# A New Efficient Explicit Deferred Correction Framework: Analysis and Applications to Hyperbolic PDEs and Adaptivity

**DOI:** 10.1007/s42967-023-00294-6

**Published:** 2023-09-12

**Authors:** Lorenzo Micalizzi, Davide Torlo

**Affiliations:** 1 Institute of Mathematics, University of Zurich, Winterthurerstrasse 190, Zurich, 8057 Switzerland; 2 SISSA mathLab, SISSA, via Bonomea 265, Trieste, 34136 Italy

**Keywords:** Efficient deferred correction (DeC), Arbitrary high order, Stability, Adaptive methods, Hyperbolic PDEs, 65M12, 65L20

## Abstract

The deferred correction (DeC) is an iterative procedure, characterized by increasing the accuracy at each iteration, which can be used to design numerical methods for systems of ODEs. The main advantage of such framework is the automatic way of getting arbitrarily high order methods, which can be put in the Runge-Kutta (RK) form. The drawback is the larger computational cost with respect to the most used RK methods. To reduce such cost, in an explicit setting, we propose an efficient modification: we introduce interpolation processes between the DeC iterations, decreasing the computational cost associated to the low order ones. We provide the Butcher tableaux of the new modified methods and we study their stability, showing that in some cases the computational advantage does not affect the stability. The flexibility of the novel modification allows nontrivial applications to PDEs and construction of adaptive methods. The good performances of the introduced methods are broadly tested on several benchmarks both in ODE and PDE contexts.

**Electronic Supplementary Material:**

The online version of this article (10.1007/s42967-023-00294-6) contains supplementary material, which is available to authorized users.

## Introduction

A huge amount of phenomena in many different fields can be modeled through ODEs and PDEs, whose analytical solutions are usually not available, hence, many numerical methods have been developed to approximate such solutions. Indeed, the higher the accuracy needed in the approximation, the more expensive the associated numerical simulations are in terms of the computational time and resources employed. If, on the one hand, with modern computers the speed of the simulations has drastically improved, and on the other hand, the always stricter tolerances required by modern applications have led to massive simulations only accessible to supercomputers and, still, characterized by very long computational times. That is why any effort in reducing the computational costs of numerical simulations is of paramount importance. A classical way of reducing them is the adoption of high order methods, which allow to reach lower errors within coarse discretizations.

A wide series of arbitrarily high order methods is based on the DeC approach. Its original formulation has been firstly introduced in 1949 in [17] in a simple prediction-correction time integrator framework. A more elegant version based on spectral integration in time was introduced in 2000 [16], characterized by an iterative procedure allowing to increase the order of accuracy by one at each iteration. In 2003 [28], Minion generalized the deferred correction (DeC) framework to obtain an implicit-explicit arbitrarily high order method, with various applications to ODEs and PDEs [19, 23, 29, 30, 35]. Later on, the DeC approach has been generalized by Abgrall [2] to solve hyperbolic PDEs with high order continuous Galerkin (CG) spatial discretizations, overcoming the burden related to the mass matrix leading to numerous applications in the hyperbolic field [4, 6, 7, 14, 27]. The DeC has been also modified to preserve physical structures (positivity, entropy, moving equilibria, conservation) [3, 5, 14, 31]. Finally, in [18] it has been pointed out that DeC and ADER methods are very similar iterative time integrators and, when restricted to ODEs, they can be written as RK schemes, see also [21, 36].

The clear advantage of the DeC framework is the possibility to easily increase the order of accuracy, the drawback is the expensive computational cost, due to the iterations and to the high degree of the polynomial reconstruction of the numerical solution considered in each of them. To alleviate such cost, the *ladder *strategy was proposed in implicit DeC algorithms [23, 28, 35], where the reconstruction in time increases the degree at each iteration. Between the iterations, an interpolation procedure links the different reconstructions. Though being the idea used in some works, it has never been deeply studied and analyzed, in particular, for the purely explicit DeC.

Inspired by this idea, in this work, we provide a detailed description of two novel families of efficient explicit DeC methods, based on easy modifications of existing DeC schemes. By explicitly constructing their Butcher tableaux and studying their stability, we show that in some cases the new efficient versions and the classical one have the same stability functions. Moreover, we exploit the modification to build adaptive methods that, given a certain tolerance, automatically choose the order of accuracy to reach such error in the most efficient way. We also apply the efficient modification in the context of mass matrix-free CG-DeC methods [2] for hyperbolic PDEs.

The structure of this work is the following. We start by introducing the DeC procedure in an abstract framework in Sect. 2 and as a tool for the numerical solution of ODEs systems in Sect. 3. In Sect. 4, we introduce the new families of efficient DeC methods. Then, we give their Butcher tableaux in Sect. 5 and in Sect. 6 we study in detail their linear stability. In Sect. 7, we describe the application to the numerical solution of hyperbolic problems with CG spatial discretizations avoiding mass matrices. We propose an adaptive and efficient version of the methods in Sect. 8. In Sect. 9, we present numerical results for ODEs and hyperbolic PDEs with various comparisons with the classical DeC methods. Section 10 is dedicated to the conclusions.

## Abstract DeC Formulation

We will first introduce the DeC abstract formulation proposed by Abgrall in [2]. Let us assume that we have two operators between two normed vector spaces $\left( X, \left\Vert \cdot \right\Vert _X \right)$ and $\left( Y, \left\Vert \cdot \right\Vert _Y \right)$, namely ${\mathcal {L}}_\Delta ^1,{\mathcal {L}}_\Delta ^2{:}\,X \longrightarrow Y$, associated to two discretizations of the same problem and dependent on a same discretization parameter $\Delta$. In particular, assume that ${\mathcal {L}}_\Delta ^2$ corresponds to a high order implicit discretization, while, ${\mathcal {L}}_\Delta ^1$ corresponds to a low order explicit one. We would like to solve ${\mathcal {L}}_\Delta ^2$, i.e., finding $\underline{\varvec{u\hspace{-1.66656pt}}\hspace{1.66656pt}}_\Delta \in X$ such that ${\mathcal {L}}_\Delta ^2(\underline{\varvec{u\hspace{-1.66656pt}}\hspace{1.66656pt}}_\Delta )=\varvec{0\hspace{-1.66656pt}}\hspace{1.66656pt}_Y$, to get a high order approximation of the solution to the original problem, but this is not easy because of its implicit character. Instead, the low order explicit operator ${\mathcal {L}}_\Delta ^1$ is very easy to solve and, more in general, we assume that it is easy to solve ${\mathcal {L}}_\Delta ^1(\underline{\varvec{u\hspace{-1.66656pt}}\hspace{1.66656pt}})=\underline{\varvec{r\hspace{-1.66656pt}}\hspace{1.66656pt}}$ with $\underline{\varvec{r\hspace{-1.66656pt}}\hspace{1.66656pt}} \in Y$ given, but the associated accuracy is not sufficient for our intended goals. In the next theorem, we will provide a simple recipe to get an arbitrary high order approximation of the solution of ${\mathcal {L}}_\Delta ^2$ by combining the operators ${\mathcal {L}}_\Delta ^1$ and ${\mathcal {L}}_\Delta ^2$ in an easy iterative procedure.

### Theorem 1

(DeC accuracy) Let the following hypotheses hold:

i) **existence of a unique solution to** ${\mathcal {L}}_\Delta ^2$
  $\exists ! \,\underline{\varvec{u\hspace{-1.66656pt}}\hspace{1.66656pt}}_\Delta \in X$ solution of ${\mathcal {L}}_\Delta ^2$ such that ${\mathcal {L}}_\Delta ^2(\underline{\varvec{u\hspace{-1.66656pt}}\hspace{1.66656pt}}_\Delta )=\varvec{0\hspace{-1.66656pt}}\hspace{1.66656pt}_Y$;
ii) **coercivity-like property of** $${\mathcal {L}}_\Delta ^1$$$$\exists \,\alpha _1 \geqslant 0$$ independent of $$\Delta $$ such that
  1 $$\begin{aligned} \left\Vert {\mathcal {L}}_\Delta ^1(\underline{\varvec{v\hspace{-1.66656pt}}\hspace{1.66656pt}})-{\mathcal {L}}_\Delta ^1(\underline{\varvec{w\hspace{-1.66656pt}}\hspace{1.66656pt}})\right\Vert _Y \geqslant \alpha _1\left\Vert \underline{\varvec{v\hspace{-1.66656pt}}\hspace{1.66656pt}}-\underline{\varvec{w\hspace{-1.66656pt}}\hspace{1.66656pt}}\right\Vert _X, ~ \forall \underline{\varvec{v\hspace{-1.66656pt}}\hspace{1.66656pt}},\underline{\varvec{w\hspace{-1.66656pt}}\hspace{1.66656pt}}\in X; \end{aligned}$$
iii) **Lipschitz-continuity-like property of** ${\mathcal {L}}_\Delta ^1-{\mathcal {L}}_\Delta ^2$
  $\exists \, \alpha _2 \geqslant 0$ independent of $\Delta$ such that
  2 $$\begin{aligned} \left\Vert \left( {\mathcal {L}}_\Delta ^1(\underline{\varvec{v\hspace{-1.66656pt}}\hspace{1.66656pt}})\!-\!{\mathcal {L}}_\Delta ^2(\underline{\varvec{v\hspace{-1.66656pt}}\hspace{1.66656pt}})\right) \!-\!\left( {\mathcal {L}}_\Delta ^1(\underline{\varvec{w\hspace{-1.66656pt}}\hspace{1.66656pt}})\!-\!{\mathcal {L}}_\Delta ^2(\underline{\varvec{w\hspace{-1.66656pt}}\hspace{1.66656pt}})\right) \right\Vert _Y\!\leqslant \!\alpha _2 \Delta \!\left\Vert \underline{\varvec{v\hspace{-1.66656pt}}\hspace{1.66656pt}}-\underline{\varvec{w\hspace{-1.66656pt}}\hspace{1.66656pt}}\right\Vert _X, ~ \forall \underline{\varvec{v\hspace{-1.66656pt}}\hspace{1.66656pt}},\underline{\varvec{w\hspace{-1.66656pt}}\hspace{1.66656pt}}\in X. \end{aligned}$$

Then, if we iteratively define $\underline{\varvec{u\hspace{-1.66656pt}}\hspace{1.66656pt}}^{(p)}$ as the solution of

3 $$\begin{aligned} {\mathcal {L}}_\Delta ^1(\underline{\varvec{u\hspace{-1.66656pt}}\hspace{1.66656pt}}^{(p)})={\mathcal {L}}_\Delta ^1(\underline{\varvec{u\hspace{-1.66656pt}}\hspace{1.66656pt}}^{(p-1)})-{\mathcal {L}}_\Delta ^2(\underline{\varvec{u\hspace{-1.66656pt}}\hspace{1.66656pt}}^{(p-1)}), \quad p=1,\cdots ,P, \end{aligned}$$

we have that

4 $$\begin{aligned} \left\Vert \underline{\varvec{u\hspace{-1.66656pt}}\hspace{1.66656pt}}^{(P)}-\underline{\varvec{u\hspace{-1.66656pt}}\hspace{1.66656pt}}_\Delta \right\Vert _X \leqslant \left( \Delta \frac{\alpha _2}{\alpha _1} \right) ^P\left\Vert \underline{\varvec{u\hspace{-1.66656pt}}\hspace{1.66656pt}}^{(0)}-\underline{\varvec{u\hspace{-1.66656pt}}\hspace{1.66656pt}}_\Delta \right\Vert _X. \end{aligned}$$

### Proof

The proof relies on a direct use of the hypotheses. In particular, we have

5a $$\begin{aligned} \left\Vert \underline{\varvec{u\hspace{-1.66656pt}}\hspace{1.66656pt}}^{(P)}-\underline{\varvec{u\hspace{-1.66656pt}}\hspace{1.66656pt}}_\Delta \right\Vert _X&\leqslant \frac{1}{\alpha _1} \left\Vert {\mathcal {L}}_\Delta ^1(\underline{\varvec{u\hspace{-1.66656pt}}\hspace{1.66656pt}}^{(P)})-{\mathcal {L}}_\Delta ^1(\underline{\varvec{u\hspace{-1.66656pt}}\hspace{1.66656pt}}_\Delta )\right\Vert _Y \end{aligned}$$

5b $$\begin{aligned}&= \frac{1}{\alpha _1} \left\Vert {\mathcal {L}}_\Delta ^1(\underline{\varvec{u\hspace{-1.66656pt}}\hspace{1.66656pt}}^{(P-1)})-{\mathcal {L}}_\Delta ^2(\underline{\varvec{u\hspace{-1.66656pt}}\hspace{1.66656pt}}^{(P-1)})-{\mathcal {L}}_\Delta ^1(\underline{\varvec{u\hspace{-1.66656pt}}\hspace{1.66656pt}}_\Delta )\right\Vert _Y \end{aligned}$$

5c $$\begin{aligned}&=\frac{1}{\alpha _1} \left\Vert {\mathcal {L}}_\Delta ^1(\underline{\varvec{u\hspace{-1.66656pt}}\hspace{1.66656pt}}^{(P-1)})-{\mathcal {L}}_\Delta ^2(\underline{\varvec{u\hspace{-1.66656pt}}\hspace{1.66656pt}}^{(P-1)})-{\mathcal {L}}_\Delta ^1(\underline{\varvec{u\hspace{-1.66656pt}}\hspace{1.66656pt}}_\Delta )+{\mathcal {L}}_\Delta ^2(\underline{\varvec{u\hspace{-1.66656pt}}\hspace{1.66656pt}}_\Delta )\right\Vert _Y \end{aligned}$$

5d $$\begin{aligned}&\leqslant \Delta \frac{\alpha _2}{\alpha _1}\left\Vert \underline{\varvec{u\hspace{-1.66656pt}}\hspace{1.66656pt}}^{(P-1)}-\underline{\varvec{u\hspace{-1.66656pt}}\hspace{1.66656pt}}_\Delta \right\Vert _X, \end{aligned}$$

where in (5a) we have used (1), in (5b) the definition of the DeC iteration (3), in (5c) the fact that ${\mathcal {L}}_\Delta ^2(\underline{\varvec{u\hspace{-1.66656pt}}\hspace{1.66656pt}}_\Delta )=\varvec{0\hspace{-1.66656pt}}\hspace{1.66656pt}_Y$ and, finally, in (5d) we have used (2). By repeating these calculations recursively we get the desired result.

Let us remark that, due to our assumption on the operator ${\mathcal {L}}_\Delta ^1$, the updating formula (3) represents a simple explicit recipe to approximate arbitrarily well the solution $\underline{\varvec{u\hspace{-1.66656pt}}\hspace{1.66656pt}}_\Delta$ of ${\mathcal {L}}_\Delta ^2$. The convergence for $P \rightarrow +\infty$ is ensured independently of the starting vector $\underline{\varvec{u\hspace{-1.66656pt}}\hspace{1.66656pt}}^{(0)}$ provided that $\Delta \frac{\alpha _2}{\alpha _1}<1$. The coefficients $\alpha _1$ and $\alpha _2$ can be computed once the operators ${\mathcal {L}}_\Delta ^1$ and ${\mathcal {L}}_\Delta ^2$ are defined. In the next sections, we will provide such definitions for different DeC ODE solvers, and the convergence constraint imposed by $\Delta \frac{\alpha _2}{\alpha _1}<1$ will sum up to a classical timestep restriction for explicit methods.

If the solution $\underline{\varvec{u\hspace{-1.66656pt}}\hspace{1.66656pt}}_\Delta$ of ${\mathcal {L}}_\Delta ^2$ is an *R*th order accurate approximation of the exact solution $\underline{\varvec{u\hspace{-1.66656pt}}\hspace{1.66656pt}}^{{\text{ex}}}$ of the original problem to which the operators are associated, it does not make sense to approximate $\underline{\varvec{u\hspace{-1.66656pt}}\hspace{1.66656pt}}_\Delta$ with the accuracy higher than *R*, as we are actually interested in $\underline{\varvec{u\hspace{-1.66656pt}}\hspace{1.66656pt}}^{{\text{ex}}}$. In particular, thanks to the accuracy estimate (4), if $\underline{\varvec{u\hspace{-1.66656pt}}\hspace{1.66656pt}}^{(0)}$ is an $O(\Delta )$-approximation of $\underline{\varvec{u\hspace{-1.66656pt}}\hspace{1.66656pt}}^{{\text{ex}}}$, the optimal choice is $P=R$, i.e., the optimal number of iterations coincides with the accuracy of the operator ${\mathcal {L}}_\Delta ^2$. Any further iteration results in a waste of computational resources.

In the following, we will characterize the operators ${\mathcal {L}}_\Delta ^1$ and ${\mathcal {L}}_\Delta ^2$ for some DeC ODEs solvers, explicitly writing the associated updating formulas. To provide a clearer understanding of the methods, we also report their more classical formulation, in Appendix A, in terms of residual and error functions [16]. However, we will stick to Abgrall’s formulation [2] for its compactness, the possibility to directly work on the solution and its flexibility, which allows for applications to more general contexts, such as structure preserving methods [3, 5, 14, 31], mass-matrix free finite element methods [2, 4, 6], ADER-DG methods [18, 25]. All these generalizations and the efficient modifications that we present in this paper are straightforward in Abgrall’s formulation, while they are more involved in the classical DeC framework.

## The DeC for Systems of ODEs

We want to solve the Cauchy problem

6 $$\begin{aligned} {\left\{ \begin{array}{ll} \frac{{\rm{d}}}{{\rm{d}}t}\varvec{u\hspace{-1.66656pt}}\hspace{1.66656pt}(t) = \varvec{G\hspace{-1.66656pt}}\hspace{1.66656pt}(t,\varvec{u\hspace{-1.66656pt}}\hspace{1.66656pt}(t)),\quad t\in [0,T], \\ \varvec{u\hspace{-1.66656pt}}\hspace{1.66656pt}(0)=\varvec{z\hspace{-1.66656pt}}\hspace{1.66656pt} \end{array}\right. } \end{aligned}$$

with $\varvec{u\hspace{-1.66656pt}}\hspace{1.66656pt}(t) \in {\mathbb {R}}^Q$, $\varvec{z\hspace{-1.66656pt}}\hspace{1.66656pt}\in {\mathbb {R}}^Q$ and $\varvec{G\hspace{-1.66656pt}}\hspace{1.66656pt}{:} \,{\mathbb {R}}^+_0 \times {\mathbb {R}}^Q \rightarrow {\mathbb {R}}^Q$ a continuous map Lipschitz continuous with respect to $\varvec{u\hspace{-1.66656pt}}\hspace{1.66656pt}$ uniformly with respect to *t* with a Lipschitz constant *L*, which ensures the existence of a unique solution. We will present two explicit DeC methods for the numerical solution of such a problem, which are based on approximations of its integral form.

- bDeC, which was introduced originally in [24] in a more general family of schemes, but fully exploited for its simplicity only starting from [2] in the context of Galerkin solvers for hyperbolic PDEs without the mass matrix. In this method, the integral form is approximated on *“big” *intervals, hence the name bDeC.
- sDeC, which has a longer history [16] and more developments [19, 22, 28, 35]. In this method, the integral form is approximated on *“small” *intervals, hence the name sDeC.

Then, we will consider a general family of DeC methods, $\alpha$DeC, depending on a parameter $\alpha$, which contains both the previously described formulations as particular cases, as described in [24].

We assume a one-step method setting: at each time interval $[t_n,t_{n+1}]$, we assume to know $\varvec{u\hspace{-1.66656pt}}\hspace{1.66656pt}_{n}\approx \varvec{u\hspace{-1.66656pt}}\hspace{1.66656pt}(t_{n})$ and we look for $\varvec{u\hspace{-1.66656pt}}\hspace{1.66656pt}_{n+1}\approx \varvec{u\hspace{-1.66656pt}}\hspace{1.66656pt}(t_{n+1})$. In particular, as in the context of a general consistency analysis, we assume $\varvec{u\hspace{-1.66656pt}}\hspace{1.66656pt}_{n}=\varvec{u\hspace{-1.66656pt}}\hspace{1.66656pt}(t_{n})$. In this context, the parameter $\Delta$ of the DeC is the step size $\Delta t=t_{n+1}-t_{n}$. A more traditional but equivalent formulation of bDeC and sDeC in terms of error and residual functions [8, 9, 12, 13] is reported in Appendix A.

### bDeC

In the generic time step $[t_n,t_n+\Delta t]$, we introduce $M+1$ subtimenodes $t_n=t^0<t^1<\cdots <t^M=t_n+\Delta t.$ Several choices of subtimenodes are possible, but for the following discussion we will consider equispaced ones. In the numerical tests, we will also present results obtained with Gauss-Lobatto (GL) subtimenodes [16, 18, 31], which can obtain a higher accuracy for a fixed number of subtimenodes. We will refer to $\varvec{u\hspace{-1.66656pt}}\hspace{1.66656pt}(t^m)$ as the exact solution in the subtimenode $t^m$ and to $\varvec{u\hspace{-1.66656pt}}\hspace{1.66656pt}^m$ as the approximation of the solution in the same subtimenode. Just for the first subtimenode, we set $\varvec{u\hspace{-1.66656pt}}\hspace{1.66656pt}^0:=\varvec{u\hspace{-1.66656pt}}\hspace{1.66656pt}_n$.

The bDeC method is based on the integral version of the ODE (6) in each interval $[t^0,t^m]$, which reads

7 $$\begin{aligned} \varvec{u\hspace{-1.66656pt}}\hspace{1.66656pt}(t^m)-\varvec{u\hspace{-1.66656pt}}\hspace{1.66656pt}^0-\int _{t^0}^{t^m}\varvec{G\hspace{-1.66656pt}}\hspace{1.66656pt}(t,\varvec{u\hspace{-1.66656pt}}\hspace{1.66656pt}(t))\textrm{d}t=\varvec{0\hspace{-1.66656pt}}\hspace{1.66656pt}, \quad m=1,\cdots ,M. \end{aligned}$$

Starting from this formulation, we define the high order operator ${\mathcal {L}}_{\Delta }^2$ and the low order operator ${\mathcal {L}}_{\Delta }^1$. We define ${\mathcal {L}}_{\Delta }^2{:}\,{\mathbb {R}}^{(M\times Q)}\rightarrow {\mathbb {R}}^{(M\times Q)}$ by approximating the function $\varvec{G\hspace{-1.66656pt}}\hspace{1.66656pt}$ in (7) with a high order interpolation via the Lagrange polynomials $\psi ^\ell$ of degree *M* associated to the $M+1$ subtimenodes and exact integration of such polynomials

8 $$\begin{aligned} {\mathcal {L}}_{\Delta }^2(\underline{\varvec{u\hspace{-1.66656pt}}\hspace{1.66656pt}}) = \begin{pmatrix} \varvec{u\hspace{-1.66656pt}}\hspace{1.66656pt}^1-\varvec{u\hspace{-1.66656pt}}\hspace{1.66656pt}^0-\Delta t \sum _{\ell =0}^{M} \theta ^1_\ell \varvec{G\hspace{-1.66656pt}}\hspace{1.66656pt}(t^\ell ,\varvec{u\hspace{-1.66656pt}}\hspace{1.66656pt}^\ell )\\ \vdots \\ \varvec{u\hspace{-1.66656pt}}\hspace{1.66656pt}^M-\varvec{u\hspace{-1.66656pt}}\hspace{1.66656pt}^0-\Delta t \sum _{\ell =0}^{M} \theta ^M_\ell \varvec{G\hspace{-1.66656pt}}\hspace{1.66656pt}(t^\ell ,\varvec{u\hspace{-1.66656pt}}\hspace{1.66656pt}^\ell )\\ \end{pmatrix} \text { with } \underline{\varvec{u\hspace{-1.66656pt}}\hspace{1.66656pt}}=\left( \begin{array}{ccc} \varvec{u\hspace{-1.66656pt}}\hspace{1.66656pt}^1\\ \vdots \\ \varvec{u\hspace{-1.66656pt}}\hspace{1.66656pt}^M \end{array} \right) , \end{aligned}$$

where the normalized coefficients $\theta ^m_\ell :=\frac{1}{\Delta t} \int _{t^0}^{t^m}\psi ^\ell (t){\rm{d}}t$ do not depend on $\Delta t$. This leads to the definition of the spaces $X=Y:={\mathbb {R}}^{M\times Q}$ of Sect. 2. Let us remark that ${\mathcal {L}}_{\Delta }^2$ is defined on the *M* components $\varvec{u\hspace{-1.66656pt}}\hspace{1.66656pt}^m\in {\mathbb {R}}^Q$ corresponding to the subtimenodes where the solution is unknown, while $\varvec{u\hspace{-1.66656pt}}\hspace{1.66656pt}^0$ is an intrinsic datum of the operator. The generic *m*th component ${\mathcal {L}}_{\Delta }^{2,m}(\underline{\varvec{u\hspace{-1.66656pt}}\hspace{1.66656pt}})=\varvec{0\hspace{-1.66656pt}}\hspace{1.66656pt}$ of the global problem ${\mathcal {L}}_{\Delta }^{2}(\underline{\varvec{u\hspace{-1.66656pt}}\hspace{1.66656pt}})=\varvec{0\hspace{-1.66656pt}}\hspace{1.66656pt}$ corresponds to a high order discretization of (7). In particular, for equispaced subtimenodes, we have that if $\varvec{u\hspace{-1.66656pt}}\hspace{1.66656pt}^m$ is the *m*th component of the solution of (8), then, it is an $(M+1)$th order accurate approximation of $\varvec{u\hspace{-1.66656pt}}\hspace{1.66656pt}(t^m)$. The proof is based on a fixed-point argument and can be found in the supplementary material. It is worth noting that ${\mathcal {L}}_{\Delta }^2(\underline{\varvec{u\hspace{-1.66656pt}}\hspace{1.66656pt}})=\varvec{0\hspace{-1.66656pt}}\hspace{1.66656pt}$ coincides with an implicit RK method with *M* stages, e.g., when choosing GL subtimenodes one obtains the LobattoIIIA methods.

The definition of the low order explicit operator ${\mathcal {L}}_{\Delta }^1{:} \,{\mathbb {R}}^{(M \times Q)} \rightarrow {\mathbb {R}}^{(M \times Q)}$ is based on a first order explicit Euler discretization of (7) leading to

9 $$\begin{aligned} {\mathcal {L}}_{\Delta }^1(\underline{\varvec{u\hspace{-1.66656pt}}\hspace{1.66656pt}}) = \begin{pmatrix} \varvec{u\hspace{-1.66656pt}}\hspace{1.66656pt}^1-\varvec{u\hspace{-1.66656pt}}\hspace{1.66656pt}^0-\Delta t \beta ^1 \varvec{G\hspace{-1.66656pt}}\hspace{1.66656pt}(t^0,\varvec{u\hspace{-1.66656pt}}\hspace{1.66656pt}^0)\\ \vdots \\ \varvec{u\hspace{-1.66656pt}}\hspace{1.66656pt}^M-\varvec{u\hspace{-1.66656pt}}\hspace{1.66656pt}^0-\Delta t \beta ^M \varvec{G\hspace{-1.66656pt}}\hspace{1.66656pt}(t^0,\varvec{u\hspace{-1.66656pt}}\hspace{1.66656pt}^0)\\ \end{pmatrix}, \end{aligned}$$

where the normalized coefficients $\beta ^m=\frac{t^m-t^0}{\Delta t}$ are determined only by the distribution of the subtimenodes. The generic *m*th component ${\mathcal {L}}_{\Delta }^{1,m}(\underline{\varvec{u\hspace{-1.66656pt}}\hspace{1.66656pt}})=\varvec{0\hspace{-1.66656pt}}\hspace{1.66656pt}$ of ${\mathcal {L}}_{\Delta }^{1}(\underline{\varvec{u\hspace{-1.66656pt}}\hspace{1.66656pt}})=\varvec{0\hspace{-1.66656pt}}\hspace{1.66656pt}$ corresponds to the explicit Euler discretization of (7), hence, it is first order accurate and any system ${\mathcal {L}}_{\Delta }^1(\underline{\varvec{u\hspace{-1.66656pt}}\hspace{1.66656pt}})=\underline{\varvec{r\hspace{-1.66656pt}}\hspace{1.66656pt}}$ can be readily solved for a given $\underline{\varvec{r\hspace{-1.66656pt}}\hspace{1.66656pt}}\in {\mathbb {R}}^{M\times Q}$.

**Fig. 1:**
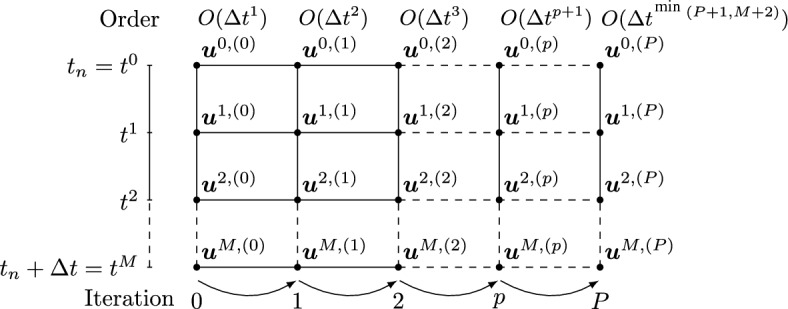
Sketch of the DeC iterative process for equispaced subtimenodes

The operators ${\mathcal {L}}_{\Delta }^1$ and ${\mathcal {L}}_{\Delta }^2$ fulfill the hypotheses required to apply the DeC procedure, the proofs can be found in the supplementary material. In particular, we highlight that $\alpha _1=1$, while $\alpha _2=L \cdot \max _{m=1,\cdots ,M}\sum _{\ell =1}^M\vert \theta _\ell ^m\vert$.

Let us now characterize the updating formula (3) to this setting. The vector $\underline{\varvec{u\hspace{-1.66656pt}}\hspace{1.66656pt}}^{(p)} \in {\mathbb {R}}^{(M\times Q)}$ is, in this case, made by *M* components $\varvec{u\hspace{-1.66656pt}}\hspace{1.66656pt}^{m,(p)}\in {\mathbb {R}}^Q$, associated to the subtimenodes $t^m$ $m=1,\cdots ,M$ in which the solution is unknown, while we set $\varvec{u\hspace{-1.66656pt}}\hspace{1.66656pt}^{0,(p)}:=\varvec{u\hspace{-1.66656pt}}\hspace{1.66656pt}_n$ for all *p*. Then, (3) gives

10 $$\begin{aligned} \varvec{u\hspace{-1.66656pt}}\hspace{1.66656pt}^{m,(p)} = \varvec{u\hspace{-1.66656pt}}\hspace{1.66656pt}^0+\Delta t \sum _{\ell =0}^{M} \theta ^m_\ell \varvec{G\hspace{-1.66656pt}}\hspace{1.66656pt}(t^\ell ,\varvec{u\hspace{-1.66656pt}}\hspace{1.66656pt}^{\ell ,(p-1)}), \quad m=1,\cdots ,M. \end{aligned}$$

The starting vector $\underline{\varvec{u\hspace{-1.66656pt}}\hspace{1.66656pt}}^{(0)}$ for our iterative procedure is chosen as $\varvec{u\hspace{-1.66656pt}}\hspace{1.66656pt}^{m,(0)}:=\varvec{u\hspace{-1.66656pt}}\hspace{1.66656pt}_n$ for all *m*. At the end of the iteration process, we set $\varvec{u\hspace{-1.66656pt}}\hspace{1.66656pt}_{n+1}:= \varvec{u\hspace{-1.66656pt}}\hspace{1.66656pt}^{M,(P)}$. A graphical sketch of the updating process is shown in Fig. 1. As said in Sect. 2, the optimal number of iterations depends on the accuracy of the operator ${\mathcal {L}}_{\Delta }^2$, i.e., $P=M+1$ for equispaced subtimenodes and $P=2M$ for GL ones. Further iterations would not increase the order of accuracy of the method. On the other hand, to build a *P*th order method, the optimal choice consists of *P* iterations with $M=P-1$ for equispaced and $M=\left\lceil {\frac{P}{2}} \right\rceil$ for GL subtimenodes.

### sDeC

The sDeC operators differ from the bDeC ones by the “smaller” intervals considered to obtain the integral version of the ODE. In fact, adopting the previous definition of the subtimenodes, the sDeC method is based on the integral version of (6) over the intervals $[t^{m-1},t^m]$ for $m=1,\cdots ,M$. This leads to the following definition of the operators ${\mathcal {L}}_{\Delta }^1, {\mathcal {L}}_{\Delta }^2{:} \,{\mathbb {R}}^{(M \times Q)} \rightarrow {\mathbb {R}}^{(M \times Q)},$

11 $$\begin{aligned} {\mathcal {L}}_{\Delta }^{1,m}(\underline{\varvec{u\hspace{-1.66656pt}}\hspace{1.66656pt}}):=\varvec{u\hspace{-1.66656pt}}\hspace{1.66656pt}^m-\varvec{u\hspace{-1.66656pt}}\hspace{1.66656pt}^{m-1}-\Delta t \gamma ^m \varvec{G\hspace{-1.66656pt}}\hspace{1.66656pt}(t^{m-1},\varvec{u\hspace{-1.66656pt}}\hspace{1.66656pt}^{m-1}) \qquad \text {for }m=1,\cdots ,M, \end{aligned}$$

12 $$\begin{aligned} {\mathcal {L}}_{\Delta }^{2,m}(\underline{\varvec{u\hspace{-1.66656pt}}\hspace{1.66656pt}}):=\varvec{u\hspace{-1.66656pt}}\hspace{1.66656pt}^m-\varvec{u\hspace{-1.66656pt}}\hspace{1.66656pt}^{m-1}-\Delta t \sum _{\ell =0}^{M} \delta ^m_\ell \varvec{G\hspace{-1.66656pt}}\hspace{1.66656pt}(t^\ell ,\varvec{u\hspace{-1.66656pt}}\hspace{1.66656pt}^\ell ) \qquad \text {for }m=1,\cdots ,M \end{aligned}$$

with $\gamma ^m=\frac{t^m-t^{m-1}}{\Delta t}$ and $\delta ^m_\ell :=\frac{1}{\Delta t}\int _{t^{m-1}}^{t^m}\psi ^\ell (t){\rm{d}}t$ normalized coefficients. As before, ${\mathcal {L}}_{\Delta }^{1,m}(\underline{\varvec{u\hspace{-1.66656pt}}\hspace{1.66656pt}})=\varvec{0\hspace{-1.66656pt}}\hspace{1.66656pt}$ is a first order explicit discretization, while, ${\mathcal {L}}_{\Delta }^{2,m}(\underline{\varvec{u\hspace{-1.66656pt}}\hspace{1.66656pt}})=\varvec{0\hspace{-1.66656pt}}\hspace{1.66656pt}$ is a high order implicit one and, further, we have $\varvec{u\hspace{-1.66656pt}}\hspace{1.66656pt}^0:=\varvec{u\hspace{-1.66656pt}}\hspace{1.66656pt}_n$.

Differently from the previous formulation, in this case we cannot solve the operator ${\mathcal {L}}_{\Delta }^1$ in all its components at the same time but we have to do it component by component from $\varvec{u\hspace{-1.66656pt}}\hspace{1.66656pt}^1$ to $\varvec{u\hspace{-1.66656pt}}\hspace{1.66656pt}^M$. The same holds for the general problem ${\mathcal {L}}_{\Delta }^1(\underline{\varvec{u\hspace{-1.66656pt}}\hspace{1.66656pt}})=\underline{\varvec{r\hspace{-1.66656pt}}\hspace{1.66656pt}}$ for a fixed $\underline{\varvec{r\hspace{-1.66656pt}}\hspace{1.66656pt}}\in {\mathbb {R}}^{(M \times Q)}$. However, still the computation of its solution can be performed explicitly.

Let us characterize the updating formula (3) to this context. The explicit character of the operator ${\mathcal {L}}_{\Delta }^1$ leads to an explicit recipe for the computation of $\underline{\varvec{u\hspace{-1.66656pt}}\hspace{1.66656pt}}^{(p)}$ whose components, in this case, must be computed in an increasing order

13 $$ \begin{aligned} \varvec{u\hspace{-1.66656pt}}\hspace{1.66656pt}^{m,(p)}&=\varvec{u\hspace{-1.66656pt}}\hspace{1.66656pt}^{m-1,(p)}+\Delta t \gamma ^m \left( \varvec{G\hspace{-1.66656pt}}\hspace{1.66656pt}(t^{m-1},\varvec{u\hspace{-1.66656pt}}\hspace{1.66656pt}^{m-1,(p)})- \varvec{G\hspace{-1.66656pt}}\hspace{1.66656pt}(t^{m-1},\varvec{u\hspace{-1.66656pt}}\hspace{1.66656pt}^{m-1,(p-1)})\right) \\ {}&\quad +\Delta t \sum _{\ell =0}^{M} \delta ^m_\ell \varvec{G\hspace{-1.66656pt}}\hspace{1.66656pt}(t^\ell ,\varvec{u\hspace{-1.66656pt}}\hspace{1.66656pt}^{\ell ,(p-1)}). \end{aligned}$$

With recursive substitutions, (13) can be equivalently written as

14 $$\begin{aligned} \varvec{u\hspace{-1.66656pt}}\hspace{1.66656pt}^{m,(p)}&=\varvec{u\hspace{-1.66656pt}}\hspace{1.66656pt}^{0}+\Delta t \sum _{\ell =0}^{m-1} \gamma ^{\ell +1} \left( \varvec{G\hspace{-1.66656pt}}\hspace{1.66656pt}(t^{\ell },\varvec{u\hspace{-1.66656pt}}\hspace{1.66656pt}^{\ell ,(p)})-\varvec{G\hspace{-1.66656pt}}\hspace{1.66656pt}(t^{\ell },\varvec{u\hspace{-1.66656pt}}\hspace{1.66656pt}^{\ell ,(p-1)}) \right) \\&\quad +\Delta t\sum _{r=1}^m \sum _{\ell =0}^{M} \delta ^r_\ell \varvec{G\hspace{-1.66656pt}}\hspace{1.66656pt}(t^\ell ,\varvec{u\hspace{-1.66656pt}}\hspace{1.66656pt}^{\ell ,(p-1)}). \end{aligned} $$

Now, let us focus on the last term of (14). Exchanging the sums over *r* and $\ell$, thanks to the fact that $\sum _{r=1}^m \delta ^r_\ell =\theta ^m_\ell$, we have

15 $$ \begin{aligned} \varvec{u\hspace{-1.66656pt}}\hspace{1.66656pt}^{m,(p)}&=\varvec{u\hspace{-1.66656pt}}\hspace{1.66656pt}^{0}+ \Delta t \sum _{\ell =0}^{m-1} \gamma ^{\ell +1} \left( \varvec{G\hspace{-1.66656pt}}\hspace{1.66656pt}(t^{\ell },\varvec{u\hspace{-1.66656pt}}\hspace{1.66656pt}^{\ell ,(p)})-\varvec{G\hspace{-1.66656pt}}\hspace{1.66656pt}(t^{\ell },\varvec{u\hspace{-1.66656pt}}\hspace{1.66656pt}^{\ell ,(p-1)}) \right) \\&\quad +\Delta t \sum _{\ell =0}^{M} \theta ^m_\ell \varvec{G\hspace{-1.66656pt}}\hspace{1.66656pt}(t^\ell ,\varvec{u\hspace{-1.66656pt}}\hspace{1.66656pt}^{\ell ,(p-1)}), \end{aligned}$$

which allows to explicitly compute all the components $\varvec{u\hspace{-1.66656pt}}\hspace{1.66656pt}^{m,(p)}$ in sequence from $m=1$ to $m=M$, in opposition to bDeC where a parallel strategy can be adopted. For what concerns the accuracy of the method and the optimal number of iterations, one can refer to what already said in the context of the bDeC formulation.

Let us observe that the sDeC method is equivalent to the DeC method presented in [16] in terms of residuals and error functions. We show the equivalence in Appendix A.

### A General Family of DeC Methods, $\alpha$DeC

Following [21], we can construct a family of schemes dependent on a single parameter $\alpha \in [0,1]$ by a convex combination of the updating formulas of bDeC (10) and sDeC (15):

16 $$\begin{aligned}  \varvec{u\hspace{-1.66656pt}}\hspace{1.66656pt}^{m,(p)}&=\varvec{u\hspace{-1.66656pt}}\hspace{1.66656pt}^0 +\Delta t \sum _{\ell =0}^{M} \theta ^m_\ell \varvec{G\hspace{-1.66656pt}}\hspace{1.66656pt}(t^\ell ,\varvec{u\hspace{-1.66656pt}}\hspace{1.66656pt}^{\ell ,(p-1)})\\&\quad + \alpha \left[ \Delta t \sum _{\ell =0}^{m-1} \gamma ^{\ell +1} \left( \varvec{G\hspace{-1.66656pt}}\hspace{1.66656pt}(t^{\ell },\varvec{u\hspace{-1.66656pt}}\hspace{1.66656pt}^{\ell ,(p)})-\varvec{G\hspace{-1.66656pt}}\hspace{1.66656pt}(t^{\ell },\varvec{u\hspace{-1.66656pt}}\hspace{1.66656pt}^{\ell ,(p-1)}) \right) \right] . \end{aligned}$$

Through (16), it is possible to explicitly compute iteration by iteration the different components $\varvec{u\hspace{-1.66656pt}}\hspace{1.66656pt}^{m,(p)}$ starting from $m=1$ until *M*. Of course, when $\alpha =0$ we retrieve the bDeC formulation, while for $\alpha =1$ we get the sDeC one.

#### Matrix Formulation

We will now introduce a compact matrix-formulation of the presented methods. For convenience, we will now introduce the vectors containing as components the quantities related to all the subtimenodes including the initial one, even if $\varvec{u\hspace{-1.66656pt}}\hspace{1.66656pt}^0=\varvec{u\hspace{-1.66656pt}}\hspace{1.66656pt}_n$ is never changed along the iterations and it is not an input of the operators previously described. To avoid confusion, we refer to the vectors not containing such a component with the small letter and to the vectors containing it with the capital letter, i.e.,

17 $$\begin{aligned} \underline{\varvec{u\hspace{-1.66656pt}}\hspace{1.66656pt}}^{(p)}= \, \left( \begin{array}{ccc} \varvec{u\hspace{-1.66656pt}}\hspace{1.66656pt}^{1,(p)}\\ \vdots \\ \varvec{u\hspace{-1.66656pt}}\hspace{1.66656pt}^{M,(p)} \end{array} \right) , \quad \underline{\varvec{U\hspace{-1.66656pt}}\hspace{1.66656pt}}^{(p)}=\left( \begin{array}{ccc} \varvec{u\hspace{-1.66656pt}}\hspace{1.66656pt}^{0}\\ \underline{\varvec{u\hspace{-1.66656pt}}\hspace{1.66656pt}}^{(p)} \end{array} \right) . \end{aligned}$$

We will also denote the component-wise application of $\varvec{G\hspace{-1.66656pt}}\hspace{1.66656pt}$ to the vectors $\underline{\varvec{u\hspace{-1.66656pt}}\hspace{1.66656pt}}^{(p)}$ and $\underline{\varvec{U\hspace{-1.66656pt}}\hspace{1.66656pt}}^{(p)}$ by

18 $$\begin{aligned} \underline{\varvec{G\hspace{-1.66656pt}}\hspace{1.66656pt}}(\underline{\varvec{u\hspace{-1.66656pt}}\hspace{1.66656pt}}^{(p)})=\left( \begin{array}{ccc} \varvec{G\hspace{-1.66656pt}}\hspace{1.66656pt}(t^{1},\varvec{u\hspace{-1.66656pt}}\hspace{1.66656pt}^{1,(p)})\\ \vdots \\ \varvec{G\hspace{-1.66656pt}}\hspace{1.66656pt}(t^{M},\varvec{u\hspace{-1.66656pt}}\hspace{1.66656pt}^{M,(p)}) \end{array} \right) , \quad \underline{\varvec{G\hspace{-1.66656pt}}\hspace{1.66656pt}}(\underline{\varvec{U\hspace{-1.66656pt}}\hspace{1.66656pt}}^{(p)})=\left( \begin{array}{ccc} \varvec{G\hspace{-1.66656pt}}\hspace{1.66656pt}(t^{0},\varvec{u\hspace{-1.66656pt}}\hspace{1.66656pt}^{0})\\ \underline{\varvec{G\hspace{-1.66656pt}}\hspace{1.66656pt}}(\underline{\varvec{u\hspace{-1.66656pt}}\hspace{1.66656pt}}^{(p)}) \end{array} \right) . \end{aligned}$$

With the previous definitions, it is possible to recast the general updating formula (16) in the following compact form:

19 $$\begin{aligned}  \underline{\varvec{U\hspace{-1.66656pt}}\hspace{1.66656pt}}^{(p)}&= \underline{\varvec{U\hspace{-1.66656pt}}\hspace{1.66656pt}}^{(0)}+\Delta t\Theta \underline{\varvec{G\hspace{-1.66656pt}}\hspace{1.66656pt}}(\underline{\varvec{U\hspace{-1.66656pt}}\hspace{1.66656pt}}^{(p-1)})+\Delta t \alpha \Gamma (\underline{\varvec{G\hspace{-1.66656pt}}\hspace{1.66656pt}}(\underline{\varvec{U\hspace{-1.66656pt}}\hspace{1.66656pt}}^{(p)}) -\underline{\varvec{G\hspace{-1.66656pt}}\hspace{1.66656pt}}(\underline{\varvec{U\hspace{-1.66656pt}}\hspace{1.66656pt}}^{(p-1)}))\\&= \underline{\varvec{U\hspace{-1.66656pt}}\hspace{1.66656pt}}^{(0)}+\Delta t(\Theta -\alpha \Gamma )\underline{\varvec{G\hspace{-1.66656pt}}\hspace{1.66656pt}}(\underline{\varvec{U\hspace{-1.66656pt}}\hspace{1.66656pt}}^{(p-1)})+\Delta t \alpha \Gamma \underline{\varvec{G\hspace{-1.66656pt}}\hspace{1.66656pt}}(\underline{\varvec{U\hspace{-1.66656pt}}\hspace{1.66656pt}}^{(p)}), \end{aligned} 
$$

where the vector $\underline{\varvec{U\hspace{-1.66656pt}}\hspace{1.66656pt}}^{(0)}\in {\mathbb {R}}^{((M+1)\times Q)}$ and the matrices $\Theta ,\Gamma \in {\mathbb {R}}^{(M+1)\times (M+1)}$ are defined as

20 $$\begin{aligned} \underline{\varvec{U\hspace{-1.66656pt}}\hspace{1.66656pt}}^{(0)}= \, \left( \begin{array}{ccc} \varvec{u\hspace{-1.66656pt}}\hspace{1.66656pt}_{n}\\ \vdots \\ \varvec{u\hspace{-1.66656pt}}\hspace{1.66656pt}_{n} \end{array} \right) , \, \Theta =\begin{pmatrix} 0 &{} 0 &{} \dots &{} 0 \\ \theta ^1_0 &{} \theta ^1_1 &{} \dots &{} \theta ^1_M \\ \theta ^2_0 &{} \theta ^2_1 &{} \dots &{} \theta ^2_M \\ \vdots &{} \vdots &{}  &{} \vdots \\ \theta ^M_0 &{} \theta ^M_1 &{} \dots &{} \theta ^M_M \\ \end{pmatrix}, \, \Gamma =\begin{pmatrix} 0 &{} 0 &{} \dots &{} 0 &{} 0 \\ \gamma ^1 &{} 0 &{} \dots &{} 0 &{} 0 \\ \gamma ^1 &{} \gamma ^2 &{}  &{} 0 &{} 0 \\ \vdots &{} \vdots &{}  &{} \vdots &{} \vdots \\ \gamma ^1 &{} \gamma ^2 &{} \dots &{} \gamma ^M &{} 0\\ \end{pmatrix} \end{aligned}$$

with the matrix $\Gamma$ being strictly lower-triangular, as the scheme is fully explicit. Let us observe that the first component $\varvec{u\hspace{-1.66656pt}}\hspace{1.66656pt}^{0}$ of $\underline{\varvec{U\hspace{-1.66656pt}}\hspace{1.66656pt}}^{(p)}$ is never updated. This is coherent with what we have said so far. The matrices $\Theta$ and $\Gamma$ that we have defined are referred to a scalar ODE ($Q=1$). In case one wants to adapt them to a vectorial problem, they must be block-expanded.

## Two Novel Families of DeC Methods

In this section, we will show how to construct two novel families of efficient DeC methods by introducing a modification in the $\alpha$DeC methods, first focusing on equispaced subtimenodes and then extending the idea to GL ones. The modification is based on the following observation: at any iteration $p<M+1$, we get a solution $\underline{\varvec{u\hspace{-1.66656pt}}\hspace{1.66656pt}}^{(p)}$ that is *p*th order accurate using $M+1$ subtimenodes even though only *p* would be formally sufficient to provide such accuracy. In other words, the number of subtimenodes is fixed a priori for all iterations to get the desired order of accuracy. These subtimenodes are used throughout the whole iterative process, although the formal order of accuracy, for which such nodes are required, is reached only in the final iteration. This represents indeed a waste of computational resources.

The proposed modification consists in starting with only two subtimenodes and increasing their number, iteration by iteration, matching the order of accuracy achieved in the specific iteration. In particular, we introduce intermediate interpolation processes between the iterations to retrieve the needed quantities in the new subtimenodes. The idea has been introduced in [28] for implicit methods, but without a systematic theory and related analytical study. We will present here two possible interpolation strategies which will lead to the definition of two general families of efficient DeC methods.

We will use the star symbol $*$ to refer to quantities obtained through the interpolation process. The number of subtimenodes will change iteration by iteration, therefore, it is useful to define the vector ${\underline{t}}^{(p)}:=\left( t^{0,(p)}, \cdots , t^{p,(p)} \right) ^{\rm{T}}$ of the subtimenodes in which we obtain the approximations of the solution at the *p*th iteration, with $t^{0,(p)}=t_n$ and $t^{p,(p)}=t_{n+1}$.

### $\alpha$DeCu

The $\alpha$DeCu methods are obtained from the $\alpha$DeC methods by introducing an intermediate interpolation process on the solution $\varvec{u\hspace{-1.66656pt}}\hspace{1.66656pt}(t)$ between the iterations. For convenience, we will formulate the methods in terms of the vectors $\underline{\varvec{U\hspace{-1.66656pt}}\hspace{1.66656pt}}^{(p)}$ containing the component $\varvec{u\hspace{-1.66656pt}}\hspace{1.66656pt}^0=\varvec{u\hspace{-1.66656pt}}\hspace{1.66656pt}_n$ associated to the initial subtimenode.

**Fig. 2:**
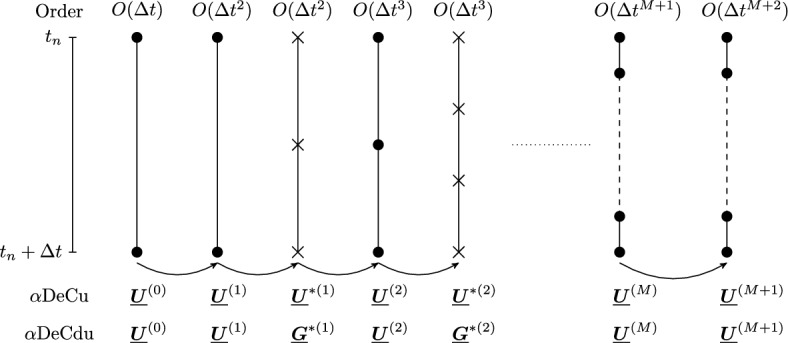
$\alpha$DeCu and $\alpha$DeCdu, sketches: dots for computed values, crosses for interpolated ones

We start with $\underline{\varvec{U\hspace{-1.66656pt}}\hspace{1.66656pt}}^{(0)}=(\varvec{u\hspace{-1.66656pt}}\hspace{1.66656pt}_n,\varvec{u\hspace{-1.66656pt}}\hspace{1.66656pt}_n)^{\rm{T}} \in {\mathbb {R}}^{(2\times Q)}$ associated to two subtimenodes, $t_n$ and $t_n+\Delta t$, and we perform the first iteration

21 $$\begin{aligned} \underline{\varvec{U\hspace{-1.66656pt}}\hspace{1.66656pt}}^{(1)} = \underline{\varvec{U\hspace{-1.66656pt}}\hspace{1.66656pt}}^{(0)}+\Delta t(\Theta ^{(1)} -\alpha \Gamma ^{(1)} )\underline{\varvec{G\hspace{-1.66656pt}}\hspace{1.66656pt}}(\underline{\varvec{U\hspace{-1.66656pt}}\hspace{1.66656pt}}^{(0)})+\Delta t \alpha \Gamma ^{(1)} \underline{\varvec{G\hspace{-1.66656pt}}\hspace{1.66656pt}}(\underline{\varvec{U\hspace{-1.66656pt}}\hspace{1.66656pt}}^{(1)}) \in {\mathbb {R}}^{(2\times Q)}. \end{aligned}$$

$\underline{\varvec{U\hspace{-1.66656pt}}\hspace{1.66656pt}}^{(1)}$ is first order accurate and it yields an $O(\Delta t^2)$-accurate reconstruction on $[t_n,t_{n+1}]$. Here, $\Gamma ^{(1)}$ and $\Theta ^{(1)}$ are the operators associated to two subtimenodes. Now, we perform the first interpolation, via a suitable interpolation matrix $H^{(1)}$, passing from two to three equispaced subtimenodes

22 $$ \begin{aligned} \underline{\varvec{U\hspace{-1.66656pt}}\hspace{1.66656pt}}^{*(1)}&= H^{(1)}\underline{\varvec{U\hspace{-1.66656pt}}\hspace{1.66656pt}}^{(1)}\\&=H^{(1)}\left[ \underline{\varvec{U\hspace{-1.66656pt}}\hspace{1.66656pt}}^{(0)}+\Delta t(\Theta ^{(1)} -\alpha \Gamma ^{(1)} )\underline{\varvec{G\hspace{-1.66656pt}}\hspace{1.66656pt}}(\underline{\varvec{U\hspace{-1.66656pt}}\hspace{1.66656pt}}^{(0)})+\Delta t \alpha \Gamma ^{(1)} \underline{\varvec{G\hspace{-1.66656pt}}\hspace{1.66656pt}}(\underline{\varvec{U\hspace{-1.66656pt}}\hspace{1.66656pt}}^{(1)})\right] \\&=\underline{\varvec{U\hspace{-1.66656pt}}\hspace{1.66656pt}}^{(0)}_3+\Delta t H^{(1)}(\Theta ^{(1)} -\alpha \Gamma ^{(1)} )\underline{\varvec{G\hspace{-1.66656pt}}\hspace{1.66656pt}}(\underline{\varvec{U\hspace{-1.66656pt}}\hspace{1.66656pt}}^{(0)})+\Delta t \alpha H^{(1)}\Gamma ^{(1)} \underline{\varvec{G\hspace{-1.66656pt}}\hspace{1.66656pt}}(\underline{\varvec{U\hspace{-1.66656pt}}\hspace{1.66656pt}}^{(1)}), \end{aligned}$$

where the last equality is due to the fact that, by consistency, the sum of the elements on the rows of the interpolation matrices $H^{(p)}$ is equal to 1. The subscript 3 has been added to $\underline{\varvec{U\hspace{-1.66656pt}}\hspace{1.66656pt}}^{(0)}_3 \in {\mathbb {R}}^{3\times Q}$ to distinguish it from the initial $\underline{\varvec{U\hspace{-1.66656pt}}\hspace{1.66656pt}}^{(0)}\in {\mathbb {R}}^{2\times Q}$. Now, we have $\underline{\varvec{U\hspace{-1.66656pt}}\hspace{1.66656pt}}^{*(1)}\in {\mathbb {R}}^{(3\times Q)}$, still first order accurate. Then, we perform the second iteration

23 $$\begin{aligned} \underline{\varvec{U\hspace{-1.66656pt}}\hspace{1.66656pt}}^{(2)} = \underline{\varvec{U\hspace{-1.66656pt}}\hspace{1.66656pt}}^{(0)}_3+\Delta t(\Theta ^{(2)} -\alpha \Gamma ^{(2)} )\underline{\varvec{G\hspace{-1.66656pt}}\hspace{1.66656pt}}(\underline{\varvec{U\hspace{-1.66656pt}}\hspace{1.66656pt}}^{*(1)})+\Delta t \alpha \Gamma ^{(2)} \underline{\varvec{G\hspace{-1.66656pt}}\hspace{1.66656pt}}(\underline{\varvec{U\hspace{-1.66656pt}}\hspace{1.66656pt}}^{(2)}), \end{aligned}$$

which gives a second order accurate approximation, i.e., an $O(\Delta t^3)$-accurate approximation. Thus, we continue with another interpolation

24 $$ \begin{aligned} \underline{\varvec{U\hspace{-1.66656pt}}\hspace{1.66656pt}}^{*(2)}&= H^{(2)}\underline{\varvec{U\hspace{-1.66656pt}}\hspace{1.66656pt}}^{(2)}\\&=H^{(2)}\left[ \underline{\varvec{U\hspace{-1.66656pt}}\hspace{1.66656pt}}^{(0)}_3+\Delta t(\Theta ^{(2)} -\alpha \Gamma ^{(2)} )\underline{\varvec{G\hspace{-1.66656pt}}\hspace{1.66656pt}}(\underline{\varvec{U\hspace{-1.66656pt}}\hspace{1.66656pt}}^{*(1)})+\Delta t \alpha \Gamma ^{(2)} \underline{\varvec{G\hspace{-1.66656pt}}\hspace{1.66656pt}}(\underline{\varvec{U\hspace{-1.66656pt}}\hspace{1.66656pt}}^{(2)})\right] \\&=\underline{\varvec{U\hspace{-1.66656pt}}\hspace{1.66656pt}}^{(0)}_4+\Delta t H^{(2)}(\Theta ^{(2)} -\alpha \Gamma ^{(2)} )\underline{\varvec{G\hspace{-1.66656pt}}\hspace{1.66656pt}}(\underline{\varvec{U\hspace{-1.66656pt}}\hspace{1.66656pt}}^{*(1)})+\Delta t \alpha H^{(2)}\Gamma ^{(2)} \underline{\varvec{G\hspace{-1.66656pt}}\hspace{1.66656pt}}(\underline{\varvec{U\hspace{-1.66656pt}}\hspace{1.66656pt}}^{(2)}), \end{aligned} $$

from which we can get $\underline{\varvec{U\hspace{-1.66656pt}}\hspace{1.66656pt}}^{(3)}$ $O(\Delta t^4)$-accurate and so on. Proceeding iteratively, at the *p*th iteration we have

25 $$\begin{aligned} \underline{\varvec{U\hspace{-1.66656pt}}\hspace{1.66656pt}}^{*(p-1)} &=\, \underline{\varvec{U\hspace{-1.66656pt}}\hspace{1.66656pt}}^{(0)}_{p+1}+\Delta t H^{(p-1)}(\Theta ^{(p-1)} -\alpha \Gamma ^{(p-1)} )\underline{\varvec{G\hspace{-1.66656pt}}\hspace{1.66656pt}}(\underline{\varvec{U\hspace{-1.66656pt}}\hspace{1.66656pt}}^{*(p-2)})\\&\quad+\Delta t \alpha H^{(p-1)}\Gamma ^{(p-1)} \underline{\varvec{G\hspace{-1.66656pt}}\hspace{1.66656pt}}(\underline{\varvec{U\hspace{-1.66656pt}}\hspace{1.66656pt}}^{(p-1)}), \end{aligned}$$

26 $$\begin{aligned}&\underline{\varvec{U\hspace{-1.66656pt}}\hspace{1.66656pt}}^{(p)} = \underline{\varvec{U\hspace{-1.66656pt}}\hspace{1.66656pt}}^{(0)}_{p+1}+\Delta t (\Theta ^{(p)} -\alpha \Gamma ^{(p)} )\underline{\varvec{G\hspace{-1.66656pt}}\hspace{1.66656pt}}(\underline{\varvec{U\hspace{-1.66656pt}}\hspace{1.66656pt}}^{*(p-1)})+\Delta t \alpha \Gamma ^{(p)} \underline{\varvec{G\hspace{-1.66656pt}}\hspace{1.66656pt}}(\underline{\varvec{U\hspace{-1.66656pt}}\hspace{1.66656pt}}^{(p)}), \end{aligned}$$

where $\underline{\varvec{U\hspace{-1.66656pt}}\hspace{1.66656pt}}^{*(p-1)} \in {\mathbb {R}}^{(p+1)\times Q}$ is $O(\Delta t^p)$-accurate, and $\underline{\varvec{U\hspace{-1.66656pt}}\hspace{1.66656pt}}^{(p)}\in {\mathbb {R}}^{(p+1)\times Q}$ is $O(\Delta t^{p+1})$-accurate. Clearly, the DeC operators $\Theta ^{(p)}$ and $\Gamma ^{(p)}$, used in the *p*th iteration, are chosen according to the dimension of involved variables.

Let us notice that $\underline{\varvec{U\hspace{-1.66656pt}}\hspace{1.66656pt}}^{(p)}\in {\mathbb {R}}^{((p+1)\times Q)}$, got at the *p*th iteration, is $O(\Delta t^{p+1})$-accurate and associated to $p+1$ subtimenodes but, actually, they would be enough to guarantee the $O(\Delta t^{p+2})$-accuracy. For this reason, if the final number of subtimenodes is fixed to be $M+1$, the optimal choice is to perform *M* iterations to reach such setting and a final $(M+1)$th iteration without interpolation to saturate the $O(\Delta t^{M+2})$-accuracy associated to the subtimenodes. In this way, we have that the interpolation is performed at each iteration except the first and the last one. Thus, the last iteration reads

27 $$\begin{aligned} \underline{\varvec{U\hspace{-1.66656pt}}\hspace{1.66656pt}}^{(M+1)}\!\!\, = \underline{\varvec{U\hspace{-1.66656pt}}\hspace{1.66656pt}}^{(0)}_{M+1}\!+\Delta t (\Theta ^{(M)}\!\! -\alpha \Gamma ^{(M)} )\underline{\varvec{G\hspace{-1.66656pt}}\hspace{1.66656pt}}(\underline{\varvec{U\hspace{-1.66656pt}}\hspace{1.66656pt}}^{(M)})\!+\Delta t \alpha \Gamma ^{(M)} \underline{\varvec{G\hspace{-1.66656pt}}\hspace{1.66656pt}}(\underline{\varvec{U\hspace{-1.66656pt}}\hspace{1.66656pt}}^{(M+1)}), \end{aligned}$$

where the matrices $\Theta ^{(M)}$ and $\Gamma ^{(M)}$ are the ones used also for the *M*th iteration. A useful sketch of the algorithm is represented in Fig. 2.

On the other hand, one could also not fix a priori the final number of subtimenodes and stop when certain conditions are met, see an example for adaptive methods in Sect. 8.

### $\alpha$DeCdu

Like the $\alpha$DeCu methods, the $\alpha$DeCdu methods are based on the introduction of an interpolation process between the iterations. In this case, the interpolated quantity is the function $\varvec{G\hspace{-1.66656pt}}\hspace{1.66656pt}(t,\varvec{u\hspace{-1.66656pt}}\hspace{1.66656pt}(t))$. The name is due to the fact that formally we interpolate $\frac{{\rm{d}}}{{\rm{d}}t}\varvec{u\hspace{-1.66656pt}}\hspace{1.66656pt}(t)=\varvec{G\hspace{-1.66656pt}}\hspace{1.66656pt}(t,\varvec{u\hspace{-1.66656pt}}\hspace{1.66656pt}(t))$.

We start with two subtimenodes, associated to $t_n$ and $t_n+\Delta t$, and $\underline{\varvec{U\hspace{-1.66656pt}}\hspace{1.66656pt}}^{(0)}\in {\mathbb {R}}^{(2\times Q)}$ and we perform the first iteration of the $\alpha$DeC method, as in (21), getting $\underline{\varvec{U\hspace{-1.66656pt}}\hspace{1.66656pt}}^{(1)}\in {\mathbb {R}}^{(2\times Q)}$, which is $O(\Delta t^2)$-accurate. Then, we can compute $\underline{\varvec{G\hspace{-1.66656pt}}\hspace{1.66656pt}}(\underline{\varvec{U\hspace{-1.66656pt}}\hspace{1.66656pt}}^{(1)})$, whose components allow to get an $O(\Delta t^2)$-accurate global reconstruction of $\varvec{G\hspace{-1.66656pt}}\hspace{1.66656pt}(t,\varvec{u\hspace{-1.66656pt}}\hspace{1.66656pt}(t))$ in the interval $[t_n,t_n+\Delta t]$ through the Lagrange interpolation. We thus perform an interpolation to retrieve the approximated values of $\varvec{G\hspace{-1.66656pt}}\hspace{1.66656pt}(t,\varvec{u\hspace{-1.66656pt}}\hspace{1.66656pt}(t))$ in three equispaced subtimenodes in the interval $[t_n,t_n+\Delta t]$, getting $\underline{\varvec{G\hspace{-1.66656pt}}\hspace{1.66656pt}}^{*(1)}=H^{(1)}\underline{\varvec{G\hspace{-1.66656pt}}\hspace{1.66656pt}}(\underline{\varvec{U\hspace{-1.66656pt}}\hspace{1.66656pt}}^{(1)})\in {\mathbb {R}}^{(3\times Q)}$. Then, we compute

28 $$\begin{aligned}  \underline{\varvec{U\hspace{-1.66656pt}}\hspace{1.66656pt}}^{(2)}&= \underline{\varvec{U\hspace{-1.66656pt}}\hspace{1.66656pt}}^{(0)}_3+\Delta t(\Theta ^{(2)} -\alpha \Gamma ^{(2)} )\underline{\varvec{G\hspace{-1.66656pt}}\hspace{1.66656pt}}^{*(1)} +\Delta t \alpha \Gamma ^{(2)} \underline{\varvec{G\hspace{-1.66656pt}}\hspace{1.66656pt}}(\underline{\varvec{U\hspace{-1.66656pt}}\hspace{1.66656pt}}^{(2)})\\&= \underline{\varvec{U\hspace{-1.66656pt}}\hspace{1.66656pt}}^{(0)}_3+\Delta t(\Theta ^{(2)} -\alpha \Gamma ^{(2)} )H^{(1)}\underline{\varvec{G\hspace{-1.66656pt}}\hspace{1.66656pt}}(\underline{\varvec{U\hspace{-1.66656pt}}\hspace{1.66656pt}}^{(1)})+\Delta t \alpha \Gamma ^{(2)} \underline{\varvec{G\hspace{-1.66656pt}}\hspace{1.66656pt}}(\underline{\varvec{U\hspace{-1.66656pt}}\hspace{1.66656pt}}^{(2)}),  \end{aligned}$$

which is in ${\mathbb {R}}^{(3\times Q)}$ and $O(\Delta t^3)$-accurate. We can iteratively continue with interpolations, $\underline{\varvec{G\hspace{-1.66656pt}}\hspace{1.66656pt}}^{*(p-1)}=H^{(p-1)}\underline{\varvec{G\hspace{-1.66656pt}}\hspace{1.66656pt}}(\underline{\varvec{U\hspace{-1.66656pt}}\hspace{1.66656pt}}^{(p-1)})$, and iterations, obtaining the general updating formula

29 $$\begin{aligned} \underline{\varvec{U\hspace{-1.66656pt}}\hspace{1.66656pt}}^{(p)} = \underline{\varvec{U\hspace{-1.66656pt}}\hspace{1.66656pt}}^{(0)}_{p+1}+\Delta t(\Theta ^{(p)} -\alpha \Gamma ^{(p)} )H^{(p-1)}\underline{\varvec{G\hspace{-1.66656pt}}\hspace{1.66656pt}}(\underline{\varvec{U\hspace{-1.66656pt}}\hspace{1.66656pt}}^{(p-1)})+\Delta t \alpha \Gamma ^{(p)} \underline{\varvec{G\hspace{-1.66656pt}}\hspace{1.66656pt}}(\underline{\varvec{U\hspace{-1.66656pt}}\hspace{1.66656pt}}^{(p)}) \end{aligned}$$

with $\underline{\varvec{U\hspace{-1.66656pt}}\hspace{1.66656pt}}^{(p)}\in {\mathbb {R}}^{((p+1)\times Q)}$ and $O(\Delta t^{p+1})$-accurate. Analogous considerations, as for the $\alpha$DeCu method, hold on the advantage of performing a final iteration with no interpolation when the final number of subtimenodes is fixed. Also in this case, the reader is referred to Fig. 2 for a better understanding of the method.

### $\alpha$DeCu and $\alpha$DeCdu with Gauss-Lobatto Subtimenodes

As already explained, $M+1$ GL subtimenodes can guarantee an accuracy equal to 2*M*. In such a case, if the final number of subtimenodes is fixed, we start with two subtimenodes and we alternate iterations of the $\alpha$DeC method and interpolations as in the equispaced case, adding one subtimenode at each iteration until reaching the desired $M+1$ subtimenodes, then, we continue with normal iterations of the $\alpha$DeC until $P=2M$ to get the maximal order of accuracy associated to such a choice. The updating formulas are identical to the ones already presented. The interpolation is not performed at the first iteration and from the $(M+1)$th iteration on. On the other hand, if the order *P* is fixed, the most efficient choice is given by a final number of subtimenodes equal to $M+1$ with $M=\left\lceil {\frac{P}{2}} \right\rceil$ and *P* iterations.

Contrary to what one might think, it is not possible to postpone an interpolation process after the saturation of the maximal accuracy associated to some intermediate number of GL subtimenodes adopted in the early iterations. The interpolation processes must mandatorily take place in the first iterations. This is due to the mismatch between the $O(\Delta t^{2p+1})$-accuracy of the operator ${\mathcal {L}}_{\Delta }^2$ associated to $p+1$ GL subtimenodes and the $O(\Delta t^{p+1})$-accuracy of the interpolation process with the same number of subtimenodes.

## The DeC as RK

An explicit RK method with *S* stages applied in the interval $[t_n,t_{n+1}]$ reads

30 $$\begin{aligned} {\left\{ \begin{array}{ll} \varvec{y\hspace{-1.66656pt}}\hspace{1.66656pt}^0=\varvec{u\hspace{-1.66656pt}}\hspace{1.66656pt}_n,\\ \varvec{y\hspace{-1.66656pt}}\hspace{1.66656pt}^s = \varvec{u\hspace{-1.66656pt}}\hspace{1.66656pt}_n + \Delta t\sum _{r = 0}^{s-1} a_{s,r} {\varvec{G\hspace{-1.66656pt}}\hspace{1.66656pt}}(t_n+c_r \Delta t, \varvec{y\hspace{-1.66656pt}}\hspace{1.66656pt}^r) \quad \text {for }s=1,\cdots ,S-1,\\ \varvec{u\hspace{-1.66656pt}}\hspace{1.66656pt}_{n+1} = \varvec{u\hspace{-1.66656pt}}\hspace{1.66656pt}_n + \Delta t\sum _{r = 0}^{S-1} b_{r} {\varvec{G\hspace{-1.66656pt}}\hspace{1.66656pt}}(t_n+c_r \Delta t, \varvec{y\hspace{-1.66656pt}}\hspace{1.66656pt}^r). \end{array}\right. } \end{aligned}$$

The coefficients $a_{sr}$, $c_r$, and $b_r$ uniquely characterize the RK method and can be stored, respectively, into the strictly lower triangular matrix *A* and the vectors $\varvec{c\hspace{-1.66656pt}}\hspace{1.66656pt}$ and $\varvec{b\hspace{-1.66656pt}}\hspace{1.66656pt}$, often summarized in a Butcher tableau

$$\begin{aligned} \begin{array}{c|c} \varvec{c\hspace{-1.66656pt}}\hspace{1.66656pt}&{} A \\ \hline &{} \varvec{b\hspace{-1.66656pt}}\hspace{1.66656pt}\end{array}. \end{aligned}$$

It is well known, as presented in [18, 21, 24], that DeC methods can be written into the RK form. This also holds for the new methods, $\alpha$DeCu and $\alpha$DeCdu. In this section, we will explicitly construct their Butcher tableaux. We will adopt a zero-based numeration and the following convention for slicing. If ${\mathcal {M}}\in {\mathbb {R}}^{D_0\times D_1}$, we denote by ${\mathcal {M}}_{i:j,k:\ell }$ its slice from the *i*th row to the *j*th row (included) and from the *k*th column to the $\ell$th column (included). We omit the last (first) index in case we want to include all the entries until the end (from the beginning), e.g., ${\mathcal {M}}_{1:,:6}=M_{1:D_0-1,0:6}$. The same notation is assumed for vectors. We define also the vectors ${\underline{\beta }}^{(p)}:=\left( 0,\frac{t^{1,(p)}-t_n}{\Delta t}, \cdots , \frac{t^{p,(p)}-t_n}{\Delta t} \right) ^{\rm{T}}$ of the $\beta ^m$ coefficients in different iterations of the new methods and, for the original $\alpha$DeC method, the fixed vector ${\underline{\beta }}:=\left( 0,\frac{t^{1}-t_n}{\Delta t}, \cdots , \frac{t^{M}-t_n}{\Delta t} \right) ^{\rm{T}}$. In order to make the Butcher tableaux as compact as possible, the computation of the solution in the different subtimenodes at the first iteration will be always made through the explicit Euler method. This little modification has no impact on the formal accuracy, since the first iteration is meant to provide a first order approximation of the solution.

We will focus on equispaced subtimenodes. The extension to the GL case is trivial: it suffices to repeat the block without interpolation, related to the final iteration of the standard method, for the needed number of times, $M-1$ in the optimal case.

### $\alpha$DeC

We recall the general updating formula of the $\alpha$DeC methods in a matricial form

31 $$\begin{aligned} \underline{\varvec{U\hspace{-1.66656pt}}\hspace{1.66656pt}}^{(p)} = \underline{\varvec{U\hspace{-1.66656pt}}\hspace{1.66656pt}}^{(0)}+\Delta t(\Theta -\alpha \Gamma )\underline{\varvec{G\hspace{-1.66656pt}}\hspace{1.66656pt}}(\underline{\varvec{U\hspace{-1.66656pt}}\hspace{1.66656pt}}^{(p-1)})+\Delta t \alpha \Gamma \underline{\varvec{G\hspace{-1.66656pt}}\hspace{1.66656pt}}(\underline{\varvec{U\hspace{-1.66656pt}}\hspace{1.66656pt}}^{(p)}). \end{aligned}$$

If we align each iteration one after the other and we consider the approximation in each subtimenode of each iteration as an RK stage, we can pass to the RK formulation. Indeed, we do not repeat the redundant states, i.e., all the $\varvec{u\hspace{-1.66656pt}}\hspace{1.66656pt}^{0,(p)}=\varvec{u\hspace{-1.66656pt}}\hspace{1.66656pt}_n$, and we keep only $\varvec{u\hspace{-1.66656pt}}\hspace{1.66656pt}^0$ as representative of all of them. This leads to the RK formulation (30) with the Butcher tableau as in Table 1, where we added on top and on the right side the references to the different iteration steps. The number of stages of this formulation amounts to $S=M P$ for any type of subtimenodes. If $\alpha =0$, the $\alpha$DeC method reduces to the bDeC method and the Butcher tableau simplifies to Table 2. In such case, we observe that we do not need the whole vector $\underline{\varvec{u\hspace{-1.66656pt}}\hspace{1.66656pt}}^{(P)}$, but we can just compute the component associated to the final subtimenode with the only $\underline{\varvec{u\hspace{-1.66656pt}}\hspace{1.66656pt}}^{(P-1)}$, leading to a total number of RK stages equal to $S=M(P-1)+1$.

**Table 1:** RK structures for the original $\alpha$DeC with equispaced subtimenodes, $\varvec{c\hspace{-1.66656pt}}\hspace{1.66656pt}$ at the left $\varvec{b\hspace{-1.66656pt}}\hspace{1.66656pt}$ at the bottom, *A* in the middle

$\varvec{c\hspace{-1.66656pt}}\hspace{1.66656pt}$	$\varvec{u\hspace{-1.66656pt}}\hspace{1.66656pt}^0$	$\underline{\varvec{u\hspace{-1.66656pt}}\hspace{1.66656pt}}^{(1)}$	$\underline{\varvec{u\hspace{-1.66656pt}}\hspace{1.66656pt}}^{(2)}$	$\underline{\varvec{u\hspace{-1.66656pt}}\hspace{1.66656pt}}^{(3)}$	$\cdots$	$\underline{\varvec{u\hspace{-1.66656pt}}\hspace{1.66656pt}}^{(M)}$	$\underline{\varvec{u\hspace{-1.66656pt}}\hspace{1.66656pt}}^{(M+1)}_{:M-1}$	*A*
0	0							$\varvec{u\hspace{-1.66656pt}}\hspace{1.66656pt}^0$
${\underline{\beta }}_{1:}$	${\underline{\beta }}_{1:}$	$\underline{{\underline{0}}}$						$\underline{\varvec{u\hspace{-1.66656pt}}\hspace{1.66656pt}}^{(1)}$
${\underline{\beta }}_{1:}$	$\Theta _{1:,0}$	$(\Theta -\alpha \Gamma )_{1:,1:}\!\!\!\!\!\!$	$\alpha \Gamma _{1:,1:}$	$\underline{{\underline{0}}}$				$\underline{\varvec{u\hspace{-1.66656pt}}\hspace{1.66656pt}}^{(2)}$
${\underline{\beta }}_{1:}$	$\Theta _{1:,0}$	$\underline{{\underline{0}}}$	$(\Theta -\alpha \Gamma )_{1:,1:}\!\!\!\!$	$\alpha \Gamma _{1:,1:}$	$\underline{{\underline{0}}}$			$\underline{\varvec{u\hspace{-1.66656pt}}\hspace{1.66656pt}}^{(3)}$
	$\vdots$	$\vdots$		$\ddots$	$\ddots$			$\vdots$
	$\vdots$	$\vdots$			$\ddots$	$\ddots$		$\vdots$
${\underline{\beta }}_{1:M-1}$	$\Theta _{1:M-1,0}\!\!\!\!\!\!\!$	$\underline{{\underline{0}}}$	$\cdots$	$\cdots$	$\underline{{\underline{0}}}$	$(\Theta -\alpha \Gamma )_{1:M-1,1:}\!\!\!\!$	$\alpha \Gamma _{1:M-1,1:M-1}$	$\underline{\varvec{u\hspace{-1.66656pt}}\hspace{1.66656pt}}^{(M+1)}_{:M-1}$
$\varvec{b\hspace{-1.66656pt}}\hspace{1.66656pt}$	$\Theta _{M,0}$	${\underline{0}}$	$\cdots$	$\cdots$	${\underline{0}}$	$(\Theta -\alpha \Gamma )_{M,1:}$	$\alpha \Gamma _{M,1:M-1}$	$\underline{\varvec{u\hspace{-1.66656pt}}\hspace{1.66656pt}}^{M,(M+1)}$

**Table 2:** RK structures for the original bDeC with equispaced subtimenodes, $\varvec{c\hspace{-1.66656pt}}\hspace{1.66656pt}$ at the left $\varvec{b\hspace{-1.66656pt}}\hspace{1.66656pt}$ at the bottom, *A* in the middle

$\varvec{c\hspace{-1.66656pt}}\hspace{1.66656pt}$	$\varvec{u\hspace{-1.66656pt}}\hspace{1.66656pt}^0$	$\underline{\varvec{u\hspace{-1.66656pt}}\hspace{1.66656pt}}^{(1)}$	$\underline{\varvec{u\hspace{-1.66656pt}}\hspace{1.66656pt}}^{(2)}$	$\underline{\varvec{u\hspace{-1.66656pt}}\hspace{1.66656pt}}^{(3)}$	$\cdots$	$\underline{\varvec{u\hspace{-1.66656pt}}\hspace{1.66656pt}}^{(M-1)}$	$\underline{\varvec{u\hspace{-1.66656pt}}\hspace{1.66656pt}}^{(M)}$	*A*
0	0							$\varvec{u\hspace{-1.66656pt}}\hspace{1.66656pt}^0$
${\underline{\beta }}_{1:}$	${\underline{\beta }}_{1:}$	$\underline{{\underline{0}}}$						$\underline{\varvec{u\hspace{-1.66656pt}}\hspace{1.66656pt}}^{(1)}$
${\underline{\beta }}_{1:}$	$\Theta _{1:,0}$	$\Theta _{1:,1:}$	$\underline{{\underline{0}}}$					$\underline{\varvec{u\hspace{-1.66656pt}}\hspace{1.66656pt}}^{(2)}$
${\underline{\beta }}_{1:}$	$\Theta _{1:,0}$	$\underline{{\underline{0}}}$	$\Theta _{1:,1:}$	$\underline{{\underline{0}}}$				$\underline{\varvec{u\hspace{-1.66656pt}}\hspace{1.66656pt}}^{(3)}$
	$\vdots$	$\vdots$		$\ddots$	$\ddots$			$\vdots$
	$\vdots$	$\vdots$			$\ddots$	$\ddots$		$\vdots$
${\underline{\beta }}_{1:}$	$\Theta _{1:,0}$	$\underline{{\underline{0}}}$	$\cdots$	$\cdots$	$\underline{{\underline{0}}}$	$\Theta _{1:,1:}$	$\underline{{\underline{0}}}$	$\underline{\varvec{u\hspace{-1.66656pt}}\hspace{1.66656pt}}^{(M)}$
$\varvec{b\hspace{-1.66656pt}}\hspace{1.66656pt}$	$\Theta _{M,0}$	${\underline{0}}$	$\cdots$	$\cdots$	$\cdots$	${\underline{0}}$	$\Theta _{M,1:}$	$\underline{\varvec{u\hspace{-1.66656pt}}\hspace{1.66656pt}}^{M,(M+1)}$

### bDeCu

Let us recall the general updating formulas of the $\alpha$DeCu methods

32 $$\begin{aligned} \underline{\varvec{U\hspace{-1.66656pt}}\hspace{1.66656pt}}^{*(p-1)} &=\, \underline{\varvec{U\hspace{-1.66656pt}}\hspace{1.66656pt}}^{(0)}_{p+1}+\Delta t H^{(p-1)}(\Theta ^{(p-1)} -\alpha \Gamma ^{(p-1)} )\underline{\varvec{G\hspace{-1.66656pt}}\hspace{1.66656pt}}(\underline{\varvec{U\hspace{-1.66656pt}}\hspace{1.66656pt}}^{*(p-2)})\\&\quad+\Delta t \alpha H^{(p-1)}\Gamma ^{(p-1)} \underline{\varvec{G\hspace{-1.66656pt}}\hspace{1.66656pt}}(\underline{\varvec{U\hspace{-1.66656pt}}\hspace{1.66656pt}}^{(p-1)}), \end{aligned}$$

33 $$\begin{aligned}&\underline{\varvec{U\hspace{-1.66656pt}}\hspace{1.66656pt}}^{(p)} = \underline{\varvec{U\hspace{-1.66656pt}}\hspace{1.66656pt}}^{(0)}_{p+1}+\Delta t (\Theta ^{(p)} -\alpha \Gamma ^{(p)} )\underline{\varvec{G\hspace{-1.66656pt}}\hspace{1.66656pt}}(\underline{\varvec{U\hspace{-1.66656pt}}\hspace{1.66656pt}}^{*(p-1)})+\Delta t \alpha \Gamma ^{(p)} \underline{\varvec{G\hspace{-1.66656pt}}\hspace{1.66656pt}}(\underline{\varvec{U\hspace{-1.66656pt}}\hspace{1.66656pt}}^{(p)}), \end{aligned}$$

to which we need to add an initial iteration made with Euler and either a final iteration or, in the context of GL subtimenodes, some final iterations (*M* in the optimal case) of the standard $\alpha$DeC method performed without interpolation. In this case, the stages of the RK method are given by all the components of the vectors $\underline{\varvec{U\hspace{-1.66656pt}}\hspace{1.66656pt}}^{(p)}$ and $\underline{\varvec{U\hspace{-1.66656pt}}\hspace{1.66656pt}}^{*(p)}$ (excluding the redundant states). From easy computations, one can see that for $\alpha \ne 0$ the number of stages of the $\alpha$DeCu method coincides with the number of stages of the $\alpha$DeC method without computational advantage under this point of view. For this reason, we focus on the bDeCu method ($\alpha = 0$), for which we have a substantial computational advantage. In such a case, the updating formulas (32) and (33) reduce to

34 $$\begin{aligned}&\underline{\varvec{U\hspace{-1.66656pt}}\hspace{1.66656pt}}^{*(p-1)} = \underline{\varvec{U\hspace{-1.66656pt}}\hspace{1.66656pt}}^{(0)}_{p+1}+\Delta t H^{(p-1)}\Theta ^{(p-1)} \underline{\varvec{G\hspace{-1.66656pt}}\hspace{1.66656pt}}(\underline{\varvec{U\hspace{-1.66656pt}}\hspace{1.66656pt}}^{*(p-2)}), \end{aligned}$$

35 $$\begin{aligned}&\underline{\varvec{U\hspace{-1.66656pt}}\hspace{1.66656pt}}^{(p)} = \underline{\varvec{U\hspace{-1.66656pt}}\hspace{1.66656pt}}^{(0)}_{p+1}+\Delta t \Theta ^{(p)} \underline{\varvec{G\hspace{-1.66656pt}}\hspace{1.66656pt}}(\underline{\varvec{U\hspace{-1.66656pt}}\hspace{1.66656pt}}^{*(p-1)}). \end{aligned}$$

The right-hand sides of the previous equations involve the computation of $\varvec{G\hspace{-1.66656pt}}\hspace{1.66656pt}$ in interpolated states $\underline{\varvec{U\hspace{-1.66656pt}}\hspace{1.66656pt}}^*$ only and, in particular, the update of $\underline{\varvec{U\hspace{-1.66656pt}}\hspace{1.66656pt}}^{*(p-1)}$ only depends on $\underline{\varvec{U\hspace{-1.66656pt}}\hspace{1.66656pt}}^{*(p-2)}$. This means that the scheme can be rewritten in terms of the vectors $\underline{\varvec{U\hspace{-1.66656pt}}\hspace{1.66656pt}}^{*(p)}$ only (plus $\underline{\varvec{U\hspace{-1.66656pt}}\hspace{1.66656pt}}^{M,(P)}$), drastically reducing the number of stages. The RK coefficients are reported in Table 3, in which we have

36 $$\begin{aligned} W^{(p)} : = {\left\{ \begin{array}{ll} H^{(p)}\Theta ^{(p)} \in {\mathbb {R}}^{(p+2)\times (p+1)}, &{}\text {if } p =2, \cdots , M-1,\\ \Theta ^{(M)} \in {\mathbb {R}}^{(M+1)\times (M+1)}, &{} \text {if } p \geqslant M. \end{array}\right. } \end{aligned}$$

The total number of RK stages is given by $S=M (P-1) +1 -\frac{(M-1)(M-2)}{2}$, so $\frac{(M-1)(M-2)}{2}$ less with respect to the original method. The formula holds for both equispaced and GL subtimenodes.

**Table 3:** RK structures for the bDeCu method, $\varvec{c\hspace{-1.66656pt}}\hspace{1.66656pt}$ at the left $\varvec{b\hspace{-1.66656pt}}\hspace{1.66656pt}$ at the bottom, *A* in the middle

$\varvec{c\hspace{-1.66656pt}}\hspace{1.66656pt}$	$\varvec{u\hspace{-1.66656pt}}\hspace{1.66656pt}^0$	$\underline{\varvec{u\hspace{-1.66656pt}}\hspace{1.66656pt}}^{*(1)}\!\!$	$\underline{\varvec{u\hspace{-1.66656pt}}\hspace{1.66656pt}}^{*(2)}\!\!$	$\underline{\varvec{u\hspace{-1.66656pt}}\hspace{1.66656pt}}^{*(3)}\!\!$	$\cdots \!\!$	$\underline{\varvec{u\hspace{-1.66656pt}}\hspace{1.66656pt}}^{*(M-2)}\!\!$	$\underline{\varvec{u\hspace{-1.66656pt}}\hspace{1.66656pt}}^{*(M-1)}\!\!$	$\underline{\varvec{u\hspace{-1.66656pt}}\hspace{1.66656pt}}^{(M)}\!\!$	*A*	dim
0	0								$\varvec{u\hspace{-1.66656pt}}\hspace{1.66656pt}^0$	1
${\underline{\beta }}^{(2)}_{1:}$	${\underline{\beta }}^{(2)}_{1:}$	$\underline{{\underline{0}}}$							$\underline{\varvec{u\hspace{-1.66656pt}}\hspace{1.66656pt}}^{*(1)}$	2
${\underline{\beta }}^{(3)}_{1:}$	$W^{(2)}_{1:,0}\!\!$	$W^{(2)}_{1:,1:}\!\!\!\!$	$\underline{{\underline{0}}}$						$\underline{\varvec{u\hspace{-1.66656pt}}\hspace{1.66656pt}}^{*(2)}$	3
${\underline{\beta }}^{(4)}_{1:}$	$W^{(3)}_{1:,0}$	$\underline{{\underline{0}}}$	$W^{(3)}_{1:,1:}\!\!\!\!$	$\underline{{\underline{0}}}$					$\underline{\varvec{u\hspace{-1.66656pt}}\hspace{1.66656pt}}^{*(3)}$	4
	$\vdots$	$\vdots$		$\ddots$	$\ddots$				$\vdots$	$\vdots$
	$\vdots$	$\vdots$			$\ddots$	$\ddots$			$\vdots$	$\vdots$
${\underline{\beta }}^{(M)}_{1:}\!\!$	$W^{(M-1)}_{1:,0}\!\!\!\!$	$\underline{{\underline{0}}}$	$\cdots$	$\cdots$	$\underline{{\underline{0}}}$	$W^{(M-1)}_{1:,1:}\!\!\!\!$	$\underline{{\underline{0}}}$	$\underline{{\underline{0}}}$	$\underline{\varvec{u\hspace{-1.66656pt}}\hspace{1.66656pt}}^{*(M-1)}\!\!$	*M*
${\underline{\beta }}^{(M)}_{1:}\!\!$	$W^{(M)}_{1:,0}$	$\underline{{\underline{0}}}$	$\cdots$	$\cdots$	$\cdots$	$\underline{{\underline{0}}}$	$W^{(M)}_{1:,1:}\!\!\!\!$	$\underline{{\underline{0}}}$	$\underline{\varvec{u\hspace{-1.66656pt}}\hspace{1.66656pt}}^{(M)}$	*M*
$\varvec{b\hspace{-1.66656pt}}\hspace{1.66656pt}$	$W^{(M+1)}_{M,0}$	${\underline{0}}$	$\cdots$	$\cdots$	$\cdots$	$\cdots$	${\underline{0}}$	$W^{(M+1)}_{M,1:}\!\!$	$\underline{\varvec{u\hspace{-1.66656pt}}\hspace{1.66656pt}}^{M,(M+1)}\!\!$	

#### Remark 1

(On the relation between stages and computational cost) The number of stages is not completely explanatory of the computational costs of the new algorithms. In the context of the novel methods, the cost associated to the computation of the different stages is not homogeneous, especially in applications to PDEs, as some of them are “properly” computed through the updating formula (16) of the original scheme, while the others are got through an interpolation process which is much cheaper. As an example, (32) can be computed as $\underline{\varvec{U\hspace{-1.66656pt}}\hspace{1.66656pt}}^{*(p-1)}=H^{(p-1)} \underline{\varvec{U\hspace{-1.66656pt}}\hspace{1.66656pt}}^{(p-2)}$. In particular, as already specified, the novel $\alpha$DeCu methods for $\alpha \ne 0$ are characterized by the same number of stages as the original $\alpha$DeC, nevertheless, roughly half of them is computed through interpolation. For this reason, they have been numerically investigated for $\alpha =1$.

### $\alpha$DeCdu

Again, we start by recalling the updating formulas of the method

37 $$\begin{aligned} \underline{\varvec{U\hspace{-1.66656pt}}\hspace{1.66656pt}}^{(p)} = \underline{\varvec{U\hspace{-1.66656pt}}\hspace{1.66656pt}}^{(0)}_{p+1}+\Delta t(\Theta ^{(p)} -\alpha \Gamma ^{(p)} )H^{(p-1)}\underline{\varvec{G\hspace{-1.66656pt}}\hspace{1.66656pt}}(\underline{\varvec{U\hspace{-1.66656pt}}\hspace{1.66656pt}}^{(p-1)})+\Delta t \alpha \Gamma ^{(p)} \underline{\varvec{G\hspace{-1.66656pt}}\hspace{1.66656pt}}(\underline{\varvec{U\hspace{-1.66656pt}}\hspace{1.66656pt}}^{(p)}), \end{aligned}$$

supplemented with an initial Euler step and a final iteration or, for GL subtimenodes, at most *M* final iterations of $\alpha$DeC without interpolation. The usual identification of subtimenodes and RK stages leads to the Butcher tableau in Table 4, in which we have

38 $$\begin{aligned} X^{(p)} : = {\left\{ \begin{array}{ll} (\Theta ^{(p)} - \alpha \Gamma ^{(p)} ) H^{(p-1)} \in {\mathbb {R}}^{(p+1)\times p}, &{}\text {if } p =2, \cdots , M,\\ \Theta ^{(M)} - \alpha \Gamma ^{(M)} \in {\mathbb {R}}^{(M+1)\times (M+1)}, &{} \text {if } p >M, \end{array}\right. } \end{aligned}$$

39 $$\begin{aligned} Y^{(p)} : = {\left\{ \begin{array}{ll} \alpha \Gamma ^{(p)} \in {\mathbb {R}}^{(p+1)\times (p+1)}, &{}\text {if } p =2, \cdots , M,\\ \alpha \Gamma ^{(M)} \in {\mathbb {R}}^{(M+1)\times (M+1)}, &{} \text {if } p >M. \end{array}\right. } \end{aligned}$$

The number of stages in this case amounts to $S=MP-\frac{M(M-1)}{2}$, with a computational advantage of $\frac{M(M-1)}{2}$ with respect to the original method.

**Table 4:** RK structures for the $\alpha$DeCdu method with equispaced subtimenodes, $\varvec{c\hspace{-1.66656pt}}\hspace{1.66656pt}$ at the left $\varvec{b\hspace{-1.66656pt}}\hspace{1.66656pt}$ at the bottom, *A* in the middle

$\varvec{c\hspace{-1.66656pt}}\hspace{1.66656pt}$	$\varvec{u\hspace{-1.66656pt}}\hspace{1.66656pt}^0$	$\underline{\varvec{u\hspace{-1.66656pt}}\hspace{1.66656pt}}^{(1)}$	$\underline{\varvec{u\hspace{-1.66656pt}}\hspace{1.66656pt}}^{(2)}$	$\underline{\varvec{u\hspace{-1.66656pt}}\hspace{1.66656pt}}^{(3)}$	$\cdots$	$\underline{\varvec{u\hspace{-1.66656pt}}\hspace{1.66656pt}}^{(M-2)}\!\!$	$\underline{\varvec{u\hspace{-1.66656pt}}\hspace{1.66656pt}}^{(M-1)}\!\!$	$\underline{\varvec{u\hspace{-1.66656pt}}\hspace{1.66656pt}}^{(M)}$	$\underline{\varvec{u\hspace{-1.66656pt}}\hspace{1.66656pt}}^{(M+1)}_{:M-1}$	*A*	dim
0	0									$\varvec{u\hspace{-1.66656pt}}\hspace{1.66656pt}^0$	1
${\underline{\beta }}^{(1)}_{1:}$	${\underline{\beta }}^{(1)}_{1:}$	$\underline{{\underline{0}}}$								$\underline{\varvec{u\hspace{-1.66656pt}}\hspace{1.66656pt}}^{(1)}$	1
${\underline{\beta }}^{(2)}_{1:}$	$X^{(2)}_{1:,0}$	$X^{(2)}_{1:,1:}\!\!$	$Y^{(2)}_{1:,1:}\!\!$							$\underline{\varvec{u\hspace{-1.66656pt}}\hspace{1.66656pt}}^{(2)}$	2
${\underline{\beta }}^{(3)}_{1:}$	$X^{(3)}_{1:,0}\!\!$	$\underline{{\underline{0}}}$	$X^{(3)}_{1:,1:}\!\!$	$Y^{(3)}_{1:,1:}\!\!$						$\underline{\varvec{u\hspace{-1.66656pt}}\hspace{1.66656pt}}^{(3)}$	3
	$\vdots$	$\vdots$		$\ddots$	$\ddots$					$\vdots$	$\vdots$
	$\vdots$	$\vdots$			$\ddots$	$\ddots$				$\vdots$	$\vdots$
${\underline{\beta }}^{(M-1)}_{1:}\!\!$	$X^{(M-1)}_{1:,0}\!\!\!\!$	$\underline{{\underline{0}}}$	$\cdots$	$\cdots$	$\underline{{\underline{0}}}$	$X^{(M-1)}_{1:,1:}\!\!\!$	$Y^{(M-1)}_{1:,1:}\!\!\!\!$	$\underline{{\underline{0}}}$		$\underline{\varvec{u\hspace{-1.66656pt}}\hspace{1.66656pt}}^{(M-1)}\!\!\!\!$	$M-1\!\!$
${\underline{\beta }}^{(M)}_{1:}\!\!$	$X^{(M)}_{1:,0}\!\!$	$\underline{{\underline{0}}}$	$\cdots$	$\cdots$	$\cdots$	$\underline{{\underline{0}}}$	$X^{(M)}_{1:,1:}\!\!$	$Y^{(M)}_{1:,1:}\!\!$		$\underline{\varvec{u\hspace{-1.66656pt}}\hspace{1.66656pt}}^{(M)}\!\!$	*M*
${\underline{\beta }}^{(M)}_{1:M-1}\!\!$	$X^{(M+1)}_{1:M-1,0}\!\!\!\!$	$\underline{{\underline{0}}}$	$\cdots$	$\cdots$	$\cdots$	$\cdots$	$\underline{{\underline{0}}}$	$X^{(M+1)}_{1:M-1,1:}\!\!\!\!$	$Y^{(M+1)}_{1:M-1,1:M-1}\!$	$\underline{\varvec{u\hspace{-1.66656pt}}\hspace{1.66656pt}}^{(M+1)}_{1:M-1}\!\!\!\!$	$M-1\!\!$
$\varvec{b\hspace{-1.66656pt}}\hspace{1.66656pt}$	$X^{(M+1)}_{M,0}\!\!\!$	${\underline{0}}$	$\cdots$	$\cdots$	$\cdots$	$\cdots$	${\underline{0}}$	$X^{(M+1)}_{M,1:}\!\!$	$Y^{(M+1)}_{M,1:M-1}\!\!$	$\underline{\varvec{u\hspace{-1.66656pt}}\hspace{1.66656pt}}^{M,(M+1)}\!\!$	

Also in this case, it is worth giving a particular attention to the method given by $\alpha =0$. Again, the possibility to compute $\underline{\varvec{u\hspace{-1.66656pt}}\hspace{1.66656pt}}^{M,(P)}$ without any need for the other components of $\underline{\varvec{u\hspace{-1.66656pt}}\hspace{1.66656pt}}^{(P)}$ further reduces the number of stages to $S=M(P-1) +1 -\frac{M(M-1)}{2}$.

We conclude this section with two tables, Tables 5 and 6, containing the number of stages of the original methods and of the novel ones, respectively for equispaced and GL subtimenodes, up to order 13 with associated theoretical speed up factors computed as the ratios between the stages of the original methods and the stages of the modified methods.

**Table 5:** Number of stages for the original ($\alpha$DeC, bDeC) and novel ($\alpha$DeCu, $\alpha$DeCdu, bDeCu, bDeCdu) methods with equispaced subtimenodes and speed up factor

		$\alpha$DeC			bDeC				
		RK stages		Speed up	RK stages			Speed up	
*P*	*M*	$\alpha$DeC/$\alpha$DeCu	$\alpha$DeCdu	$\alpha$DeCdu	bDeC	bDeCu	bDeCdu	bDeCu	bDeCdu
2	1	2	2	1.000	2	2	2	1.000	1.000
3	2	6	5	1.200	5	5	4	1.000	1.250
4	3	12	9	1.333	10	9	7	1.111	1.429
5	4	20	14	1.429	17	14	11	1.214	1.545
6	5	30	20	1.500	26	20	16	1.300	1.625
7	6	42	27	1.556	37	27	22	1.370	1.682
8	7	56	35	1.600	50	35	29	1.429	1.724
9	8	72	44	1.636	65	44	37	1.477	1.757
10	9	90	54	1.667	82	54	46	1.519	1.783
11	10	110	65	1.692	101	65	56	1.554	1.804
12	11	132	77	1.714	122	77	67	1.584	1.821
13	12	156	90	1.733	145	90	79	1.611	1.835

**Table 6:** Number of stages for the original ($\alpha$DeC, bDeC) and novel ($\alpha$DeCu, $\alpha$DeCdu, bDeCu, bDeCdu) methods with GL subtimenodes and speed up factor

		$\alpha$DeC			bDeC				
		RK stages		Speed up	RK stages			Speed up	
*P*	*M*	$\alpha$DeC/$\alpha$DeCu	$\alpha$DeCdu	$\alpha$DeCdu	bDeC	bDeCu	bDeCdu	bDeCu	bDeCdu
2	1	2	2	1.000	2	2	2	1.000	1.000
3	2	6	5	1.200	5	5	4	1.000	1.250
4	2	8	7	1.143	7	7	6	1.000	1.167
5	3	15	12	1.250	13	12	10	1.083	1.300
6	3	18	15	1.200	16	15	13	1.067	1.231
7	4	28	22	1.273	25	22	19	1.136	1.316
8	4	32	26	1.231	29	26	23	1.115	1.261
9	5	45	35	1.286	41	35	31	1.171	1.323
10	5	50	40	1.250	46	40	36	1.150	1.278
11	6	66	51	1.294	61	51	46	1.196	1.326
12	6	72	57	1.263	67	57	52	1.175	1.288
13	7	91	70	1.300	85	70	64	1.214	1.328

## Stability Analysis

In this section, we study the stability of the novel DeC schemes. We will prove two original results. First, the stability functions of bDeCu and bDeCdu coincide with the bDeC ones and do not depend on the distribution of the subtimenodes but only on the order. Second, if we fix the subtimenodes distribution and the order, the $\alpha$DeCdu methods coincide with the $\alpha$DeCu methods on linear problems. For all the schemes, we will show the stability region using some symbolical and numerical tools.

Let us start by reviewing some known results for RK methods [10, 37]. The linear stability of an RK scheme is tested on Dahlquist’s problem $u'=\lambda u,$ where $\lambda \in {\mathbb {C}}$ with ${\rm{Re}}(\lambda )<0$. Being the RK schemes linear, we can write a general RK iteration as $u_{n+1}=R(\lambda \Delta t) u_n$, with $R(\cdot )$ the stability function of the method. The stability function is defined as

40 $$\begin{aligned} R(z) = 1+ z\varvec{b\hspace{-1.66656pt}}\hspace{1.66656pt}^{\rm{T}} (I-zA)^{-1} {\textbf{1}}, \end{aligned}$$

where ${\textbf{1}}$ is a vector with all the entries equal to 1. The set of complex numbers *z* such that $|R(z)|< 1$ is called the stability region. We remark that the stability function for explicit RK methods is a polynomial. In fact, the inverse of $(I-zA)$ can be written in the Taylor expansion as

41 $$\begin{aligned} (I-zA)^{-1} = \sum _{r=0}^{\infty } z^rA^r = I + zA + z^2A^2+\cdots \end{aligned}$$

and, since *A* is strictly lower triangular, it is nilpotent, i.e., there exists an integer *r* such that $A^r=\underline{{\underline{0}}}$ and the minimum of these natural numbers ${\mathcal {N}}$ is called degree of nilpotence. By definition of ${\mathcal {N}}$, it is clear that $A^{{\mathcal {N}}+r}=\underline{{\underline{0}}}$ for all $r \geqslant 0$. Moreover, it is also clear that ${\mathcal {N}}\leqslant S$, where *S* is the number of stages of the explicit RK method and the dimension of the matrix *A*. Hence, *R*(*z*) is a polynomial in *z* with degree at most equal to *S*. We recall that [37], if an RK method is of order *P*, then

42 $$\begin{aligned} R(z) = 1 + z + \frac{z^2}{2!} +\cdots +\frac{z^P}{P!}+O(z^{P+1}). \end{aligned}$$

Thus, we know the first $P+1$ terms of the stability functions $R(\cdot )$ for all the DeCs of order *P* presented above. Further, the following result holds.

### Theorem 2

The stability function of any bDeC, bDeCu, and bDeCdu method of order *P* is

43 $$\begin{aligned} R(z) = \sum _{r=0}^{P} \frac{z^r}{r!}= 1 + z + \frac{z^2}{2!} +\cdots +\frac{z^P}{P!}, \end{aligned}$$

and does not depend on the distribution of the subtimenodes.

### Proof

The proof of this theorem relies only on the block structure of the matrix *A* for such schemes. In all these cases, the matrix *A* can be written as

44 $$\begin{aligned} A= \begin{pmatrix} 0 &{} 0 &{} 0&{} \dots &{}0 &{}0\\ \star &{} 0 &{} 0&{} \dots &{} 0 &{}0\\ \star &{} \star &{} 0 &{} \dots &{} 0 &{}0\\ \star &{} 0&{} \star &{} \dots &{} 0 &{}0\\ \dots &{} \dots &{} \dots &{}\dots &{}\dots &{}\dots \\ \star &{} 0 &{} 0 &{}\cdots &{}\star &{}0 \end{pmatrix}, \end{aligned}$$

where $\star$ are some non-zero block matrices and the 0 are some zero block matrices of different sizes. The number of blocks in each row and column of *A* is *P*, the order of the scheme. By induction, we can prove that $A^k$ has zeros in the main block diagonal, and in all the $k-1$ block diagonals below the main diagonal, i.e., $(A^k)_{i,j} = 0$ if $i< j+k$, where the indices here refer to the blocks. Indeed, it is true that $A_{i,j}=0$ if $i<j+1$. Now, let us consider the entry $(A^{k+1})_{i,j}$ with $i<j+k+1$, i.e., $i-k<j+1$. Such entry is defined as $(A^{k+1})_{i,j} = \sum _{w} (A^{k})_{i,w} A_{w,j},$ and we will prove that all the terms of the sum are 0. Let $w<j+1$. Then $A_{w,j}=0$ because of the structure of *A*; while, if $w \geqslant j+1 >i-k$, we have that $i<w+k$, so $(A^k)_{i,w} = 0$ by induction.

In particular, this means that $A^{P}=\underline{{\underline{0}}}$, because any block row index *i* is smaller than $j+P$ for any block column index *j*, as *P* is the number of the blocks that we have in each row and column. Hence,

45 $$\begin{aligned} (I-zA)^{-1}=\sum _{r=0}^{\infty } z^rA^r =\sum _{r=0}^{P-1} z^rA^r = I + zA + z^2A^2+\cdots + z^{P-1}A^{P-1}. \end{aligned}$$

Plugging this result into (40), we can state that the stability function *R*(*z*) is a polynomial of degree *P*, the order of the scheme. Since all the terms of degree lower or equal to *P* must agree with the expansion of the exponential function (42), the stability function must be (43). Finally, let us notice that no assumption has been made on the distribution of the subtimenodes, hence, the result is general for any distribution.

In the following, we will show that, given a certain order *P* and a distribution of subtimenodes, the $\alpha$DeCu and $\alpha$DeCdu methods are equivalent on linear problems and, as a consequence, they share the same stability functions.

### Theorem 3

(Equivalence on linear problems) Given an order *P*, a distribution of subtimenodes and $\alpha \in [0,1]$, the schemes $\alpha$DeCu and $\alpha$DeCdu applied to linear systems are equivalent.

### Proof

Without loss of generality, we can focus on Dahlquist’s equation $u'=\lambda u$. Since the schemes are linear, the same arguments would apply component-wise also on linear systems of equations. Let us start by explicitly writing down the general updating formula (29) of the $\alpha$DeCdu method for Dahlquist’s equation

46 $$\begin{aligned} \underline{\varvec{U\hspace{-1.66656pt}}\hspace{1.66656pt}}^{(p)} = \underline{\varvec{U\hspace{-1.66656pt}}\hspace{1.66656pt}}^{(0)}_{p+1}+\Delta t \lambda (\Theta ^{(p)} -\alpha \Gamma ^{(p)} )H^{(p-1)}\underline{\varvec{U\hspace{-1.66656pt}}\hspace{1.66656pt}}^{(p-1)}+\Delta t \lambda \alpha \Gamma ^{(p)}\underline{\varvec{U\hspace{-1.66656pt}}\hspace{1.66656pt}}^{(p)}. \end{aligned}$$

For the $\alpha$DeCu method, the updating formula (26) becomes

47 $$\begin{aligned} \underline{\varvec{U\hspace{-1.66656pt}}\hspace{1.66656pt}}^{(p)} = \underline{\varvec{U\hspace{-1.66656pt}}\hspace{1.66656pt}}^{(0)}_{p+1}+\Delta t \lambda (\Theta ^{(p)} -\alpha \Gamma ^{(p)} )\underline{\varvec{U\hspace{-1.66656pt}}\hspace{1.66656pt}}^{*(p-1)}+\Delta t \lambda \alpha \Gamma ^{(p)} \underline{\varvec{U\hspace{-1.66656pt}}\hspace{1.66656pt}}^{(p)}. \end{aligned}$$

Now, using the definition of $\underline{\varvec{U\hspace{-1.66656pt}}\hspace{1.66656pt}}^{*(p-1)} = H^{(p-1)}\underline{\varvec{U\hspace{-1.66656pt}}\hspace{1.66656pt}}^{(p-1)}$, we obtain

48 $$\begin{aligned} \underline{\varvec{U\hspace{-1.66656pt}}\hspace{1.66656pt}}^{(p)} = \underline{\varvec{U\hspace{-1.66656pt}}\hspace{1.66656pt}}^{(0)}_{p+1}+\Delta t \lambda (\Theta ^{(p)} -\alpha \Gamma ^{(p)} )H^{(p-1)}\underline{\varvec{U\hspace{-1.66656pt}}\hspace{1.66656pt}}^{(p-1)}+\Delta t \lambda \alpha \Gamma ^{(p)} \underline{\varvec{U\hspace{-1.66656pt}}\hspace{1.66656pt}}^{(p)}, \end{aligned}$$

which coincides with (46). This means that, at each iteration, the two modified schemes coincide.

**Fig. 3:**
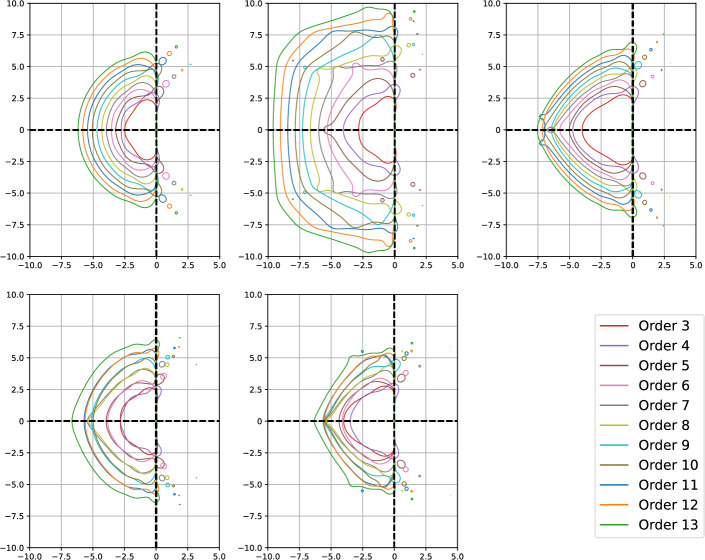
Stability regions for various schemes with order from 3 to 13: bDeC, bDeCu, and bDeCdu (equivalent) for any distribution of subtimenodes (top left), sDeC for equispaced subtimenodes (top center), sDeCu and sDeCdu (equivalent) for equispaced subtimenodes (top right), sDeC for GL subtimenodes (bottom left), sDeCu and sDeCdu (equivalent) (bottom center), legend (bottom right)

In Fig. 3, we depict the stability region of all the presented methods from order 3 to 13. We remark that there is no difference in terms of the stability between bDeC, bDeCu, and bDeCdu, nor dependence on the distribution of the subtimenodes, as well as sDeCu and sDeCdu have the same stability regions for fixed subtimenodes.

## Application to Hyperbolic PDEs

In this section, we apply the novel explicit efficient DeC techniques to hyperbolic PDEs. We will focus on the CG framework, which is particularly challenging with respect to FV and DG formulations, due to the presence of a global sparse mass matrix. In particular, we will consider two strategies that allow to avoid the related issues. We will describe the operators ${\mathcal {L}}_{\Delta }^1$ and ${\mathcal {L}}_{\Delta }^2$ for the two strategies in the bDeC formulation and see how to apply the bDeCu efficient modification. The proofs of the properties of the operators are provided in the supplementary material.

### Continuous Galerkin FEM

The general form of a hyperbolic system of balance laws reads

49 $$\begin{aligned} \frac{\partial }{\partial t}\varvec{u\hspace{-1.66656pt}}\hspace{1.66656pt}(\varvec{x\hspace{-1.66656pt}}\hspace{1.66656pt},t)+\text {div}_{\varvec{x\hspace{-1.66656pt}}\hspace{1.66656pt}} {\varvec{F}}(\varvec{u\hspace{-1.66656pt}}\hspace{1.66656pt}(\varvec{x\hspace{-1.66656pt}}\hspace{1.66656pt},t))={\varvec{S}}(\varvec{x\hspace{-1.66656pt}}\hspace{1.66656pt},\varvec{u\hspace{-1.66656pt}}\hspace{1.66656pt}(\varvec{x\hspace{-1.66656pt}}\hspace{1.66656pt},t)), \quad (\varvec{x\hspace{-1.66656pt}}\hspace{1.66656pt},t) \in \Omega \times {\mathbb {R}}^+_0, \end{aligned}$$

where $\varvec{u\hspace{-1.66656pt}}\hspace{1.66656pt}{:}\,\Omega \times {\mathbb {R}}^+_0\rightarrow {\mathbb {R}}^Q$, with some initial condition $\varvec{u\hspace{-1.66656pt}}\hspace{1.66656pt}(\varvec{x\hspace{-1.66656pt}}\hspace{1.66656pt},0)=\varvec{u\hspace{-1.66656pt}}\hspace{1.66656pt}_0(\varvec{x\hspace{-1.66656pt}}\hspace{1.66656pt})$ on the space domain $\Omega \subseteq {\mathbb {R}}^D$, and boundary conditions on $\partial \Omega$. We consider a tessellation ${\mathcal {T}}_h$ of ${\overline{\Omega }}$ with characteristic length *h*, made by convex closed polytopals *K*, and we introduce the space of continuous piecewise polynomial functions $V_h:=\lbrace g \in C^0({\overline{\Omega }})~ s.t.~g\vert _K \in {\mathbb {P}}_M(K),\,\forall K \in {\mathcal {T}}_h\rbrace$. We choose a basis $\lbrace \varphi _i \rbrace _{i=1,\cdots ,I}$ of $V_h$, e.g., the Lagrange polynomials or the Bernstein polynomials, which is such that each basis function $\varphi _i$ can be associated to a degree of freedom (DoF) $\varvec{x\hspace{-1.66656pt}}\hspace{1.66656pt}_i\in {\overline{\Omega }}$ and such that ${\rm{supp}}\left\{ \varphi _i\right\} =\cup _{K\in K_i}K$ with $K_i:=\lbrace K\in {\mathcal {T}}_h~ s.t. ~ \varvec{x\hspace{-1.66656pt}}\hspace{1.66656pt}_i\in K \rbrace$. Further, we assume a normalization of the basis functions yielding $\sum _{i=1}^I\varphi _i \equiv 1.$ Then, we project the weak formulation in space of the PDE (49) over $V_h$, i.e., we look for $\varvec{u\hspace{-1.66656pt}}\hspace{1.66656pt}_h(\varvec{x\hspace{-1.66656pt}}\hspace{1.66656pt},t)=\sum _{j=1}^I\varvec{c\hspace{-1.66656pt}}\hspace{1.66656pt}_j(t)\varphi _j(\varvec{x\hspace{-1.66656pt}}\hspace{1.66656pt}) \in V_h^Q$ such that for any $i=1,\cdots ,I$

50 $$\begin{aligned} \int _{\Omega } \left( \frac{\partial }{\partial t}\varvec{u\hspace{-1.66656pt}}\hspace{1.66656pt}_h(\varvec{x\hspace{-1.66656pt}}\hspace{1.66656pt},t)+\text {div}_{\varvec{x\hspace{-1.66656pt}}\hspace{1.66656pt}} {\varvec{F}}(\varvec{u\hspace{-1.66656pt}}\hspace{1.66656pt}_h(\varvec{x\hspace{-1.66656pt}}\hspace{1.66656pt},t))-{\varvec{S}}(\varvec{x\hspace{-1.66656pt}}\hspace{1.66656pt},\varvec{u\hspace{-1.66656pt}}\hspace{1.66656pt}_h(\varvec{x\hspace{-1.66656pt}}\hspace{1.66656pt},t))\right) \varphi _i(\varvec{x\hspace{-1.66656pt}}\hspace{1.66656pt}) \textrm{d}\varvec{x\hspace{-1.66656pt}}\hspace{1.66656pt}+\varvec{ST\hspace{-1.66656pt}}\hspace{1.66656pt}_i(\varvec{u\hspace{-1.66656pt}}\hspace{1.66656pt}_h)=\varvec{0\hspace{-1.66656pt}}\hspace{1.66656pt}, \end{aligned}$$

where the stabilization term $\varvec{ST\hspace{-1.66656pt}}\hspace{1.66656pt}_i(\varvec{u\hspace{-1.66656pt}}\hspace{1.66656pt}_h)$ is added to avoid the instabilities associated to central schemes. Thanks to the assumption on the support of the basis functions, it is possible to recast (50) as

51 $$\begin{aligned} \sum _{K\in K_i}\sum _{\varvec{x\hspace{-1.66656pt}}\hspace{1.66656pt}_j \in K} \left( \int _K \varphi _i(\varvec{x\hspace{-1.66656pt}}\hspace{1.66656pt}) \varphi _j(\varvec{x\hspace{-1.66656pt}}\hspace{1.66656pt}) \textrm{d} \varvec{x\hspace{-1.66656pt}}\hspace{1.66656pt}\right) \frac{\textrm{d}}{\textrm{d} t} \varvec{c\hspace{-1.66656pt}}\hspace{1.66656pt}_j(t)+ \varvec{\phi }_i(\varvec{c\hspace{-1.66656pt}}\hspace{1.66656pt}(t))=\varvec{0\hspace{-1.66656pt}}\hspace{1.66656pt}, \quad i=1,\cdots ,I, \end{aligned}$$

where $\varvec{c\hspace{-1.66656pt}}\hspace{1.66656pt}$ is the vector of all $\varvec{c\hspace{-1.66656pt}}\hspace{1.66656pt}_i$ and the space residuals $\varvec{\phi }_i(\varvec{c\hspace{-1.66656pt}}\hspace{1.66656pt}(t))$ are defined as

52 $$\begin{aligned} \varvec{\phi }_i(\varvec{c\hspace{-1.66656pt}}\hspace{1.66656pt}(t))\!=\!\!\sum _{K\in K_i} \!\int _{K}\!\! \left( \text {div}_{\varvec{x\hspace{-1.66656pt}}\hspace{1.66656pt}} 
{\varvec{F}}(\varvec{u\hspace{-1.66656pt}}\hspace{1.66656pt}_h(\varvec{x\hspace{-1.66656pt}}\hspace{1.66656pt},t))\!-\!{\varvec{S}}(\varvec{x\hspace{-1.66656pt}}\hspace{1.66656pt},\varvec{u\hspace{-1.66656pt}}\hspace{1.66656pt}_h(\varvec{x\hspace{-1.66656pt}}\hspace{1.66656pt},t))\right) \varphi _i(\varvec{x\hspace{-1.66656pt}}\hspace{1.66656pt}) \textrm{d}\varvec{x\hspace{-1.66656pt}}\hspace{1.66656pt}+\varvec{ST\hspace{-1.66656pt}}\hspace{1.66656pt}_i(\varvec{u\hspace{-1.66656pt}}\hspace{1.66656pt}_h). \end{aligned}$$

We would like to solve this system of ODEs in time without solving any linear system at each iteration nor inverting the huge mass matrix.

The first possibility consists in adopting particular basis functions, which, combined with the adoption of the induced quadrature formulas, allow to achieve a high order lumping of the mass matrix. This leads to a system of ODEs like the one described in the previous section and, hence, the novel methods can be applied in a straightforward way. Examples of such basis functions are given by the Lagrange polynomials associated to the GL points in one-dimensional (1D) domains and the Cubature elements in two-dimensional (2D) domains, introduced in [15] and studied in [20, 26, 27, 32]. The second strategy, introduced by Abgrall in [2] and based on the concept of residual [1, 4, 6, 33], exploits the abstract DeC formulation presented in Sect. 2, introducing a first order lumping in the mass matrix of the operator ${\mathcal {L}}_{\Delta }^1$, resulting in a fully explicit scheme, as we will explain in detail in the following.

### DeC for CG

In this section, we will define the operators ${\mathcal {L}}_{\Delta }^1$ and ${\mathcal {L}}_{\Delta }^2$ of the DeC formulation for CG FEM discretizations proposed by Abgrall in [2]. In this context, the parameter $\Delta$ of the DeC is the mesh parameter *h* of the space discretization. We assume CFL conditions of the type $\Delta \approx \Delta t \approx h$.

The definition of the high order implicit operator ${\mathcal {L}}_{\Delta }^2$ is not very different from the one seen in the context of the bDeC method for ODEs. We denote by $\varvec{c\hspace{-1.66656pt}}\hspace{1.66656pt}(t^m)$ the exact solution of the ODE (51) in the subtimenode $t^m$ and by $\varvec{c\hspace{-1.66656pt}}\hspace{1.66656pt}^m$ its approximation, containing, respectively, all components $\varvec{c\hspace{-1.66656pt}}\hspace{1.66656pt}_i(t^m)$ and $\varvec{c\hspace{-1.66656pt}}\hspace{1.66656pt}_i^m$. As usual, for the first subtimenode we set $\varvec{c\hspace{-1.66656pt}}\hspace{1.66656pt}^0=\varvec{c\hspace{-1.66656pt}}\hspace{1.66656pt}(t^0)=\varvec{c\hspace{-1.66656pt}}\hspace{1.66656pt}(t_n)=\varvec{c\hspace{-1.66656pt}}\hspace{1.66656pt}_n$. Starting from the exact integration of (51) over $[t^0,t^m]$ and replacing $\varvec{\phi }_i(\varvec{c\hspace{-1.66656pt}}\hspace{1.66656pt}(t))$ by its *M*th order interpolation in time associated to the $M+1$ subtimenodes, we get the definition of the operator ${\mathcal {L}}_{\Delta }^2{:}\,{\mathbb {R}}^{(I \times Q \times M)}\rightarrow {\mathbb {R}}^{(I \times Q \times M)}$ as

53 $$\begin{aligned} {\mathcal {L}}_{\Delta }^2(\underline{\varvec{c\hspace{-1.66656pt}}\hspace{1.66656pt}})=\left( {\mathcal {L}}^2_{\Delta ,1}(\underline{\varvec{c\hspace{-1.66656pt}}\hspace{1.66656pt}}),{\mathcal {L}}^2_{\Delta ,2}(\underline{\varvec{c\hspace{-1.66656pt}}\hspace{1.66656pt}}),\cdots ,{\mathcal {L}}^2_{\Delta ,I}(\underline{\varvec{c\hspace{-1.66656pt}}\hspace{1.66656pt}})\right) , \quad \forall \underline{\varvec{c\hspace{-1.66656pt}}\hspace{1.66656pt}}\in {\mathbb {R}}^{(I \times Q \times M)}, \end{aligned}$$

where, for any $i=1,\cdots ,I$ and $m=1,\cdots ,M$, we have

54 $$\begin{aligned} {\mathcal {L}}_{\Delta ,i}^{2,m}(\underline{\varvec{c\hspace{-1.66656pt}}\hspace{1.66656pt}})= \sum \limits _{K\in K_i}\sum \limits _{\varvec{x\hspace{-1.66656pt}}\hspace{1.66656pt}_j \in K} \left( \int _K \varphi _i(\varvec{x\hspace{-1.66656pt}}\hspace{1.66656pt}) \varphi _j(\varvec{x\hspace{-1.66656pt}}\hspace{1.66656pt}) \textrm{d} \varvec{x\hspace{-1.66656pt}}\hspace{1.66656pt}\right) \left( \varvec{c\hspace{-1.66656pt}}\hspace{1.66656pt}_j^m-\varvec{c\hspace{-1.66656pt}}\hspace{1.66656pt}_j^0\right) +\Delta t \sum \limits _{\ell =0}^{M} \theta ^m_\ell \varvec{\phi }_i(\varvec{c\hspace{-1.66656pt}}\hspace{1.66656pt}^\ell ). \end{aligned}$$

The solution $\underline{\varvec{c\hspace{-1.66656pt}}\hspace{1.66656pt}}_\Delta$ to ${\mathcal {L}}_{\Delta }^2(\underline{\varvec{c\hspace{-1.66656pt}}\hspace{1.66656pt}})=\varvec{0\hspace{-1.66656pt}}\hspace{1.66656pt}$ is $(M+1)$th order accurate. Unfortunately, such problem is a huge nonlinear system difficult to directly solve. According to the DeC philosophy, we introduce the operator ${\mathcal {L}}_{\Delta }^1$ making use of low order approximations of (51) to achieve an explicit formulation. In particular, we use the forward Euler time discretization and a first order mass lumping, obtaining ${\mathcal {L}}_{\Delta }^1{:}\,{\mathbb {R}}^{(I \times Q \times M)}\rightarrow {\mathbb {R}}^{(I \times Q \times M)}$

55 $$\begin{aligned} {\mathcal {L}}_{\Delta }^1(\underline{\varvec{c\hspace{-1.66656pt}}\hspace{1.66656pt}})=\left( {\mathcal {L}}^1_{\Delta ,1}(\underline{\varvec{c\hspace{-1.66656pt}}\hspace{1.66656pt}}),{\mathcal {L}}^1_{\Delta ,2}(\underline{\varvec{c\hspace{-1.66656pt}}\hspace{1.66656pt}}),\cdots ,{\mathcal {L}}^1_{\Delta ,I}(\underline{\varvec{c\hspace{-1.66656pt}}\hspace{1.66656pt}})\right) , \quad \forall \underline{\varvec{c\hspace{-1.66656pt}}\hspace{1.66656pt}}\in {\mathbb {R}}^{(I \times Q \times M)}, \end{aligned}$$

whose components, for any $i=1,\cdots ,I$ and $m=1,\cdots ,M$, are defined as

56 $$\begin{aligned} {\mathcal {L}}_{\Delta ,i}^{1,m}(\underline{\varvec{c\hspace{-1.66656pt}}\hspace{1.66656pt}}):= C_i \left( \varvec{c\hspace{-1.66656pt}}\hspace{1.66656pt}_i^m-\varvec{c\hspace{-1.66656pt}}\hspace{1.66656pt}_i^0\right) + \Delta t\beta ^{m}\varvec{\phi }_i(\varvec{c\hspace{-1.66656pt}}\hspace{1.66656pt}^0) \end{aligned}$$

with $$C_i:=\int _\Omega \varphi _i(\varvec{x\hspace{-1.66656pt}}\hspace{1.66656pt})\textrm{d} \varvec{x\hspace{-1.66656pt}}\hspace{1.66656pt}$$.

#### Remark 2

(Choice of the basis functions) For any *m* and *i*, we can explicitly compute $\varvec{c\hspace{-1.66656pt}}\hspace{1.66656pt}_i^m$ from ${\mathcal {L}}_{\Delta ,i}^{1,m}(\underline{\varvec{c\hspace{-1.66656pt}}\hspace{1.66656pt}})=\varvec{0\hspace{-1.66656pt}}\hspace{1.66656pt}$ if and only if $C_i\ne 0$. This means that the construction of the operator ${\mathcal {L}}_{\Delta }^1$ is not always well-posed for any arbitrary basis of polynomials. For example, with Lagrange polynomials of degree 2 on triangular meshes, we have $\int _\Omega \varphi _i(\varvec{x\hspace{-1.66656pt}}\hspace{1.66656pt}) \textrm{d}\varvec{x\hspace{-1.66656pt}}\hspace{1.66656pt}=0$ for some *i*. However, the construction is always well-posed with Bernstein bases, which verify $C_i>0$ for all *i*.

Let us characterize the iterative formula (3) in this context. We have

57 $$\begin{aligned} {\mathcal {L}}_\Delta ^1(\underline{\varvec{c\hspace{-1.66656pt}}\hspace{1.66656pt}}^{(p)})={\mathcal {L}}_\Delta ^1(\underline{\varvec{c\hspace{-1.66656pt}}\hspace{1.66656pt}}^{(p-1)})-{\mathcal {L}}_\Delta ^2(\underline{\varvec{c\hspace{-1.66656pt}}\hspace{1.66656pt}}^{(p-1)}), \quad p=1,\cdots ,P, \end{aligned}$$

where $\underline{\varvec{c\hspace{-1.66656pt}}\hspace{1.66656pt}}^{(p)}\in {\mathbb {R}}^{(I \times Q \times M)}$ consists of *M* subtimenodes components $\varvec{c\hspace{-1.66656pt}}\hspace{1.66656pt}^{m,(p)}$, each of them containing *I* DoF components $\varvec{c\hspace{-1.66656pt}}\hspace{1.66656pt}_i^{m,(p)}$. Just like in the ODE case, procedure (57) results in an explicit iterative algorithm due to the fact that the operator ${\mathcal {L}}_{\Delta }^1$ is explicit. After a direct computation, the update of the component associated to the general DoF *i* in the *m*th subtimenode at the *p*th iteration reads

58 $$\begin{aligned}  \varvec{c\hspace{-1.66656pt}}\hspace{1.66656pt}_i^{m,(p)}&=\varvec{c\hspace{-1.66656pt}}\hspace{1.66656pt}_i^{m,(p-1)} -\frac{1}{C_i}\Bigg [\sum _{K\in K_i}\sum _{\varvec{x\hspace{-1.66656pt}}\hspace{1.66656pt}_j \in K} \left( \varvec{c\hspace{-1.66656pt}}\hspace{1.66656pt}_j^{m,(p-1)}\!\!-\varvec{c\hspace{-1.66656pt}}\hspace{1.66656pt}_j^0\right) \int _K \varphi _i(\varvec{x\hspace{-1.66656pt}}\hspace{1.66656pt}) \varphi _j(\varvec{x\hspace{-1.66656pt}}\hspace{1.66656pt}) \textrm{d} \varvec{x\hspace{-1.66656pt}}\hspace{1.66656pt}\\&\quad +\Delta t \sum _{\ell =0}^{M} \theta ^m_\ell \varvec{\phi }_i(\varvec{c\hspace{-1.66656pt}}\hspace{1.66656pt}^{\ell ,(p-1)})\Bigg ].  \end{aligned}$$

We remark that also in this case we assume $\varvec{c\hspace{-1.66656pt}}\hspace{1.66656pt}_i^{m,(p)}=\varvec{c\hspace{-1.66656pt}}\hspace{1.66656pt}_i(t_n)$ whenever *p* or *m* are equal to 0. For what concerns the optimal number of iterations, analogous considerations to the ones made in the ODE case hold. Finally, it is worth observing that the resulting DeC schemes cannot be written in the RK form due to the difference between the mass matrices in ${\mathcal {L}}_{\Delta }^1$ and ${\mathcal {L}}_{\Delta }^2$. In fact, such DeC formulation is not obtained via a trivial application of the method of lines.

### bDeCu for CG

As for ODEs, it is possible to modify the original DeC for hyperbolic problems to get a new more efficient method by introducing interpolation processes between the iterations. The underlying idea is the same, and we increase the number of subtimenodes as the accuracy of the approximation increases. At the general iteration *p*, the interpolation process allows to get $\underline{\varvec{c\hspace{-1.66656pt}}\hspace{1.66656pt}}^{*(p-1)}$ from $\underline{\varvec{c\hspace{-1.66656pt}}\hspace{1.66656pt}}^{(p-1)}$ and then we perform the iteration via (58) getting

59 $$\begin{aligned} \varvec{c\hspace{-1.66656pt}}\hspace{1.66656pt}_i^{m,(p)}&=\varvec{c\hspace{-1.66656pt}}\hspace{1.66656pt}_i^{*m,(p-1)}-\frac{1}{C_i}\Bigg [\sum _{K\in K_i}\sum _{\varvec{x\hspace{-1.66656pt}}\hspace{1.66656pt}_j \in K} \left( \varvec{c\hspace{-1.66656pt}}\hspace{1.66656pt}_j^{*m,(p-1)}-\varvec{c\hspace{-1.66656pt}}\hspace{1.66656pt}_j^0\right) \int _K \varphi _i(\varvec{x\hspace{-1.66656pt}}\hspace{1.66656pt}) \varphi _j(\varvec{x\hspace{-1.66656pt}}\hspace{1.66656pt}) \textrm{d} \varvec{x\hspace{-1.66656pt}}\hspace{1.66656pt}\\&\quad +\Delta t \sum _{\ell =0}^{M} \theta ^m_\ell \varvec{\phi }_i(\varvec{c\hspace{-1.66656pt}}\hspace{1.66656pt}^{*\ell,(p-1)})\Bigg ].  \end{aligned}$$

## Application to Adaptivity

In this section, we will see how to exploit the interpolation processes in the new schemes, $\alpha$DeCu and $\alpha$DeCdu, to design adaptive methods. In the context of an original $\alpha$DeC method with a fixed number of subtimenodes, iteration by iteration, we increase the order of accuracy with respect to the solution $\underline{\varvec{u\hspace{-1.66656pt}}\hspace{1.66656pt}}_\Delta$ of the operator ${\mathcal {L}}_{\Delta }^2$. For this reason, performing a number of iterations higher than the order of accuracy of the discretization adopted in the construction of the operator ${\mathcal {L}}_{\Delta }^2$ is formally useless, as we have already pointed out in Sect. 2. Instead, in the context of an $\alpha$DeCu or $\alpha$DeCdu method, we could in principle keep adding subtimenodes, through interpolation, always improving the accuracy of the approximation with respect to the exact solution of (6), until a convergence condition on the final component of $\underline{\varvec{u\hspace{-1.66656pt}}\hspace{1.66656pt}}^{(p)}$ (always associated to $t_{n+1}$) is met, e.g.,

60 $$\begin{aligned} \frac{\left\Vert \underline{\varvec{u\hspace{-1.66656pt}}\hspace{1.66656pt}}^{p,(p)} - \underline{\varvec{u\hspace{-1.66656pt}}\hspace{1.66656pt}}^{p-1,(p-1)}\right\Vert }{\left\Vert \underline{\varvec{u\hspace{-1.66656pt}}\hspace{1.66656pt}}^{p,(p)}\right\Vert }\leqslant \varepsilon \end{aligned}$$

with $\varepsilon$ a desired tolerance. This leads to a *p*-adaptive version of the presented algorithms.

## Numerical Results

In this section, we will numerically investigate the new methods, showing the computational advantage with respect to the original ones. Since the $\alpha$DeC, $\alpha$DeCu, and $\alpha$DeCdu methods of order 2 coincide, we will focus on methods from order 3 on.

### ODE Tests

We will assess here the properties of the new methods on different ODEs tests, checking their computational costs, their errors and their adaptive versions. We will focus on the methods got for $\alpha =0$ (bDeC) and $\alpha =1$ (sDeC).

#### Linear System

The first test is a very simple $2\times 2$ system of equations

61 $$\begin{aligned} {\left\{ \begin{array}{ll} u' = -5u+v,\\ v' = 5u-v, \end{array}\right. } \qquad \begin{pmatrix} u_0\\ v_0 \end{pmatrix} = \begin{pmatrix} 0.9\\ 0.1 \end{pmatrix} \end{aligned}$$

with the exact solution $u(t)= u_0 + (1-{\rm{e}}^{-6t}) (-5u_0+v_0)$ and $v(t)=1-u(t)$. We assume a final time $T=1$.

**Fig. 4:**
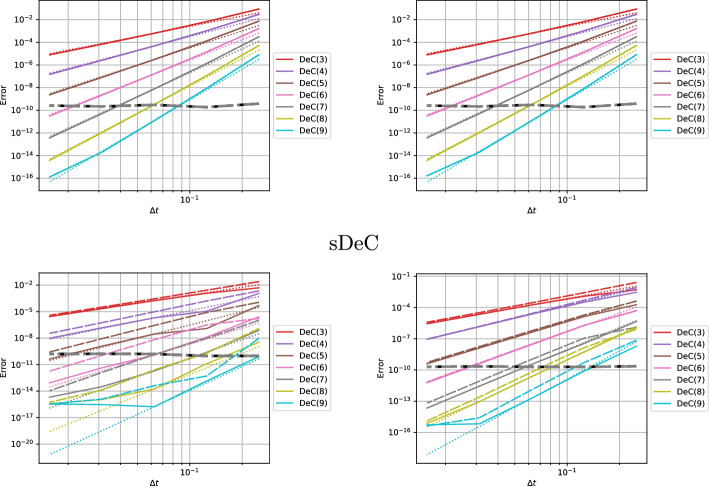
Linear system: error decay for DeC with the continuous line, DeCu with the dashed line, DeCdu with the dash-dotted line, reference order with the dotted line, adaptive DeCu with the dashed black line, and adaptive DeCdu with the dash-dotted gray line. Equispaced subtimenodes on the left and GL on the right

**Fig. 5:**
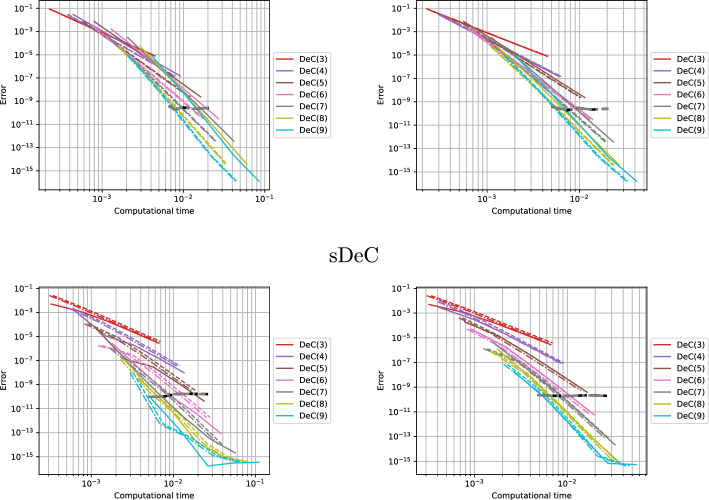
Linear system: error with respect to computational time for DeC with the continuous line, DeCu with the dashed line, DeCdu with the dash-dotted line, adaptive DeCu with the dashed black line, and adaptive DeCdu with the dash-dotted gray line. Equispaced subtimenodes on the left and GL on the right

**Fig. 6:**
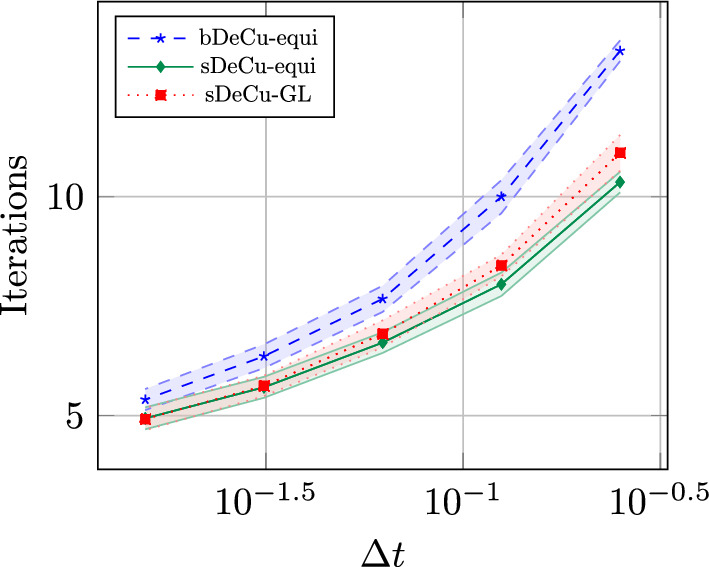
Linear test: average number of iterations (± half standard deviation) of some adaptive DeC for different time steps

**Fig. 7:**
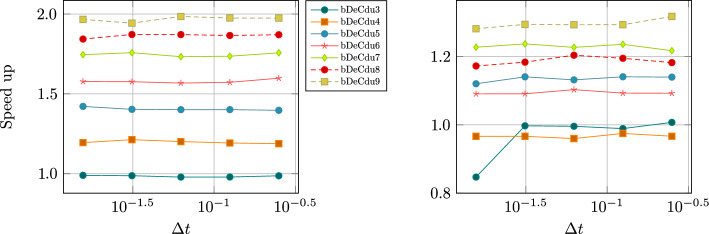
Linear system test: speed up factor for the bDeCdu method. Equispaced subtimenodes on the left and GL on the right

In Fig. 4, we plot the error decay for all methods with respect to $\Delta t$ for all orders from 3 to 9 and the expected order of convergence is achieved in all cases. We can see that the bDeC, bDeCu, and bDeCdu methods have the same error, since they coincide on linear problems, as shown in Theorem 2. The sDeC methods show a more irregular behavior and, on average, the errors with the sDeCu and sDeCdu, which coincide due to Theorem 3, are slightly larger than the one of sDeC for a fixed $\Delta t$. In Fig. 5, we plot the error against the computational time of the methods. For bDeC methods there is a huge advantage in using the novel methods: the Pareto front is composed only by the novel methods. In particular, for equispaced subtimenodes there is a larger reduction in computational cost than for GL ones, as predicted by theory. For sDeC methods the situation is not as clear as in the bDeC case. We can systematically see a difference between sDeCu and sDeCdu, being the latter more efficient than the former. In the context of GL subtimenodes, the sDeCdu is slightly better than the original sDeC method from order 5 on in the mesh refinement. We also tested the adaptive versions of the methods, characterized by the convergence criterion (60) with a tolerance $\varepsilon =10^{-8}$. As we observe in Fig. 4, the error of these methods (in black and gray) is constant and independent of $\Delta t$. The required computational time, see Fig. 5, is comparable to the one of very high order schemes. In Fig. 6, we report the average number of iterations ± half standard deviation for different adaptive methods with respect to the time discretization. As expected, the smaller the timestep, the smaller is the number of iterations necessary to reach the expected accuracy. In Fig. 7, we display, for different $\Delta t$, the speed up factor of the bDeCdu method with respect to the bDeC method computed as the ratio between the computational times required by the bDeCdu and the bDeC method. For equispaced subtimenodes we see that, as the order increases, the interpolation process reduces the computational time by an increasing factor, which is almost 2 for order 9. For GL subtimenodes the reduction is smaller but still remarkable, close to $\frac{4}{3}$ in the asymptotic limit.

#### Vibrating System

**Fig. 8:**
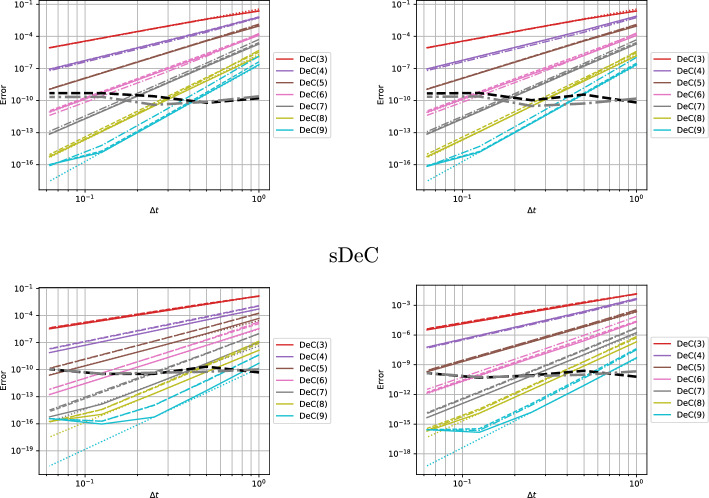
Vibrating system: error decay for DeC with the continuous line, DeCu with the dashed line, DeCdu with the dash-dotted line, reference order with the dotted line, adaptive DeCu with the dashed black line, and adaptive DeCdu with the dash-dotted gray line. Equispaced subtimenodes on the left and GL on the right

**Fig. 9:**
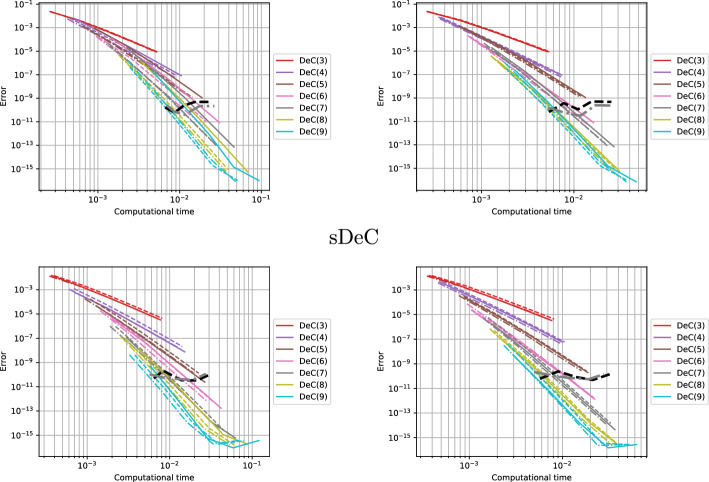
Vibrating system: error with respect to the computational time for DeC with the continuous line, DeCu with the dashed line, DeCdu with the dash-dotted line, adaptive DeCu with the dashed black line, and adaptive DeCdu with the dash-dotted gray line. Equispaced subtimenodes on the left and GL on the right

**Fig. 10:**
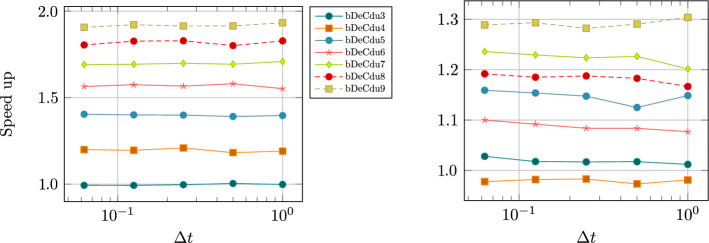
Vibrating system test: speed up factor for the bDeCdu method. Equispaced subtimenodes on the left and GL on the right

Let us consider a vibrating system defined by the following ODE:

62 $$\begin{aligned} {\left\{ \begin{array}{ll} &{} my''+ry'+ky=F\cos (\Omega t +\varphi ),\quad t \in {\mathbb {R}}^+_0,\\ &{} y(0)=A ,\\ &{} y'(0)=B \end{array}\right. } \end{aligned}$$

with $m,k,\Omega >0$, $r,F,\psi \geqslant 0$. Its exact solution [11] reads $y^{{\text{ex}}}(t)=y_h(t)+y_p(t)$ with $y_p(t)=Y_p\cos (\Omega t + \psi )$ particular solution of the whole equation characterized by

63 $$ Y_p=\frac{F}{\sqrt{(-m\Omega ^2+k)^2+\Omega ^2 r^2}},\qquad \psi=\varphi -\arg {(-m\Omega ^2+k+i\Omega r)} $$

and $y_h(t)$ general solution of the homogeneous equation

64 $$\begin{aligned} y_h(t)={\left\{ \begin{array}{ll} C_1{\rm{e}}^{\lambda _1 t}+C_2{\rm{e}}^{\lambda _2 t}, &{} \text {if }r> 2\sqrt{km},\\ C_1{\rm{e}}^{\lambda t}+C_2t{\rm{e}}^{\lambda t}, &{} \text {if } r= 2\sqrt{km},\\ {\rm{e}}^{-\frac{r}{2m} t}\left( C_1\cos (\omega t)+C_2\sin (\omega t)\right) , &{} \text {if } r< 2\sqrt{km}, \end{array}\right. } \end{aligned}$$

where $\omega =\frac{1}{2m}\sqrt{4km-r^2}$, $\lambda _1$ and $\lambda _2$ are the real roots of the characteristics polynomial associated to (62), which are equal to $\lambda$ when $r= 2\sqrt{km}$. $C_1$ and $C_2$ are two constants computed by imposing the initial conditions $y(0)=A$ and $y'(0)=B$. The mathematical steps needed to get the solution are reported in the supplementary material. The second order scalar ODE (62) can be rewritten in a standard way as a vectorial first order ODE. In the test, we have set $m=5$, $r=2$, $k=5$, $F=1$, $\Omega =2$, $\varphi =0.1$, $A=0.5$, and $B=0.25$ with a final time $T=4$. In Fig. 8, we show the error decay for all methods. Differently from the linear case, here bDeC, bDeCu, and bDeCdu are not equivalent. Nevertheless, in terms of errors, they behave in a similar way and, also comparing equispaced and GL subtimenodes, we do not observe large deviations. On average the novel schemes are slightly less accurate for a fixed $\Delta t$, even if this is not true for all orders of accuracy. For the sDeC, there is a larger difference in the errors between sDeC and sDeCu or sDeCdu, though being the order of accuracy always correct. These effects are visible also in Fig. 9. For bDeC with equispaced subtimenodes, the advantages of using the novel methods are evident: the error is almost the same and the computational time reduces by almost half for high order schemes. For bDeC methods with GL subtimenodes the computational advantage of the novel methods is not as big as the one registered in the previous case as expected from theory, see Tables 5 and 6, but still pretty visible. For what concerns the sDeC methods with equispaced subtimenodes, the performance of sDeCdu is similar to the one of sDeC until order 5, while, from order 6 on, the novel method is definitely more convenient. The sDeCu method is always less efficient than the sDeCdu one; in particular, only for very high orders it appears to be preferable to the standard method. The general trend of the sDeC methods with GL subtimenodes is that the sDeCdu and the sDeCu always perform, respectively, slightly better and slightly worse than the original sDeC. The results of the adaptive methods for this test are qualitatively similar to the ones seen in the context of the previous test: the methods produce a constant error for any $\Delta t$. Also in this case, the threshold for the relative error has been chosen equal to $10^{-8}$. Finally, in Fig. 10, we display the speed up factor of the new bDeCdu methods with respect to the original bDeC: as expected from theory, it increases with the order of accuracy.

### Hyperbolic PDE Tests

For hyperbolic PDEs, we will focus on the bDeC and the bDeCu methods with equispaced subtimenodes. The order of the DeC will be chosen to match the spatial discretization one. We will use two stabilizations discussed in [26, 27]: continuous interior penalty (CIP) and orthogonal subscale stabilization (OSS). The CIP stabilization is defined as

65 $$\begin{aligned} \varvec{ST\hspace{-1.66656pt}}\hspace{1.66656pt}_i(\varvec{u\hspace{-1.66656pt}}\hspace{1.66656pt}_h)=\sum _{f \in {\mathcal {F}}_h} \alpha ^{\text {CIP}}_{f} \int _f [\![ \nabla _{\nu _f} \varphi _i ]\!] \cdot [\![ \nabla _{\nu _f} \varvec{u\hspace{-1.66656pt}}\hspace{1.66656pt}_h ]\!] \text {d}\sigma (\varvec{x\hspace{-1.66656pt}}\hspace{1.66656pt}), \end{aligned}$$

where $\alpha ^{\text {CIP}}_{f}=\delta ^{\text {CIP}} {\bar{\rho }}_f h_f^{2}$, ${\mathcal {F}}_h$ is the set of the $(D-1)$-dimensional faces shared by two elements of ${\mathcal {T}}_h$, $[\![ \cdot ]\!]$ is the jump across the face *f*, $\nabla _{\nu _f}$ is the partial derivative in the direction $\nu _f$ normal to the face *f*, ${\bar{\rho }}_f$ is a local reference value for the spectral radius of the normal Jacobian of the flux, $h_f$ is the diameter of *f*, and $\delta ^{\text {CIP}}$ is a parameter that must be tuned.

The OSS stabilization is given by

66 $$\begin{aligned} \varvec{ST\hspace{-1.66656pt}}\hspace{1.66656pt}_i(\varvec{u\hspace{-1.66656pt}}\hspace{1.66656pt}_h)=\sum _{K \in {\mathcal {T}}_h} \alpha ^{\text {OSS}}_K \int _K \nabla _{\varvec{x\hspace{-1.66656pt}}\hspace{1.66656pt}}\varphi _i \left( \nabla _{\varvec{x\hspace{-1.66656pt}}\hspace{1.66656pt}} \varvec{u\hspace{-1.66656pt}}\hspace{1.66656pt}_h -\varvec{w\hspace{-1.66656pt}}\hspace{1.66656pt}_h\right) \text {d}\varvec{x\hspace{-1.66656pt}}\hspace{1.66656pt}, \end{aligned}$$

where $\alpha ^{\text {OSS}}_K = \delta ^{\text {OSS}} {\bar{\rho }}_K h_K$, $\varvec{w\hspace{-1.66656pt}}\hspace{1.66656pt}_h$ is the $L^2$ projection of $\nabla _{\varvec{x\hspace{-1.66656pt}}\hspace{1.66656pt}} \varvec{u\hspace{-1.66656pt}}\hspace{1.66656pt}_h$ onto $V_h^{Q\times D}$, ${\bar{\rho }}_K$ is a local reference value for the spectral radius of the normal Jacobian of the flux, $h_K$ is the diameter of *K*, and $\delta ^{\text {OSS}}$ is a parameter that must be tuned.

#### 1D Linear Advection Equation (LAE)

We consider the LAE, $$ u_t+u_x=0,$$ with periodic boundary conditions on the domain $$\Omega =[0,1]$$, the initial condition $$u_0(x)=\cos (2\uppi x)$$ and the final time $$T=1$$. The exact solution is given by $$u(x,t)=u_0(x-t)$$. For the spatial discretization, we considered three families of polynomial basis functions with degree *n*: B*n*, the Bernstein polynomials [2, 4]; P*n*, the Lagrange polynomials associated to equispaced nodes; PGL*n*, the Lagrange polynomials associated to the GL nodes [26]. For B*n* and P*n*, we used the bDeC version for hyperbolic PDEs (58) introduced by Abgrall; for PGL*n*, we adopted the bDeC formulation for ODEs (10), as, in this case, the adopted quadrature formula associated to the Lagrangian nodes leads to a high order mass lumping. For all of them, we used the CIP stabilization (65) with the coefficients $$\delta ^{\text {CIP}}$$ reported in Table 7 found in [26] to minimize the dispersion error, even if, differently from there, we assumed here a constant CFL $$=0.1$$. In particular, since the coefficients for P3 and PGL4 were not provided, we used for the former the same coefficient as for B3, while, for the latter the same coefficient as for PGL3.

**Fig. 11:**
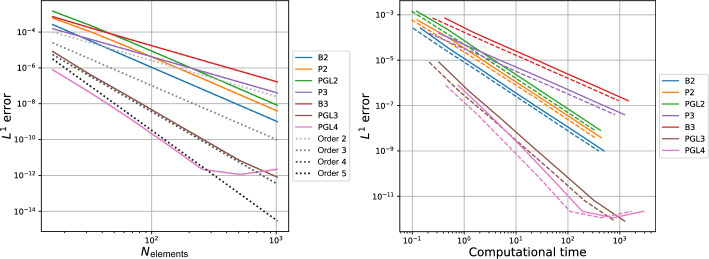
1D LAE: bDeC with continuous line, bDeCu with the dashed line, and reference order with the dotted line. Convergence analysis on the left and error with respect to computational time on the right

**Table 7:** Coefficients $\delta ^{\text {CIP}}$ used for LAE in one dimension

	B2	P2	PGL2	B3	P3$^{*}$	PGL3	PGL4$^{*}$
$\delta ^{\text {CIP}}$	0.016	0.002 42	0.003 46	0.007 02	0.007 02	0.000 113	0.000 113

$^{*}$The coefficients adopted for P3 and PGL4 are not provided in [26]

The results of the convergence analysis and of the computational cost analysis are displayed in Fig. 11. For a fixed number of elements, the errors of the bDeC and of the bDeCu methods are essentially identical, leading to a remarkable computational advantage of the novel method with respect to the original bDeC, visible in the plot on the right, where the error against the computational time is depicted. The formal order of accuracy is recovered in all the cases but for B3 and P3 for which we get only second order for both bDeC and bDeCu.

##### Remark 3

(Issues with the DeC for PDEs) The loss of accuracy for bDeC4 and B3 elements has been registered in other works, e.g., [4, 26, 27]. Even in the original paper [2], the author underlined the necessity to perform more iterations than theoretically expected for orders greater than 3 to recover the formal order of accuracy. According to authors’ opinion the problem deserves a particular attention, for this reason, the results related to B3 and P3 have not been omitted. The pathology seems to have effect only in the context of unsteady tests and it is maybe due to a *high order weak instability*. The phenomenon is currently under investigation; more details can be found in the supplementary material. However, we remark that this issue does not occur for elements that allow a proper mass lumping like PGL (or Cubature in 2D).

**Fig. 12:**
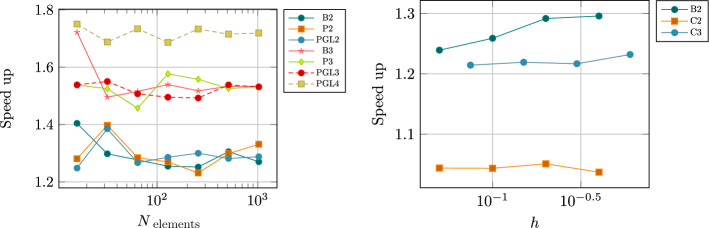
Speed up in the hyperbolic tests of bDeCu with respect to bDeC. 1D LAE on the left and 2D shallow water (SW) on the right

The speed up factor of the novel bDeCu with respect to the original method is reported in Fig. 12. The obtained speed up factors are higher than ODE ones, because in the implementation of the DeC for PDEs the major cost is not given by the flux evaluation of previously computed stages, but by the evolution of the new stages. This slightly changes the expected and the observed speed up, providing even larger computational advantages.

#### 2D Shallow Water (SW) Equations

We consider the SW equations onto $\Omega =(0,3)\times (0,3)\in {\mathbb {R}}^2$, defined, in the form (49), by

67 $$\begin{aligned} \varvec{u\hspace{-1.66656pt}}\hspace{1.66656pt}=\begin{pmatrix} H\\ H\varvec{v\hspace{-1.66656pt}}\hspace{1.66656pt}\end{pmatrix},\quad \varvec{F\hspace{-1.66656pt}}\hspace{1.66656pt}(\varvec{u\hspace{-1.66656pt}}\hspace{1.66656pt})=\begin{pmatrix} H\varvec{v\hspace{-1.66656pt}}\hspace{1.66656pt}\\ H\varvec{v\hspace{-1.66656pt}}\hspace{1.66656pt}\otimes \varvec{v\hspace{-1.66656pt}}\hspace{1.66656pt}+g\frac{H^2}{2}{\mathbb {I}} \end{pmatrix},\quad {\varvec{S}}(\varvec{x\hspace{-1.66656pt}}\hspace{1.66656pt},\varvec{u\hspace{-1.66656pt}}\hspace{1.66656pt})=0, \end{aligned}$$

where *H* is the water height, $\varvec{v\hspace{-1.66656pt}}\hspace{1.66656pt}=(v_1,v_2)^{\rm{T}}\in {\mathbb {R}}^2$ is the vertically averaged speed of the flow, *g* is the gravitational constant, ${\mathbb {I}}\in {\mathbb {R}}^{D\times D}$ is the identity matrix, and $D=2$ is the number of physical dimensions. The test is a $C^6(\Omega )$ compactly supported unsteady vortex from the collection presented in [34] given by

68 $$\begin{aligned} \varvec{u\hspace{-1.66656pt}}\hspace{1.66656pt}= \varvec{u\hspace{-1.66656pt}}\hspace{1.66656pt}^\infty + {\left\{ \begin{array}{ll} \varvec{u\hspace{-1.66656pt}}\hspace{1.66656pt}_{r_0}(r), &{} \text { if }r=||\varvec{x\hspace{-1.66656pt}}\hspace{1.66656pt}-\varvec{x\hspace{-1.66656pt}}\hspace{1.66656pt}_m(t)||_2 <r_0,\\ 0, &{} \text { else,} \end{array}\right. } \end{aligned}$$

where $$\varvec{u\hspace{-1.66656pt}}\hspace{1.66656pt}^{\infty } =(1,1,1)^{\rm{T}}$$, $$\varvec{x\hspace{-1.66656pt}}\hspace{1.66656pt}_m(t) = \varvec{x\hspace{-1.66656pt}}\hspace{1.66656pt}_c + t\cdot (1,1)^{\rm{T}}$$, and

69 $$\begin{aligned}&\varvec{u\hspace{-1.66656pt}}\hspace{1.66656pt}_{r_0}(r) = \begin{pmatrix} \frac{1}{g}\left( \frac{\Gamma }{\omega }\right) ^2 \left( \lambda (\omega r)-\lambda (\uppi )\right) \\ \Gamma \left( 1+\cos (\omega r)\right) ^2(x_2-x_{m,2})\\ - \Gamma \left( 1+\cos (\omega r)\right) ^2(x_1-x_{m,1}) \end{pmatrix},\; \Gamma = \frac{12\uppi \sqrt{g\Delta H}}{r_0\sqrt{315\uppi ^2 - 2\,048}} \end{aligned}$$

with $\omega = \frac{\uppi }{r_0}$ and the function $\lambda$ defined by

70 $$ \begin{aligned} \lambda (s)&= \frac{20}{3}\cos (s) + \frac{27}{16}\cos (s)^2 + \frac{4}{9}\cos (s)^3+ \frac{1}{16}\cos (s)^4 +\frac{20}{3}s\sin (s) \\&\quad +\frac{35}{16}s^2 + \frac{27}{8}s\cos (s)\sin (s) +\frac{4}{3}s\cos (s)^2\sin (s) + \frac{1}{4}s\cos (s)^3\sin (s). \end{aligned}$$

We set $g=9.81$, $r_0=1$, $\Delta H = 0.1$, $\varvec{x\hspace{-1.66656pt}}\hspace{1.66656pt}_c=(1,1)^{\rm{T}}$ with a final time $T=1$ and Dirichlet boundary conditions. For the spatial discretization, we considered two basis functions: B*n*, the Bernstein polynomials; C*n*, the Cubature elements introduced in [15]. As they allow a high order mass lumping, for C*n* elements we used the bDeC (10) for ODEs and OSS stabilization (66), instead, for B*n* we considered the PDE formulation (58) and CIP stabilization (65). The tests with B2 have been run with $\text {CFL}=0.1$ and $\delta ^{\text {CIP}}=0.04$; for C2 elements we have set $\text {CFL}=0.12$ and $\delta ^{\text {OSS}}=0.07$, the optimal coefficients minimizing the dispersion error of the original bDeC according to the linear analysis performed in [27]; for C3 we adopted $\text {CFL}=0.015$ and $\delta ^{\text {OSS}}=0.2$.

**Fig. 13:**
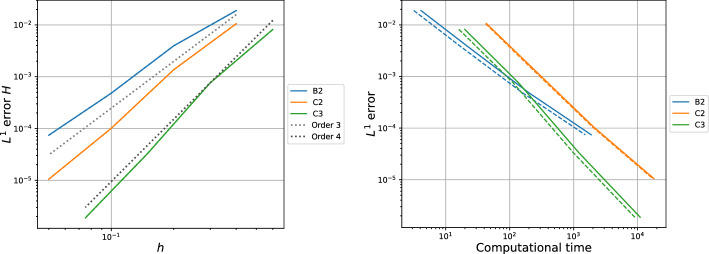
2D SW: bDeC with the continuous line, bDeCu with the dashed line, and reference order with the dotted line. Convergence analysis on the left and error with respect to computational time on the right

The results of the convergence analysis and of the computational cost analysis are displayed in Fig. 13. The errors produced by the novel and the original bDeC method are so close that the lines coincide. The resulting computational advantage can be seen in the plot on the right. The formal order of accuracy is recovered in all the cases and the speed up factor, in Fig. 12, proves the convenience in using the novel bDeCu formulation instead of the original bDeC. Let us observe that, according to Table 5, the number of stages of bDeC3 and bDeCu3 is identical, nevertheless, as observed in Remark 1, the number of stages does not strictly correspond to the computational time. If we do not consider the “cheap” stages computed via interpolation, we get the theoretical speed up factor $\frac{5}{4}=1.25$, which is what we obtained in the numerical test for B2. We conclude this section with one last observation: the computational advantage registered with B2 is much higher with respect to C2 and C3 ones, because we have run the simulations with different codes: the results obtained with B2 are obtained with a Fortran implementation, while, for C2 and C3 we have used Parasol, a Python implementation developed by Michel et al. [27] and kindly provided to us. A more careful implementation would increase further the speed up factors associated to such elements.

## Conclusions and Further Developments

In this work, we have investigated analytical and numerical aspects of two novel families of efficient explicit DeC methods. The novel methods are constructed by introducing interpolation processes between the iterations, which increase the degree of the discretization to match the accuracy of the approximation associated to the iterations. In particular, we proved that for some of the novel methods the stability region coincides with the one of the original methods. The novel methods have been tested on classical benchmarks in the ODE context revealing, in most of the cases, a remarkable computational advantage with respect to the original ones. Furthermore, the interpolation strategies have been used to design adaptive schemes. Finally, we successfully proved the good performances of the novel methods in the context of the numerical solution of hyperbolic PDEs with continuous space discretizations. Overall, we believe that the approach proposed in this work can alleviate the computational costs not only of DeC methods but also of other schemes with a similar structure. For this reason, investigations of other numerical frameworks are planned and, in particular, we are working on applications to hyperbolic PDEs (with FV and ADER schemes), in which also the order of the space reconstruction is gradually increased iteration by iteration. We hope to spread broadly this technique in the community to save computational time and resources in the numerical solution of differential problems, as only little effort is required to embed the novel modification in an existing DeC code.

## Electronic supplementary material

Below is the link to the electronic supplementary material.

Supplementary material 1 The interested reader is referred to the supplementary material for all the proofs omitted in this document for the sake of compactness. (PDF 562 kb)

## Data Availability

On behalf of all authors, the corresponding author is available to collect documentation of compliance with ethical standards and send upon request.

## Appendix A: Residual Formulations

Here, we report the residual formulations of the original bDeC and sDeC methods presented in Sect. 3. In particular, we will present the spectral DeC formulation in terms of residuals introduced in [16] and prove that it is equivalent to the sDeC method. Then, we will see how to get, with a little modification of the presented spectral DeC formulation, the residual formulation of the bDeC method.

### Appendix A.1: Link Between Spectral DeC and sDeC

We want to solve system (6) in the interval $[t_n,t_{n+1}]$ getting $\varvec{u\hspace{-1.66656pt}}\hspace{1.66656pt}_{n+1}\approx \varvec{u\hspace{-1.66656pt}}\hspace{1.66656pt}(t_{n+1})$ from $\varvec{u\hspace{-1.66656pt}}\hspace{1.66656pt}_{n}=\varvec{u\hspace{-1.66656pt}}\hspace{1.66656pt}(t_{n})$. Also in this case, we consider an iterative procedure on the approximated values $\varvec{u\hspace{-1.66656pt}}\hspace{1.66656pt}^{m,(p)}$ of the solution in the subtimenodes $m=1,\cdots ,M$, collected in the vector $\underline{\varvec{u\hspace{-1.66656pt}}\hspace{1.66656pt}}^{(p)}$, with $\varvec{u\hspace{-1.66656pt}}\hspace{1.66656pt}^{0,(p)}:=\varvec{u\hspace{-1.66656pt}}\hspace{1.66656pt}_{n}$ fixed. Given $\underline{\varvec{u\hspace{-1.66656pt}}\hspace{1.66656pt}}^{(p-1)}$, we consider the interpolation polynomial $\varvec{{\mathcal {I}}\hspace{-1.66656pt}}\hspace{1.66656pt}(\underline{\varvec{u\hspace{-1.66656pt}}\hspace{1.66656pt}}^{(p-1)},t):=\sum _{m=0}^M \varvec{u\hspace{-1.66656pt}}\hspace{1.66656pt}^{m,(p-1)}\psi ^m(t)$. The spectral DeC relies on the definition at each iteration *p* of two support variables, namely the error function $\varvec{e\hspace{-1.66656pt}}\hspace{1.66656pt}^{(p)}(t)$ with respect to the exact solution and the residual function $\varvec{r\hspace{-1.66656pt}}\hspace{1.66656pt}^{(p-1)}(t)$ respectively given by

A1 $$\begin{aligned} \varvec{e\hspace{-1.66656pt}}\hspace{1.66656pt}^{(p)}(t)&:=\varvec{u\hspace{-1.66656pt}}\hspace{1.66656pt}(t)-\varvec{{\mathcal {I}}\hspace{-1.66656pt}}\hspace{1.66656pt}(\underline{\varvec{u\hspace{-1.66656pt}}\hspace{1.66656pt}}^{(p-1)},t), \end{aligned}$$

A2 $$\begin{aligned} \varvec{r\hspace{-1.66656pt}}\hspace{1.66656pt}^{(p-1)}(t)&:=\varvec{u\hspace{-1.66656pt}}\hspace{1.66656pt}_n+\int _{t_n}^{t}\varvec{G\hspace{-1.66656pt}}\hspace{1.66656pt}(s,\varvec{{\mathcal {I}}\hspace{-1.66656pt}}\hspace{1.66656pt}(\underline{\varvec{u\hspace{-1.66656pt}}\hspace{1.66656pt}}^{(p-1)},s))\textrm{d}s -\varvec{{\mathcal {I}}\hspace{-1.66656pt}}\hspace{1.66656pt}(\underline{\varvec{u\hspace{-1.66656pt}}\hspace{1.66656pt}}^{(p-1)},t). \end{aligned}$$

By integrating the original ODE (6), making use of the definitions of the error function $\varvec{e\hspace{-1.66656pt}}\hspace{1.66656pt}^{(p)}(t)$ and of the residual function $\varvec{r\hspace{-1.66656pt}}\hspace{1.66656pt}^{(p-1)}(t)$ and differentiating again, we get that the error function satisfies the ODE

A3 $$\begin{aligned} {\left\{ \begin{array}{ll} \frac{\textrm{d}}{\textrm{d}t}\varvec{e\hspace{-1.66656pt}}\hspace{1.66656pt}^{(p)}(t)=\varvec{G\hspace{-1.66656pt}}\hspace{1.66656pt}(t,\varvec{{\mathcal {I}}\hspace{-1.66656pt}}\hspace{1.66656pt}(\underline{\varvec{u\hspace{-1.66656pt}}\hspace{1.66656pt}}^{(p-1)},t)+\varvec{e\hspace{-1.66656pt}}\hspace{1.66656pt}^{(p)}(t))-\varvec{G\hspace{-1.66656pt}}\hspace{1.66656pt}(t,\varvec{{\mathcal {I}}\hspace{-1.66656pt}}\hspace{1.66656pt}(\underline{\varvec{u\hspace{-1.66656pt}}\hspace{1.66656pt}}^{(p-1)},t))+\frac{\textrm{d}}{\textrm{d}t}\varvec{r\hspace{-1.66656pt}}\hspace{1.66656pt}^{(p-1)}(t),\\ \varvec{e\hspace{-1.66656pt}}\hspace{1.66656pt}^{(p)}(t_n)=0. \end{array}\right. } \end{aligned}$$

We can numerically solve such an ODE in each subinterval $[t^{m-1},t^{m}]$ through the explicit Euler method starting from $m=1$ on, thus getting

A4 $$\begin{aligned} \varvec{e\hspace{-1.66656pt}}\hspace{1.66656pt}^{m,(p)}&=\varvec{e\hspace{-1.66656pt}}\hspace{1.66656pt}^{m-1,(p)} + \Delta t \gamma ^m [ \varvec{G\hspace{-1.66656pt}}\hspace{1.66656pt}(t^{m-1},\varvec{u\hspace{-1.66656pt}}\hspace{1.66656pt}^{m-1,(p-1)}+\varvec{e\hspace{-1.66656pt}}\hspace{1.66656pt}^{m-1,(p)})\\&\quad  -\varvec{G\hspace{-1.66656pt}}\hspace{1.66656pt}(t^{m-1},\varvec{u\hspace{-1.66656pt}}\hspace{1.66656pt}^{m-1,(p-1)}) ] +\varvec{r\hspace{-1.66656pt}}\hspace{1.66656pt}^{m,(p-1)}-\varvec{r\hspace{-1.66656pt}}\hspace{1.66656pt}^{m-1,(p-1)}  \end{aligned}$$

with the integrals in the residual function approximated through a spectral integration, i.e., $\varvec{r\hspace{-1.66656pt}}\hspace{1.66656pt}^{m,(p-1)}:=\varvec{u\hspace{-1.66656pt}}\hspace{1.66656pt}_n+\int _{t_n}^{t^m}\sum _{\ell =0}^M \varvec{G\hspace{-1.66656pt}}\hspace{1.66656pt}(t^{\ell },\varvec{u\hspace{-1.66656pt}}\hspace{1.66656pt}^{\ell ,(p-1)}) \psi ^{\ell }(t) {\rm{d}}t-\varvec{u\hspace{-1.66656pt}}\hspace{1.66656pt}^{m,(p-1)}$. We have used for $\varvec{e\hspace{-1.66656pt}}\hspace{1.66656pt}^{m,(p)}$ and $\varvec{r\hspace{-1.66656pt}}\hspace{1.66656pt}^{m,(p-1)}$ the usual convention adopted throughout the manuscript, with *m* standing for the subtimenode $t^m$ to which such quantities are associated. Indeed, we have $\varvec{e\hspace{-1.66656pt}}\hspace{1.66656pt}^{0,(p)}=\varvec{0\hspace{-1.66656pt}}\hspace{1.66656pt}$ and $\varvec{r\hspace{-1.66656pt}}\hspace{1.66656pt}^{0,(p-1)}=\varvec{0\hspace{-1.66656pt}}\hspace{1.66656pt}$. The computed errors are then used to get new approximated values of the solution $\varvec{u\hspace{-1.66656pt}}\hspace{1.66656pt}^{m,(p)}:=\varvec{u\hspace{-1.66656pt}}\hspace{1.66656pt}^{m,(p-1)}+\varvec{e\hspace{-1.66656pt}}\hspace{1.66656pt}^{m,(p)}$, allowing to repeat the described process with new error and residual functions, $\varvec{e\hspace{-1.66656pt}}\hspace{1.66656pt}^{(p+1)}(t)$ and $\varvec{r\hspace{-1.66656pt}}\hspace{1.66656pt}^{(p)}(t)$, analogously defined. The procedure gains one order of accuracy at each iteration until the accuracy of the discretization is saturated and, at the end of the iteration process with *P* iterations, one can set $\varvec{u\hspace{-1.66656pt}}\hspace{1.66656pt}_{n+1}:=\varvec{u\hspace{-1.66656pt}}\hspace{1.66656pt}^{M,(P)}$. By explicit computation, we have that (A4) is equivalent to the sDeC updating formula (13). In fact, recalling the definition of $\varvec{u\hspace{-1.66656pt}}\hspace{1.66656pt}^{m,(p)}$ and $\varvec{r\hspace{-1.66656pt}}\hspace{1.66656pt}^{m,(p-1)}$, we get

A5 $$\begin{aligned} \varvec{e\hspace{-1.66656pt}}\hspace{1.66656pt}^{m,(p)}&=\varvec{e\hspace{-1.66656pt}}\hspace{1.66656pt}^{m-1,(p)} + \Delta t \gamma ^m \left[ \varvec{G\hspace{-1.66656pt}}\hspace{1.66656pt}(t^{m-1},\varvec{u\hspace{-1.66656pt}}\hspace{1.66656pt}^{m-1,(p)})-\varvec{G\hspace{-1.66656pt}}\hspace{1.66656pt}(t^{m-1},\varvec{u\hspace{-1.66656pt}}\hspace{1.66656pt}^{m-1,(p-1)}) \right] \\&\quad +\int _{t^{m-1}}^{t^m}\sum _{\ell =0}^M \varvec{G\hspace{-1.66656pt}}\hspace{1.66656pt}(t^{\ell 
},\varvec{u\hspace{-1.66656pt}}\hspace{1.66656pt}^{\ell ,(p-1)}) \psi ^{\ell }(t) {\rm{d}}t-\varvec{u\hspace{-1.66656pt}}\hspace{1.66656pt}^{m,(p-1)}+\varvec{u\hspace{-1.66656pt}}\hspace{1.66656pt}^{m-1,(p-1)}, \end{aligned}$$

from which, recalling the definition of $\delta _{\ell }^m$, follows

A6 $$\begin{aligned} \varvec{u\hspace{-1.66656pt}}\hspace{1.66656pt}^{m,(p)}&=\varvec{u\hspace{-1.66656pt}}\hspace{1.66656pt}^{m-1,(p)} + \Delta t \gamma ^m \left[ \varvec{G\hspace{-1.66656pt}}\hspace{1.66656pt}(t^{m-1},\varvec{u\hspace{-1.66656pt}}\hspace{1.66656pt}^{m-1,(p)})-\varvec{G\hspace{-1.66656pt}}\hspace{1.66656pt}(t^{m-1},\varvec{u\hspace{-1.66656pt}}\hspace{1.66656pt}^{m-1,(p-1)}) \right] \\&\quad +\Delta t\sum _{\ell =0}^M \delta _\ell ^m \varvec{G\hspace{-1.66656pt}}\hspace{1.66656pt}(t^{\ell },\varvec{u\hspace{-1.66656pt}}\hspace{1.66656pt}^{\ell ,(p-1)}). \end{aligned}$$

### Appendix A.2: bDeC

The residual formulation of the bDeC method is obtained in a similar way. Keeping the same definitions of $\varvec{{\mathcal {I}}\hspace{-1.66656pt}}\hspace{1.66656pt}(\underline{\varvec{u\hspace{-1.66656pt}}\hspace{1.66656pt}}^{(p-1)},t)$, $\varvec{e\hspace{-1.66656pt}}\hspace{1.66656pt}^{(p)}(t)$, and $\varvec{r\hspace{-1.66656pt}}\hspace{1.66656pt}^{(p-1)}(t)$, we have that (A3) still holds. We solve it through the explicit Euler method in each subinterval $[t^{0},t^{m}]$ obtaining

A7 $$\begin{aligned} \varvec{e\hspace{-1.66656pt}}\hspace{1.66656pt}^{m,(p)}&=\varvec{e\hspace{-1.66656pt}}\hspace{1.66656pt}^{0,(p)} + \Delta t \beta ^m \left[ \varvec{G\hspace{-1.66656pt}}\hspace{1.66656pt}(t^{0},\varvec{u\hspace{-1.66656pt}}\hspace{1.66656pt}^{0,(p-1)}+\varvec{e\hspace{-1.66656pt}}\hspace{1.66656pt}^{0,(p)})-\varvec{G\hspace{-1.66656pt}}\hspace{1.66656pt}(t^{0},\varvec{u\hspace{-1.66656pt}}\hspace{1.66656pt}^{0,(p-1)}) \right] \\&\quad +\varvec{r\hspace{-1.66656pt}}\hspace{1.66656pt}^{m,(p-1)}-\varvec{r\hspace{-1.66656pt}}\hspace{1.66656pt}^{0,(p-1)} \end{aligned}$$

with the same definition for $\varvec{r\hspace{-1.66656pt}}\hspace{1.66656pt}^{m,(p-1)}$ through spectral integration. This is the residual formulation of the bDeC method. Recalling that $\varvec{e\hspace{-1.66656pt}}\hspace{1.66656pt}^{0,(p)}=\varvec{r\hspace{-1.66656pt}}\hspace{1.66656pt}^{0,(p-1)}=\varvec{0\hspace{-1.66656pt}}\hspace{1.66656pt}$, we get

A8 $$\begin{aligned} \varvec{e\hspace{-1.66656pt}}\hspace{1.66656pt}^{m,(p)}=\varvec{r\hspace{-1.66656pt}}\hspace{1.66656pt}^{m,(p-1)}=\varvec{u\hspace{-1.66656pt}}\hspace{1.66656pt}_n+\int _{t_n}^{t^m}\sum _{\ell =0}^M \varvec{G\hspace{-1.66656pt}}\hspace{1.66656pt}(t^{\ell },\varvec{u\hspace{-1.66656pt}}\hspace{1.66656pt}^{\ell ,(p-1)}) \psi ^{\ell }(t) {\rm{d}}t-\varvec{u\hspace{-1.66656pt}}\hspace{1.66656pt}^{m,(p-1)}, \end{aligned}$$

from which, recalling the definition of $\varvec{u\hspace{-1.66656pt}}\hspace{1.66656pt}^{m,(p)}$ and of $\theta _{\ell }^m$, finally follows

A9 $$\begin{aligned} \varvec{u\hspace{-1.66656pt}}\hspace{1.66656pt}^{m,(p)}=\varvec{u\hspace{-1.66656pt}}\hspace{1.66656pt}_n+\Delta t\sum _{\ell =0}^M \theta _{\ell }^m \varvec{G\hspace{-1.66656pt}}\hspace{1.66656pt}(t^{\ell },\varvec{u\hspace{-1.66656pt}}\hspace{1.66656pt}^{\ell ,(p-1)}), \end{aligned} $$

which is nothing but (10).
