# Supplementary material for: A New Efficient Explicit Deferred Correction Framework: Analysis and Applications to Hyperbolic PDEs and Adaptivity
Source: Commun Appl Math Comput. 2023 Sep 12;6(3):1629–64. doi: 10.1007/s42967-023-00294-6 (PMC11637013; doi:10.1007/s42967-023-00294-6)
Supplement: Supplementary file 1 — Supplementary material 1 The interested reader is referred to the supplementary material for all the proofs omitted in this document for the sake of compactness. (PDF 562 kb) [file 42967_2023_294_MOESM1_ESM.pdf]

A new efficient explicit Deferred Correction framework: analysis and applications to hyperbolic PDEs and adaptivity Supplementary Material*

L. Micalizzi†and D. Torlo‡

May 17, 2023

Introduction

In this supplementary material, we show the proofs and the details that were too lengthy to be put in the principal manuscript. We show the proof of the Deferred Correction procedure in a general framework in section 1. In section 2, we provide the proofs of the accuracy and of the properties of the operators $\mathcal{L}^2_\Delta$ and $\mathcal{L}^1_\Delta$ of the bDeC method in the context of ODEs, and we show how the sDeC method can be seen as a perturbation of the bDeC. In section 3, we prove the properties of the operators $\mathcal{L}^2_\Delta$ and $\mathcal{L}^1_\Delta$ of the bDeC formulation for the continuous Galerkin (CG) finite element framework and we investigate the issues experienced in many works with such formulation. Finally, in section 4, we show how to find the analytical solution to the ODE modeling a monodimensional vibrating system.

For each section, we recall the basic notions of the main document needed for the discussion, in order to make this document as much self-contained as possible, and sometimes deepened, in order to increase the understandability.

1 Abstract DeC formulation

Assume that we have two operators, depending on the same parameter Δ, between two normed vector spaces $(X, \|\cdot\|_X)$ and $(Y, \|\cdot\|_Y)$

$$\mathcal{L}^1_\Delta, \mathcal{L}^2_\Delta : X \longrightarrow Y, \tag{1}$$

associated to two discretizations of the same problem. Then, the following theorem holds.

Theorem 1.1 (Deferred Correction accuracy)**.** *Let the following hypotheses hold*

1. ***Existence of a unique solution to $\mathcal{L}^2_\Delta$***
   $\exists!\, \underline{\boldsymbol{u}}_\Delta \in X$ *solution of* $\mathcal{L}^2_\Delta$ *such that* $\mathcal{L}^2_\Delta(\underline{\boldsymbol{u}}_\Delta) = \mathbf{0}_Y$*;*

*Main document submitted to Communications on Applied Mathematics and Computation.

†Corresponding author. Affiliation: Institute of Mathematics, University of Zurich, Winterthurerstrasse 190, Zurich, 8057, Switzerland. Email: lorenzo.micalizzi@math.uzh.ch.

‡Affiliation: SISSA mathLab, SISSA, via Bonomea 265, Trieste, 34136, Italy. Email: davide.torlo@sissa.it.

2. ***Coercivity-like property of*** $\mathcal{L}^1_\Delta$
$\exists\,\alpha_1 \geq 0$ *independent of* Δ *s.t.*

$$\left\|\mathcal{L}^1_\Delta(\underline{\boldsymbol{v}})-\mathcal{L}^1_\Delta(\underline{\boldsymbol{w}})\right\|_Y \geq \alpha_1 \left\|\underline{\boldsymbol{v}}-\underline{\boldsymbol{w}}\right\|_X, \quad \forall \underline{\boldsymbol{v}},\underline{\boldsymbol{w}} \in X; \tag{2}$$

3. ***Lipschitz-continuity-like property of*** $\mathcal{L}^1_\Delta - \mathcal{L}^2_\Delta$
$\exists\,\alpha_2 \geq 0$ *independent of* Δ *s.t.*

$$\left\|\left(\mathcal{L}^1_\Delta(\underline{\boldsymbol{v}})-\mathcal{L}^2_\Delta(\underline{\boldsymbol{v}})\right)-\left(\mathcal{L}^1_\Delta(\underline{\boldsymbol{w}})-\mathcal{L}^2_\Delta(\underline{\boldsymbol{w}})\right)\right\|_Y \leq \alpha_2 \Delta \left\|\underline{\boldsymbol{v}}-\underline{\boldsymbol{w}}\right\|_X, \quad \forall \underline{\boldsymbol{v}},\underline{\boldsymbol{w}} \in X. \tag{3}$$

Then, if we iteratively define $\underline{\boldsymbol{u}}^{(p)}$ *as the solution of*

$$\mathcal{L}^1_\Delta(\underline{\boldsymbol{u}}^{(p)}) = \mathcal{L}^1_\Delta(\underline{\boldsymbol{u}}^{(p-1)}) - \mathcal{L}^2_\Delta(\underline{\boldsymbol{u}}^{(p-1)}), \quad p = 1,\dots,P, \tag{4}$$

we have that

$$\left\|\underline{\boldsymbol{u}}^{(P)}-\underline{\boldsymbol{u}}_\Delta\right\|_X \leq \left(\Delta\frac{\alpha_2}{\alpha_1}\right)^P \left\|\underline{\boldsymbol{u}}^{(0)}-\underline{\boldsymbol{u}}_\Delta\right\|_X. \tag{5}$$

Proof. By using the coercivity-like property of $\mathcal{L}^1_\Delta$ and the definition of $\mathcal{L}^1_\Delta(\underline{\boldsymbol{u}}^{(p)})$ in (4), we have

$$\left\|\underline{\boldsymbol{u}}^{(P)}-\underline{\boldsymbol{u}}_\Delta\right\|_X \leq \frac{1}{\alpha_1}\left\|\mathcal{L}^1_\Delta(\underline{\boldsymbol{u}}^{(P)})-\mathcal{L}^1_\Delta(\underline{\boldsymbol{u}}_\Delta)\right\|_Y = \frac{1}{\alpha_1}\left\|\mathcal{L}^1_\Delta(\underline{\boldsymbol{u}}^{(P-1)})-\mathcal{L}^2_\Delta(\underline{\boldsymbol{u}}^{(P-1)})-\mathcal{L}^1_\Delta(\underline{\boldsymbol{u}}_\Delta)\right\|_Y. \tag{6}$$

Since $\underline{\boldsymbol{u}}_\Delta$ is the solution of $\mathcal{L}^2_\Delta$, we have that $\mathcal{L}^2_\Delta(\underline{\boldsymbol{u}}_\Delta) = \mathbf{0}_Y$ and we can add it inside the norm on the right hand side of the equality in (6) and we get

$$\left\|\underline{\boldsymbol{u}}^{(P)}-\underline{\boldsymbol{u}}_\Delta\right\|_X \leq \frac{1}{\alpha_1}\left\|\left[\mathcal{L}^1_\Delta(\underline{\boldsymbol{u}}^{(P-1)})-\mathcal{L}^2_\Delta(\underline{\boldsymbol{u}}^{(P-1)})\right]-\left[\mathcal{L}^1_\Delta(\underline{\boldsymbol{u}}_\Delta)-\mathcal{L}^2_\Delta(\underline{\boldsymbol{u}}_\Delta)\right]\right\|_Y. \tag{7}$$

Now, by applying the Lipschitz-continuity-like property we get

$$\left\|\underline{\boldsymbol{u}}^{(P)}-\underline{\boldsymbol{u}}_\Delta\right\|_X \leq \Delta\frac{\alpha_2}{\alpha_1}\left\|\underline{\boldsymbol{u}}^{(P-1)}-\underline{\boldsymbol{u}}_\Delta\right\|_X. \tag{8}$$

By repeating these calculations recursively we get the thesis. □

2 The Deferred Correction for systems of ODEs

We will focus on the numerical solution of the general Cauchy problem

$$\begin{cases} \frac{d}{dt}\boldsymbol{u}(t) = \boldsymbol{G}(t,\boldsymbol{u}(t)), & t \in [0,T], \\ \boldsymbol{u}(0) = \boldsymbol{z}, \end{cases} \tag{9}$$

with $\boldsymbol{u}(t) \in \mathbb{R}^Q$, $\boldsymbol{z} \in \mathbb{R}^Q$ and $\boldsymbol{G} : \mathbb{R}^+_0 \times \mathbb{R}^Q \to \mathbb{R}^Q$ a continuous map Lipschitz continuous with respect to $\boldsymbol{u}$ uniformly with respect to t with a Lipschitz constant L. This ensures the existence of a unique solution for the system of ODEs (9).

We will assume here a classical one-step method setting: we discretize the time domain $[0,T]$ by introducing $N+1$ time nodes t_n, which are such that $0 = t_0 < t_1 < \dots < t_N = T$ and therefore inducing N intervals $[t_n, t_{n+1}]$, we denote by $\boldsymbol{u}_n$ an approximation of the exact solution $\boldsymbol{u}(t_n)$ at the time t_n and we look for a recipe to compute $\boldsymbol{u}_{n+1}$ by knowing $\boldsymbol{u}_n$ for each $n = 0,1,\dots,N-1$. We will focus on the generic time interval $[t_n, t_{n+1}]$ with $\Delta t = t_{n+1} - t_n$ and, as in the context of a general consistency analysis, we will assume $\boldsymbol{u}_n = \boldsymbol{u}(t_n)$.

2.1 bDeC

In the general time step $[t_n, t_n+\Delta t]$ we introduce $M+1$ subtimenodes $t^0, \dots, t^M$ such that $t_n = t^0 < t^1 < \dots < t^M = t_n + \Delta t$, which are assumed here to be equispaced. We will refer to $\boldsymbol{u}(t^m)$ as the exact solution in the node t^m and to $\boldsymbol{u}^m$ as the approximation of the solution in the same node. Just for the first node, we set $\boldsymbol{u}^0 := \boldsymbol{u}_n$ and, in the accuracy study, we will consider it to be exact, i.e., $\boldsymbol{u}^0 = \boldsymbol{u}(t^0) = \boldsymbol{u}(t_n) = \boldsymbol{u}_n$.

2.1.1 Definition of $\mathcal{L}^2_\Delta$

An exact integration of the system of ODEs over $[t^0, t^m]$ would result in

$$\boldsymbol{u}(t^m) - \boldsymbol{u}^0 - \int_{t^0}^{t^m} \boldsymbol{G}(t, \boldsymbol{u}(t))dt = \boldsymbol{0}, \quad \forall m = 1, \dots, M, \tag{10}$$

from which we would have the exact solution $\boldsymbol{u}(t^m)$.

Unfortunately, we cannot perform in general the exact integration and we need to make some approximations. We replace $\boldsymbol{G}(t, \boldsymbol{u}(t))$ by the Lagrange interpolating polynomial of degree M associated to the $M+1$ nodes t^m with $m = 0, 1, \dots, M$, getting

$$\boldsymbol{u}^m - \boldsymbol{u}^0 - \int_{t^0}^{t^m} \sum_{\ell=0}^{M} \boldsymbol{G}(t^\ell, \boldsymbol{u}(t^\ell))\psi^\ell(t)dt = \boldsymbol{0}, \quad \forall m = 1, \dots, M. \tag{11}$$

Moving the finite sum and the vectors $\boldsymbol{G}(t^\ell, \boldsymbol{u}(t^\ell))$ outside of the integral, (11) can be recast as

$$\boldsymbol{u}^m - \boldsymbol{u}^0 - \Delta t \sum_{\ell=0}^{M} \theta_\ell^m \boldsymbol{G}(t^\ell, \boldsymbol{u}(t^\ell)) = \boldsymbol{0}, \quad \forall m = 1, \dots, M, \tag{12}$$

where the coefficients θ_ℓ^m are the normalized integrals of the Lagrange basis functions and do not depend on Δt.

Proposition 2.1. *$\boldsymbol{u}^m$ satisfying* (12) *is an $(M+1)$-th order accurate approximation of $\boldsymbol{u}(t^m)$.*

Proof. For the proof, we will focus on the original equivalent formulation (11). Let us compute $\boldsymbol{u}(t^m) - \boldsymbol{u}^m$ with $\boldsymbol{u}^m$ got by (11). From (10), (11) and the M-th order accuracy on the approximation of $\boldsymbol{G}(t, \boldsymbol{u}(t))$ due to the interpolation with Lagrange polynomials of degree M we have

$$\begin{aligned}
\boldsymbol{u}(t^m) - \boldsymbol{u}^m &= \boldsymbol{u}^0 + \int_{t^0}^{t^m} \boldsymbol{G}(t, \boldsymbol{u}(t))dt - \boldsymbol{u}^0 - \int_{t^0}^{t^m} \sum_{\ell=0}^{M} \boldsymbol{G}(t^\ell, \boldsymbol{u}(t^\ell))\psi^\ell(t)dt \\
&= \int_{t^0}^{t^m} \left[\boldsymbol{G}(t, \boldsymbol{u}(t)) - \sum_{\ell=0}^{M} \boldsymbol{G}(t^\ell, \boldsymbol{u}(t^\ell))\psi^\ell(t) \right] dt \\
&= \int_{t^0}^{t^m} O(\Delta t^{M+1})dt = O(\Delta t^{M+2}).
\end{aligned} \tag{13}$$

□

Despite this result, the previous formula cannot be used in practice because the exact solution $\boldsymbol{u}(t^\ell)$ in the nodes t^ℓ with $\ell = 1, \dots, M$ is not available.

We use the approximated values $\boldsymbol{u}^\ell$ in place of them, thus getting the following implicit formulation

$$\boldsymbol{u}^m - \boldsymbol{u}^0 - \Delta t \sum_{\ell=0}^{M} \theta_\ell^m \boldsymbol{G}(t^\ell, \boldsymbol{u}^\ell) = \boldsymbol{0} \quad \forall m = 1, \dots, M, \tag{14}$$

which leads to the definition of our $\mathcal{L}^2_\Delta$ operator

$$\mathcal{L}^2_\Delta(\underline{\boldsymbol{u}}) = \begin{pmatrix} \boldsymbol{u}^1 - \boldsymbol{u}^0 - \Delta t \sum_{\ell=0}^{M} \theta_\ell^1 \boldsymbol{G}(t^\ell, \boldsymbol{u}^\ell) \\ \vdots \\ \boldsymbol{u}^m - \boldsymbol{u}^0 - \Delta t \sum_{\ell=0}^{M} \theta_\ell^m \boldsymbol{G}(t^\ell, \boldsymbol{u}^\ell) \\ \vdots \\ \boldsymbol{u}^M - \boldsymbol{u}^0 - \Delta t \sum_{\ell=0}^{M} \theta_\ell^M \boldsymbol{G}(t^\ell, \boldsymbol{u}^\ell) \end{pmatrix} \text{ with } \underline{\boldsymbol{u}} = \begin{pmatrix} \boldsymbol{u}^1 \\ \vdots \\ \boldsymbol{u}^m \\ \vdots \\ \boldsymbol{u}^M \end{pmatrix}. \tag{15}$$

Proposition 2.2. *Let $\boldsymbol{u}^m$ be the m-th component of the solution of $\mathcal{L}^2_\Delta(\underline{\boldsymbol{u}}) = 0$. Then, $\boldsymbol{u}^m$ is an $(M+1)$-th order accurate approximation of $\mathbf{u}(t^m)$.*

Proof. Let us consider the following operator $\mathcal{J} : \mathbb{R}^{(M \times Q)} \to \mathbb{R}^{(M \times Q)}$ defined as

$$\underline{\boldsymbol{y}} = \mathcal{J}(\underline{\boldsymbol{u}}) = \begin{pmatrix} \boldsymbol{u}^0 + \Delta t \sum_{\ell=0}^{M} \theta_\ell^1 \boldsymbol{G}(t^\ell, \boldsymbol{u}^\ell) \\ \vdots \\ \boldsymbol{u}^0 + \Delta t \sum_{\ell=0}^{M} \theta_\ell^m \boldsymbol{G}(t^\ell, \boldsymbol{u}^\ell) \\ \vdots \\ \boldsymbol{u}^0 + \Delta t \sum_{\ell=0}^{M} \theta_\ell^M \boldsymbol{G}(t^\ell, \boldsymbol{u}^\ell) \end{pmatrix} \text{ with } \underline{\boldsymbol{y}} = \begin{pmatrix} \boldsymbol{y}^1 \\ \vdots \\ \boldsymbol{y}^m \\ \vdots \\ \boldsymbol{y}^M \end{pmatrix}. \tag{16}$$

Again, we remark that $\boldsymbol{u}^0$, the vector corresponding to the initial subtimenode, is always fixed. The proof consists of two parts. We will first show that, for Δt small enough, $\mathcal{J}$ is a contraction over $\mathbb{R}^{(M \times Q)}$, which is a finite dimensional space (and so complete with respect to the distance induced by any norm). This will ensure, thanks to the Banach fixed-point theorem, that there exists a fixed point $\underline{\tilde{\boldsymbol{u}}}$ such that $\underline{\tilde{\boldsymbol{u}}} = \mathcal{J}(\underline{\tilde{\boldsymbol{u}}})$ and that it is unique. It is very easy to see that this fixed point is the (unique) solution to the operator $\mathcal{L}^2_\Delta$. Then, by iteratively applying the operator, we will generate a sequence of vectors converging to this fixed point and we will show that this limit is an $(M+1)$-th order accurate approximation of the exact solution to the system of ODEs.

Let us first prove that $\mathcal{J}$ is a contraction for Δt small enough. We recall that θ_ℓ^m are constant coefficients independent on Δt and bounded by $C_\theta = \max |\theta_\ell^m|$ and that $\boldsymbol{G}(t, \boldsymbol{u})$ is Lipschitz-continuous with respect to $\boldsymbol{u}$ uniformly with respect to t with constant L. Now, using the triangular

inequality, we have

$$
\begin{aligned}
\|\mathcal{J}(\underline{\boldsymbol{v}})-\mathcal{J}(\underline{\boldsymbol{w}})\|_\infty =&\Delta t \left\| \sum_{\ell=0}^{M} \begin{pmatrix} \theta^1_\ell \left[\boldsymbol{G}(t^\ell,\boldsymbol{v}^\ell)-\boldsymbol{G}(t^\ell,\boldsymbol{w}^\ell)\right] \\ \vdots \\ \theta^m_\ell \left[\boldsymbol{G}(t^\ell,\boldsymbol{v}^\ell)-\boldsymbol{G}(t^\ell,\boldsymbol{w}^\ell)\right] \\ \vdots \\ \theta^M_\ell \left[\boldsymbol{G}(t^\ell,\boldsymbol{v}^\ell)-\boldsymbol{G}(t^\ell,\boldsymbol{w}^\ell)\right] \end{pmatrix} \right\|_\infty \\
\leq& \Delta t C_\theta \sum_{\ell=0}^{M} \left\|\boldsymbol{G}(t^\ell,\boldsymbol{v}^\ell)-\boldsymbol{G}(t^\ell,\boldsymbol{w}^\ell)\right\|_{\infty,Q} \\
\leq& \Delta t C_\theta \sum_{\ell=0}^{M} L \left\|\boldsymbol{v}^\ell-\boldsymbol{w}^\ell\right\|_{\infty,Q} \leq \Delta t C_\theta L M \|\underline{\boldsymbol{v}}-\underline{\boldsymbol{w}}\|_\infty .
\end{aligned}
\tag{17}
$$

The last inequality follows from the fact that $\underline{\boldsymbol{v}}-\underline{\boldsymbol{w}}$ contains as components all the vectors $\boldsymbol{v}^\ell-\boldsymbol{w}^\ell$ for all $\ell=1,\dots,M$ and from the fact that $\boldsymbol{v}^0=\boldsymbol{w}^0=\boldsymbol{u}^0$ and so

$$
\left\|\boldsymbol{v}^\ell-\boldsymbol{w}^\ell\right\|_{\infty,Q} \leq \|\underline{\boldsymbol{v}}-\underline{\boldsymbol{w}}\|_\infty, \quad \forall \ell=1,\dots,M, \tag{18}
$$

where $\|\cdot\|_{\infty,Q}$ is the infinity norm over $\mathbb{R}^Q$, while $\|\cdot\|_\infty$ is the infinity norm over $\mathbb{R}^{M\times Q}$. For $\Delta t < \frac{1}{C_\theta L M}$, we have

$$
\|\mathcal{J}(\underline{\boldsymbol{v}})-\mathcal{J}(\underline{\boldsymbol{w}})\|_\infty < \delta \|\underline{\boldsymbol{v}}-\underline{\boldsymbol{w}}\|_\infty \tag{19}
$$

with $\delta<1$ and so $\mathcal{J}$ is a contraction. As anticipated, there exists a unique fixed point $\underline{\tilde{\boldsymbol{u}}}$, solution of $\mathcal{L}^2_\Delta$.

For the second part, we will prove the accuracy of the iteration of the fixed point procedure. We consider the sequence $\{\underline{\boldsymbol{y}}^{(k)}\}_{k\in\mathbb{N}}$ given by the following recursive definition

$$
\underline{\boldsymbol{y}}^{(k)} = \mathcal{J}(\underline{\boldsymbol{y}}^{(k-1)}) \tag{20}
$$

with its general element being

$$
\underline{\boldsymbol{y}}^{(k)} = \begin{pmatrix} \boldsymbol{y}^{1,(k)} \\ \vdots \\ \boldsymbol{y}^{m,(k)} \\ \vdots \\ \boldsymbol{y}^{M,(k)} \end{pmatrix}, \text{ with } \underline{\boldsymbol{y}}^{(0)} = \begin{pmatrix} \boldsymbol{y}^{1,(0)} \\ \vdots \\ \boldsymbol{y}^{m,(0)} \\ \vdots \\ \boldsymbol{y}^{M,(0)} \end{pmatrix} = \begin{pmatrix} \boldsymbol{u}^0 \\ \vdots \\ \boldsymbol{u}^0 \\ \vdots \\ \boldsymbol{u}^0 \end{pmatrix}. \tag{21}
$$

The general component $\boldsymbol{y}^{m,(k)}$ of $\underline{\boldsymbol{y}}^{(k)}$ is a Q-dimensional vector. The first index m is referred to the subtimenode, the second is the index of the sequence. In order to have a more compact notation, we will not write $\boldsymbol{G}(t^0,\boldsymbol{u}^0)$ as a separate term but we set $\boldsymbol{y}^{0,(k)}=\boldsymbol{u}^0\ \forall k\geq 0$, because the value of the solution at the first subtimenode is known. From theory, we know that this sequence converges to the fixed point of $\mathcal{J}$ and so to the solution of the operator $\mathcal{L}^2_\Delta$.

Let us prove by induction on k that for all $m=1,\dots,M$, we have

$$
\boldsymbol{y}^{m,(k)} = \boldsymbol{u}(t^m) + O(\Delta t^{\min(k+1,M+2)}). \tag{22}
$$

The base case, for $k=0$, is clearly true as a simple Taylor expansion gives

$$\boldsymbol{u}(t^m) = \boldsymbol{u}(t^0) + \Delta t \boldsymbol{G}(t^0, \boldsymbol{u}(t^0))(t^m - t^0) + O(\Delta t^2) = \boldsymbol{y}^{m,(0)} + O(\Delta t), \tag{23}$$

reminding that $\frac{d}{dt}\boldsymbol{u}(t) = \boldsymbol{G}(t, \boldsymbol{u}(t))$.

For the induction step, we assume that $\boldsymbol{y}^{m,(k)} = \boldsymbol{u}(t^m) + O(\Delta t^{\min(k+1,M+2)})$ and we will prove that $\boldsymbol{y}^{m,(k+1)} = \boldsymbol{u}(t^m) + O(\Delta t^{\min(k+2,M+2)})$. By exploiting the Lipschitz-continuity of $\boldsymbol{G}$, we have that

$$\begin{aligned}\boldsymbol{G}(t^\ell, \boldsymbol{u}(t^\ell)) &= \boldsymbol{G}(t^\ell, \boldsymbol{y}^{\ell,(k)}) + \nabla_{\boldsymbol{u}} \boldsymbol{G}(t^\ell, \boldsymbol{y}^{\ell,(k)})(\boldsymbol{u}(t^\ell) - \boldsymbol{y}^{\ell,(k)}) + O\left(\left\| \boldsymbol{u}(t^\ell) - \boldsymbol{y}^{\ell,(k)} \right\|^2_{\infty,Q} \right) \\ &= \boldsymbol{G}(t^\ell, \boldsymbol{y}^{\ell,(k)}) + O(\Delta t^{\min(k+1,M+2)}),\end{aligned} \tag{24}$$

where $\nabla_{\boldsymbol{u}} \boldsymbol{G}(t^\ell, \boldsymbol{y}^{\ell,(k)})$ is bounded in some norm by L. We are then able to prove that

$$\begin{aligned}\boldsymbol{y}^{m,(k+1)} &= \boldsymbol{u}(t^0) + \Delta t \sum_{\ell=0}^{M} \theta_\ell^m \boldsymbol{G}(t^\ell, \boldsymbol{y}^{\ell,(k)}) \\ &= \boldsymbol{u}(t^0) + \Delta t \sum_{\ell=0}^{M} \theta_\ell^m \boldsymbol{G}(t^\ell, \boldsymbol{u}(t^\ell)) + O(\Delta t^{1+\min(k+1,M+2)}).\end{aligned} \tag{25}$$

Now, thanks to the $(M+1)$-th order accuracy of (12), we have that

$$\begin{aligned}\boldsymbol{y}^{m,(k+1)} &= \boldsymbol{u}(t^0) + \Delta t \sum_{\ell=0}^{M} \theta_\ell^m \boldsymbol{G}(t^\ell, \boldsymbol{u}(t^\ell)) + O(\Delta t^{1+\min(k+1,M+2)}) \\ &= \boldsymbol{u}(t^m) + O(\Delta t^{M+2}) + O(\Delta t^{1+\min(k+1,M+2)}) = \boldsymbol{u}(t^m) + O(\Delta t^{\min(k+2,M+2)}).\end{aligned} \tag{26}$$

Hence, for $k > M$ the components $\boldsymbol{y}^{(k),m}$ are an $(M+1)$ accurate solution of $u(t^m)$ and their limit for $k \to \infty$, i.e., the solutions of $\mathcal{L}^2_\Delta$, is as well an $(M+1)$ approximation of the exact solution. □

2.1.2 Definition of $\mathcal{L}^1_\Delta$

If we apply the Euler method to get the approximate solution $\boldsymbol{u}^m$ in the node t^m we have

$$\boldsymbol{u}^m - \boldsymbol{u}^0 - \Delta t \beta^m \boldsymbol{G}(t^0, \boldsymbol{u}^0) = \boldsymbol{0}, \tag{27}$$

where $\beta^m = \frac{t^m - t^0}{\Delta t}$.

Proposition 2.3. *Let $\boldsymbol{u}^m$ be the solution of (27), then $\boldsymbol{u}^m$ is first order accurate, i.e., $\boldsymbol{u}(t^m) - \boldsymbol{u}^m = O(\Delta t^2)$.*

Proof. We consider the difference between the exact solution $\boldsymbol{u}(t^m)$ to our ODEs system and $\boldsymbol{u}^m$ got from (27). Through a first order Taylor expansion of $\boldsymbol{u}(t)$ and from the fact that $\frac{d}{dt}\boldsymbol{u}(t) = \boldsymbol{G}(t, \boldsymbol{u}(t))$, we have

$$\boldsymbol{u}(t^m) - \boldsymbol{u}^m = \boldsymbol{u}^0 + \boldsymbol{G}(t^0, \boldsymbol{u}^0)(t^m - t^0) + O(\Delta t^2) - \boldsymbol{u}^0 - \Delta t \beta^m \boldsymbol{G}(t^0, \boldsymbol{u}^0) = O(\Delta t^2), \tag{28}$$

because $\boldsymbol{u}^0 = \boldsymbol{u}(t^0) = \boldsymbol{u}(t_n) = \boldsymbol{u}_n$ and $\beta^m = \frac{t^m - t^0}{\Delta t}$. □

Directly from (27), we get our explicit, low order operator $\mathcal{L}^1_\Delta : \mathbb{R}^{(M\times Q)} \to \mathbb{R}^{(M\times Q)}$ defined as

$$\mathcal{L}^1_\Delta(\underline{\boldsymbol{u}}) = \begin{pmatrix} \boldsymbol{u}^1 - \boldsymbol{u}^0 - \Delta t \beta^1 \boldsymbol{G}(t^0, \boldsymbol{u}^0) \\ \vdots \\ \boldsymbol{u}^m - \boldsymbol{u}^0 - \Delta t \beta^m \boldsymbol{G}(t^0, \boldsymbol{u}^0) \\ \vdots \\ \boldsymbol{u}^M - \boldsymbol{u}^0 - \Delta t \beta^M \boldsymbol{G}(t^0, \boldsymbol{u}^0) \end{pmatrix} \quad \text{with } \underline{\boldsymbol{u}} = \begin{pmatrix} \boldsymbol{u}^1 \\ \vdots \\ \boldsymbol{u}^m \\ \vdots \\ \boldsymbol{u}^M \end{pmatrix}. \tag{29}$$

2.1.3 Proof of the properties of $\mathcal{L}^1_\Delta$ and $\mathcal{L}^2_\Delta$

We equip $X = Y = \mathbb{R}^{(M\times Q)}$ with the infinity norm $\|\cdot\|_\infty$ and we recall here the hypotheses that are needed to apply the Deferred Correction method from the abstract formulation but characterizing them to our case.

i) **Existence of a solution to $\mathcal{L}^2_\Delta$**
$\exists ! \underline{\boldsymbol{u}}_\Delta \in \mathbb{R}^{(M\times Q)}$ solution of $\mathcal{L}^2_\Delta$, i.e. such that $\mathcal{L}^2_\Delta(\underline{\boldsymbol{u}}_\Delta) = \boldsymbol{0}$;

ii) **Coercivity-like property of $\mathcal{L}^1_\Delta$**
$\exists \alpha_1 \geq 0$ independent of Δt s.t.

$$\left\|\mathcal{L}^1_\Delta(\underline{\boldsymbol{v}}) - \mathcal{L}^1_\Delta(\underline{\boldsymbol{w}})\right\|_\infty \geq \alpha_1 \left\|\underline{\boldsymbol{v}} - \underline{\boldsymbol{w}}\right\|_\infty, \quad \forall \underline{\boldsymbol{v}}, \underline{\boldsymbol{w}} \in \mathbb{R}^{(M\times Q)}; \tag{30}$$

iii) **Lipschitz-continuity-like condition of $\mathcal{L}^1_\Delta - \mathcal{L}^2_\Delta$**
$\exists \alpha_2 \geq 0$ independent of Δt s.t.

$$\left\|\left[\mathcal{L}^1_\Delta(\underline{\boldsymbol{v}}) - \mathcal{L}^2_\Delta(\underline{\boldsymbol{v}})\right] - \left[\mathcal{L}^1_\Delta(\underline{\boldsymbol{w}}) - \mathcal{L}^2_\Delta(\underline{\boldsymbol{w}})\right]\right\|_\infty \leq \alpha_2 \Delta t \left\|\underline{\boldsymbol{v}} - \underline{\boldsymbol{w}}\right\|_\infty, \quad \forall \underline{\boldsymbol{v}}, \underline{\boldsymbol{w}} \in \mathbb{R}^{(M\times Q)}. \tag{31}$$

Proof. We prove in order the three properties.

i) **Existence of a solution to $\mathcal{L}^2_\Delta$**
The first property, i.e., the existence of a unique solution to $\mathcal{L}^2_\Delta$, has already been shown in the proof of its $(M+1)$-th order accuracy by introducing the operator $\mathcal{J} : \mathbb{R}^{(M\times Q)} \to \mathbb{R}^{(M\times Q)}$ defined by (16). We showed that for Δt small enough it is a contraction over the space $\mathbb{R}^{(M\times Q)}$ equipped with the infinity norm, so, there exists a unique fixed point of $\mathcal{J}$, which is the unique solution to $\mathcal{L}^2_\Delta$.

ii) **Coercivity-like property of $\mathcal{L}^1_\Delta$**
Let us now consider two generic vectors $\underline{\boldsymbol{v}}, \underline{\boldsymbol{w}} \in \mathbb{R}^{(M\times Q)}$

$$\underline{\boldsymbol{v}} = \begin{pmatrix} \boldsymbol{v}^1 \\ \vdots \\ \boldsymbol{v}^m \\ \vdots \\ \boldsymbol{v}^M \end{pmatrix}, \quad \underline{\boldsymbol{w}} = \begin{pmatrix} \boldsymbol{w}^1 \\ \vdots \\ \boldsymbol{w}^m \\ \vdots \\ \boldsymbol{w}^M \end{pmatrix}, \tag{32}$$

with $\boldsymbol{v}^m$ and $\boldsymbol{w}^m$ for $m = 1, \ldots, M$ generic Q-dimensional vectors. From a direct computation, we have

$$
\begin{aligned}
&\mathcal{L}^1_\Delta(\underline{\boldsymbol{v}}) - \mathcal{L}^1_\Delta(\underline{\boldsymbol{w}}) \\
&= \begin{pmatrix} \boldsymbol{v}^1 - \boldsymbol{u}^0 - \Delta t \beta^1 \boldsymbol{G}(t^0, \boldsymbol{u}^0) \\ \vdots \\ \boldsymbol{v}^m - \boldsymbol{u}^0 - \Delta t \beta^m \boldsymbol{G}(t^0, \boldsymbol{u}^0) \\ \vdots \\ \boldsymbol{v}^M - \boldsymbol{u}^0 - \Delta t \beta^M \boldsymbol{G}(t^0, \boldsymbol{u}^0) \end{pmatrix} - \begin{pmatrix} \boldsymbol{w}^1 - \boldsymbol{u}^0 - \Delta t \beta^1 \boldsymbol{G}(t^0, \boldsymbol{u}^0) \\ \vdots \\ \boldsymbol{w}^m - \boldsymbol{u}^0 - \Delta t \beta^m \boldsymbol{G}(t^0, \boldsymbol{u}^0) \\ \vdots \\ \boldsymbol{w}^M - \boldsymbol{u}^0 - \Delta t \beta^M \boldsymbol{G}(t^0, \boldsymbol{u}^0) \end{pmatrix} = \begin{pmatrix} \boldsymbol{v}^1 - \boldsymbol{w}^1 \\ \vdots \\ \boldsymbol{v}^m - \boldsymbol{w}^m \\ \vdots \\ \boldsymbol{v}^M - \boldsymbol{w}^M \end{pmatrix},
\end{aligned}
\tag{33}
$$

i.e., $\mathcal{L}^1_\Delta(\underline{\boldsymbol{v}}) - \mathcal{L}^1_\Delta(\underline{\boldsymbol{w}}) = \underline{\boldsymbol{v}} - \underline{\boldsymbol{w}}$. Then,

$$
\left\| \mathcal{L}^1_\Delta(\underline{\boldsymbol{v}}) - \mathcal{L}^1_\Delta(\underline{\boldsymbol{w}}) \right\|_\infty = \left\| \underline{\boldsymbol{v}} - \underline{\boldsymbol{w}} \right\|_\infty \tag{34}
$$

and thus the coercivity-like property of $\mathcal{L}^1_\Delta$ is verified with $\alpha_1 = 1$ and results in an equality. Again, we remark that $\boldsymbol{u}^0$ is given, it is part of the problem and embedded in the operators $\mathcal{L}^1_\Delta$ and $\mathcal{L}^2_\Delta$.

iii) **Lipschitz-continuity-like condition of $\mathcal{L}^1_\Delta - \mathcal{L}^2_\Delta$**
Again, we consider a direct computation but focusing, for the sake of compactness, on the Q-dimensional component got for a general m

$$
\begin{aligned}
&\left[\mathcal{L}^{1,m}_\Delta(\underline{\boldsymbol{v}}) - \mathcal{L}^{2,m}_\Delta(\underline{\boldsymbol{v}}) \right] - \left[\mathcal{L}^{1,m}_\Delta(\underline{\boldsymbol{w}}) - \mathcal{L}^{2,m}_\Delta(\underline{\boldsymbol{w}}) \right] \\
=& \boldsymbol{v}^m - \boldsymbol{u}^0 - \Delta t \beta^m \boldsymbol{G}(t^0, \boldsymbol{u}^0) - \boldsymbol{v}^m + \boldsymbol{u}^0 + \Delta t \sum_{\ell=0}^{M} \theta^m_\ell \boldsymbol{G}(t^\ell, \boldsymbol{v}^\ell) \\
&- \left[\boldsymbol{w}^m - \boldsymbol{u}^0 - \Delta t \beta^m \boldsymbol{G}(t^0, \boldsymbol{u}^0) - \boldsymbol{w}^m + \boldsymbol{u}^0 + \Delta t \sum_{\ell=0}^{M} \theta^m_\ell \boldsymbol{G}(t^\ell, \boldsymbol{w}^\ell) \right] \\
=& \Delta t \sum_{\ell=0}^{M} \theta^m_\ell \left(\boldsymbol{G}(t^\ell, \boldsymbol{v}^\ell) - \boldsymbol{G}(t^\ell, \boldsymbol{w}^\ell) \right),
\end{aligned}
\tag{35}
$$

where clearly $\boldsymbol{v}^0 = \boldsymbol{w}^0 = \boldsymbol{u}^0$. As we pointed out several times, $\boldsymbol{u}^0$ is not an unknown, it is a given vector, it is "part" of the problem and is embedded in the operators. We use $\boldsymbol{v}^0$ and $\boldsymbol{w}^0$ instead of $\boldsymbol{u}^0$ for the sake of compactness. Let us recall that θ^m_ℓ, for $m = 1, \ldots, M$ and $\ell = 0, 1, \ldots, M$, are fixed constant coefficients independent of Δt, thus bounded in absolute value by a positive constant C_θ, and that $\boldsymbol{G}(t, \boldsymbol{u})$ is Lipschitz-continuous with respect to $\boldsymbol{u}$ uniformly with respect to t with a Lipschitz constant L. By applying the triangular inequality,

we have

$$
\begin{aligned}
&\left\| \left[\mathcal{L}^1_\Delta(\underline{\boldsymbol{v}}) - \mathcal{L}^2_\Delta(\underline{\boldsymbol{v}})\right] - \left[\mathcal{L}^1_\Delta(\underline{\boldsymbol{w}}) - \mathcal{L}^2_\Delta(\underline{\boldsymbol{w}})\right] \right\|_\infty \\
=&\Delta t \left\| \sum_{\ell=0}^M \begin{pmatrix} \theta^1_\ell \left[\boldsymbol{G}(t^\ell,\boldsymbol{v}^\ell) - \boldsymbol{G}(t^\ell,\boldsymbol{w}^\ell)\right] \\ \vdots \\ \theta^m_\ell \left[\boldsymbol{G}(t^\ell,\boldsymbol{v}^\ell) - \boldsymbol{G}(t^\ell,\boldsymbol{w}^\ell)\right] \\ \vdots \\ \theta^M_\ell \left[\boldsymbol{G}(t^\ell,\boldsymbol{v}^\ell) - \boldsymbol{G}(t^\ell,\boldsymbol{w}^\ell)\right] \end{pmatrix} \right\|_\infty \leq \Delta t C_\theta \sum_{\ell=0}^M \left\| \begin{pmatrix} \boldsymbol{G}(t^\ell,\boldsymbol{v}^\ell) - \boldsymbol{G}(t^\ell,\boldsymbol{w}^\ell) \\ \vdots \\ \boldsymbol{G}(t^\ell,\boldsymbol{v}^\ell) - \boldsymbol{G}(t^\ell,\boldsymbol{w}^\ell) \\ \vdots \\ \boldsymbol{G}(t^\ell,\boldsymbol{v}^\ell) - \boldsymbol{G}(t^\ell,\boldsymbol{w}^\ell) \end{pmatrix} \right\|_\infty \\
=&\Delta t C_\theta \sum_{\ell=0}^M \left\| \boldsymbol{G}(t^\ell,\boldsymbol{v}^\ell) - \boldsymbol{G}(t^\ell,\boldsymbol{w}^\ell) \right\|_{\infty,Q} \leq \Delta t C_\theta \sum_{\ell=0}^M L \left\| \boldsymbol{v}^\ell - \boldsymbol{w}^\ell \right\|_{\infty,Q} \leq \Delta t C_\theta L M \left\| \underline{\boldsymbol{v}} - \underline{\boldsymbol{w}} \right\|_\infty ,
\end{aligned}
\tag{36}
$$

where the last inequality follows from the fact that $\underline{\boldsymbol{v}} - \underline{\boldsymbol{w}}$ contains as components all the vectors $\boldsymbol{v}^\ell - \boldsymbol{w}^\ell$ for $\ell = 1, \dots, M$ and from the fact that $\boldsymbol{v}^0 = \boldsymbol{w}^0 = \boldsymbol{u}^0$. This proves the Lipschitz-continuity-like condition of $\mathcal{L}^1_\Delta - \mathcal{L}^2_\Delta$ with $\alpha_2 = C_\theta L M$. For more clarity, we underline that the infinity norm $\|\cdot\|_{\infty,Q}$ is applied to Q-dimensional vectors (and not to $(M \times Q)$-dimensional vectors like $\|\cdot\|_\infty$). This completes the analysis of the Deferred Correction applied to the context of the systems of ordinary differential equations.

□

Remark 2.1 (On the optimal value of α_2)**.** *The constant $C_\theta L M$ is not the sharpest estimate for α_2 in* (31)*. Introducing the support structures*

$$
\Theta = \begin{pmatrix} 0 & 0 & \dots & 0 \\ \theta^1_0 & \theta^1_1 & \dots & \theta^1_M \\ \theta^2_0 & \theta^2_1 & \dots & \theta^2_M \\ \vdots & \vdots & \ddots & \vdots \\ \theta^M_0 & \theta^M_1 & \dots & \theta^M_M \end{pmatrix}, \quad \underline{\boldsymbol{G}}(\underline{\boldsymbol{v}}) = \begin{pmatrix} \boldsymbol{G}(t^1,\boldsymbol{v}^1) \\ \vdots \\ \boldsymbol{G}(t^m,\boldsymbol{v}^m) \\ \vdots \\ \boldsymbol{G}(t^M,\boldsymbol{v}^M) \end{pmatrix}, \quad \underline{\boldsymbol{G}}(\underline{\boldsymbol{w}}) = \begin{pmatrix} \boldsymbol{G}(t^1,\boldsymbol{w}^1) \\ \vdots \\ \boldsymbol{G}(t^m,\boldsymbol{w}^m) \\ \vdots \\ \boldsymbol{G}(t^M,\boldsymbol{w}^M) \end{pmatrix}, \tag{37}
$$

and recalling that $\boldsymbol{v}^0 = \boldsymbol{w}^0 = \boldsymbol{u}^0$, one can easily verify that

$$
\sum_{\ell=0}^M \begin{pmatrix} \theta^1_\ell \left[\boldsymbol{G}(t^\ell,\boldsymbol{v}^\ell) - \boldsymbol{G}(t^\ell,\boldsymbol{w}^\ell)\right] \\ \vdots \\ \theta^m_\ell \left[\boldsymbol{G}(t^\ell,\boldsymbol{v}^\ell) - \boldsymbol{G}(t^\ell,\boldsymbol{w}^\ell)\right] \\ \vdots \\ \theta^M_\ell \left[\boldsymbol{G}(t^\ell,\boldsymbol{v}^\ell) - \boldsymbol{G}(t^\ell,\boldsymbol{w}^\ell)\right] \end{pmatrix} = \Theta_{1:,1:} \left[\underline{\boldsymbol{G}}(\underline{\boldsymbol{v}}) - \underline{\boldsymbol{G}}(\underline{\boldsymbol{w}})\right], \tag{38}
$$

where by $\Theta_{1:,1:}$ we mean the submatrix extracted from Θ with row and column indices from 1 *on, assuming a zero-based numeration.*

Therefore, we have that

$$\left\| \sum_{\ell=0}^{M} \begin{pmatrix} \theta_\ell^1 \left[\boldsymbol{G}(t^\ell, \boldsymbol{v}^\ell) - \boldsymbol{G}(t^\ell, \boldsymbol{w}^\ell) \right] \\ \vdots \\ \theta_\ell^m \left[\boldsymbol{G}(t^\ell, \boldsymbol{v}^\ell) - \boldsymbol{G}(t^\ell, \boldsymbol{w}^\ell) \right] \\ \vdots \\ \theta_\ell^M \left[\boldsymbol{G}(t^\ell, \boldsymbol{v}^\ell) - \boldsymbol{G}(t^\ell, \boldsymbol{w}^\ell) \right] \end{pmatrix} \right\|_\infty = \left\| \Theta_{1:,1:} \left[\underline{\boldsymbol{G}}(\underline{\boldsymbol{v}}) - \underline{\boldsymbol{G}}(\underline{\boldsymbol{w}}) \right] \right\|_\infty , \tag{39}$$

By basic linear algebra and thanks to the Lipschitz-continuity of $\boldsymbol{G}(t, \boldsymbol{u})$ with respect to $\boldsymbol{u}$ uniformly with respect to t, we get

$$\begin{aligned} \left\| \Theta_{1:,1:} \left[\underline{\boldsymbol{G}}(\underline{\boldsymbol{v}}) - \underline{\boldsymbol{G}}(\underline{\boldsymbol{w}}) \right] \right\|_\infty &\leq \left\| \Theta_{1:,1:} \right\|_\infty \left\| \underline{\boldsymbol{G}}(\underline{\boldsymbol{v}}) - \underline{\boldsymbol{G}}(\underline{\boldsymbol{w}}) \right\|_\infty \\ &\leq \left\| \Theta_{1:,1:} \right\|_\infty L \left\| \underline{\boldsymbol{v}} - \underline{\boldsymbol{w}} \right\|_\infty \end{aligned} \tag{40}$$

where $\|\cdot\|_\infty$ applied to the matrix $\Theta_{1:,1:}$ is the matrix norm induced by the corresponding vector norm and hence $\left\| \Theta_{1:,1:} \right\|_\infty = \max\limits_{m=1,\dots,M} \sum\limits_{\ell=1}^{M} |\theta_\ell^m|$. Thus, one gets

$$\left\| \left[\mathcal{L}^1_\Delta(\underline{\boldsymbol{v}}) - \mathcal{L}^2_\Delta(\underline{\boldsymbol{v}}) \right] - \left[\mathcal{L}^1_\Delta(\underline{\boldsymbol{w}}) - \mathcal{L}^2_\Delta(\underline{\boldsymbol{w}}) \right] \right\|_\infty \leq \alpha_2 \Delta t \left\| \underline{\boldsymbol{v}} - \underline{\boldsymbol{w}} \right\|_\infty \tag{41}$$

with $\alpha_2 = \left\| \Theta_{1:,1:} \right\|_\infty L$, which constitutes a sharper estimate with respect to $C_\theta LM$. Indeed, the reported matrix Θ is referred to a scalar problem and it must be block-expanded in the context of a vectorial problem, however, this does not influence the estimate.

2.2 sDeC

The construction of this DeC method makes use of the definition of the subtimenodes introduced for the bDeC method. The main difference is that here we focus on the integration of the system of ODEs in the intervals $[t^{m-1}, t^m]$ rather than $[t^0, t^m]$.

2.2.1 Definition of $\mathcal{L}^2_\Delta$

We start from the exact integration of the system of ODEs in the interval $[t^{m-1}, t^m]$, which would result in

$$\boldsymbol{u}(t^m) - \boldsymbol{u}(t^{m-1}) - \int_{t^{m-1}}^{t^m} \boldsymbol{G}(t, \boldsymbol{u}(t)) dt = \boldsymbol{0}, \quad \forall m = 1, \dots, M. \tag{42}$$

Again, in order to get an expression that can actually be used, we replace $\boldsymbol{G}(t, \boldsymbol{u}(t))$ with its M-th order accurate Lagrange interpolant of degree M associated to the $M+1$ subtimenodes t^m and replace $\boldsymbol{u}(t^\ell)$ by $\boldsymbol{u}^\ell$ thus getting

$$\boldsymbol{u}^m - \boldsymbol{u}^{m-1} - \int_{t^{m-1}}^{t^m} \sum_{\ell=0}^{M} \boldsymbol{G}(t^\ell, \boldsymbol{u}^\ell) \psi^\ell(t) dt = \boldsymbol{0}, \quad \forall m = 1, \dots, M. \tag{43}$$

Moving the finite sum and the vectors $\boldsymbol{G}(t^\ell, \boldsymbol{u}^\ell)$ outside of the integral and performing the exact integration of the Lagrangian polynomial functions $\psi^\ell(t)$ in the subinterval $[t^{m-1}, t^m]$ we get

$$\boldsymbol{u}^m - \boldsymbol{u}^{m-1} - \Delta t \sum_{\ell=0}^{M} \delta_\ell^m \boldsymbol{G}(t^\ell, \boldsymbol{u}^\ell) = \boldsymbol{0}, \quad \forall m = 1, \dots, M, \tag{44}$$

where, just like in the previous case, coefficients δ_ℓ^m are normalized integrals of the Lagrange basis functions independent of Δt.

Our implicit $(M+1)$-th order accurate operator $\mathcal{L}^2_\Delta : \mathbb{R}^{(M\times Q)} \to \mathbb{R}^{(M\times Q)}$ is therefore defined as

$$\mathcal{L}^2_\Delta(\underline{\boldsymbol{u}}) = \begin{pmatrix} \boldsymbol{u}^1 - \boldsymbol{u}^0 - \Delta t \sum_{\ell=0}^M \delta_\ell^1 \boldsymbol{G}(t^\ell, \boldsymbol{u}^\ell) \\ \vdots \\ \boldsymbol{u}^m - \boldsymbol{u}^{m-1} - \Delta t \sum_{\ell=0}^M \delta_\ell^m \boldsymbol{G}(t^\ell, \boldsymbol{u}^\ell) \\ \vdots \\ \boldsymbol{u}^M - \boldsymbol{u}^{M-1} - \Delta t \sum_{\ell=0}^M \delta_\ell^M \boldsymbol{G}(t^\ell, \boldsymbol{u}^\ell) \end{pmatrix} \text{ with } \underline{\boldsymbol{u}} = \begin{pmatrix} \boldsymbol{u}^1 \\ \vdots \\ \boldsymbol{u}^m \\ \vdots \\ \boldsymbol{u}^M \end{pmatrix}. \tag{45}$$

2.2.2 Definition of $\mathcal{L}^1_\Delta$

Also in this case the operator $\mathcal{L}^1_\Delta$ is obtained by a first order approximation in the integration of our initial system of ODEs. Applying the Euler method in the subinterval $[t^{m-1}, t^m]$, we get

$$\boldsymbol{u}^m - \boldsymbol{u}^{m-1} - \Delta t \gamma^m \boldsymbol{G}(t^{m-1}, \boldsymbol{u}^{m-1}) = \boldsymbol{0} \tag{46}$$

where $\gamma^m = \frac{t^m - t^{m-1}}{\Delta t}$ are normalized coefficients. The explicit, first order order operator $\mathcal{L}^1_\Delta : \mathbb{R}^{(M\times Q)} \to \mathbb{R}^{(M\times Q)}$ is defined as

$$\mathcal{L}^1_\Delta(\underline{\boldsymbol{u}}) = \begin{pmatrix} \boldsymbol{u}^1 - \boldsymbol{u}^0 - \Delta t \gamma^1 \boldsymbol{G}(t^0, \boldsymbol{u}^0) \\ \vdots \\ \boldsymbol{u}^m - \boldsymbol{u}^{m-1} - \Delta t \gamma^m \boldsymbol{G}(t^{m-1}, \boldsymbol{u}^{m-1}) \\ \vdots \\ \boldsymbol{u}^M - \boldsymbol{u}^{M-1} \Delta t \gamma^M \boldsymbol{G}(t^{M-1}, \boldsymbol{u}^{M-1}) \end{pmatrix} \text{ with } \underline{\boldsymbol{u}} = \begin{pmatrix} \boldsymbol{u}^1 \\ \vdots \\ \boldsymbol{u}^m \\ \vdots \\ \boldsymbol{u}^M \end{pmatrix}. \tag{47}$$

2.2.3 sDeC as a perturbation of bDeC

The proofs seen for the previous formulation cannot be extended to this case in a straightforward way, but it is possible to show that the second formulation is actually a perturbation of the first one with no impact on the accuracy. Let us recall here, for more clarity, the updating formulas of the bDeC and of the sDeC methods for the computation of $\boldsymbol{u}^{m,(p)}$, m-th component of the approximated solution at the iteration p,

- **bDeC**

$$\boldsymbol{u}_b^{m,(p)} = \boldsymbol{u}^0 + \Delta t \sum_{\ell=0}^M \theta_\ell^m \boldsymbol{G}(t^\ell, \boldsymbol{u}_b^{\ell,(p-1)}) \tag{48}$$

- **sDeC**

$$\boldsymbol{u}_s^{m,(p)} = \boldsymbol{u}^0 + \Delta t \sum_{\ell=0}^{m-1} \gamma^{\ell+1} \left(\boldsymbol{G}(t^\ell, \boldsymbol{u}_s^{\ell,(p)}) - \boldsymbol{G}(t^\ell, \boldsymbol{u}_s^{\ell,(p-1)}) \right) + \Delta t \sum_{\ell=0}^M \theta_\ell^m \boldsymbol{G}(t^\ell, \boldsymbol{u}_s^{\ell,(p-1)}). \tag{49}$$

The difference lies in the term

$$\Delta t \sum_{\ell=0}^{m-1} \gamma^\ell \left(\boldsymbol{G}(t^\ell, \boldsymbol{u}_s^{\ell,(p)}) - \boldsymbol{G}(t^\ell, \boldsymbol{u}_s^{\ell,(p-1)}) \right), \tag{50}$$

which consists in a sum of differences of evaluations of the function $\boldsymbol{G}$ multiplied by Δt. We will show now why this term can be seen as a perturbation of the updating formula of the first formulation with no impact on the accuracy. This actually depends on the fact that $\boldsymbol{u}^{\ell,(p)}$ and $\boldsymbol{u}^{\ell,(p-1)}$ are approximations of the same quantity.

Proposition 2.4 (sDeC accuracy)**.** *The approximation $\boldsymbol{u}_s^{m,(p)}$ provided by the sDeC (49) is an $O(\Delta t^{p+1})$ perturbation of $\boldsymbol{u}_b^{m,(p)}$ obtained through the bDeC (48).*

Proof. We will prove it by induction over p and m. The base case of the induction is clearly true as $\boldsymbol{u}_s^{m,(p)} = \boldsymbol{u}_b^{m,(p)} = \boldsymbol{u}^0$ whenever p or m are equal to 0. We focus now on the induction step. We select $p, m \geq 1$ and assume

$$\boldsymbol{u}_s^{\ell,(k)} = \boldsymbol{u}_b^{\ell,(k)} + O(\Delta t^{k+1}), \text{ for } \begin{cases} k < p, & \forall \ell = 1, \dots, M, \text{ or} \\ k = p, & \forall \ell \leq m-1 \end{cases} \tag{51}$$

and we will prove that $\boldsymbol{u}_s^{m,(p)} = \boldsymbol{u}_b^{m,(p)} + O(\Delta t^{p+1})$. We start from (49) and, thanks to the induction hypothesis, to the Lipschitz-continuity of $\boldsymbol{G}$ and by definition of $\boldsymbol{u}_b^{m,(p)}$ in (48), we have that

$$\begin{aligned}
\boldsymbol{u}_s^{m,(p)} &= \boldsymbol{u}^0 + \Delta t \sum_{\ell=0}^{m-1} \gamma^{\ell+1} \left(\boldsymbol{G}(t^\ell, \boldsymbol{u}_s^{\ell,(p)}) - \boldsymbol{G}(t^\ell, \boldsymbol{u}_s^{\ell,(p-1)}) \right) + \Delta t \sum_{\ell=0}^{M} \theta_\ell^m \boldsymbol{G}(t^\ell, \boldsymbol{u}_s^{\ell,(p-1)}) \\
&= \boldsymbol{u}^0 + \Delta t \sum_{\ell=0}^{m-1} \gamma^{\ell+1} \left(\boldsymbol{G}(t^\ell, \boldsymbol{u}_b^{\ell,(p)}) - \boldsymbol{G}(t^\ell, \boldsymbol{u}_b^{\ell,(p-1)}) + O(\Delta t^p) \right) \\
&\qquad\qquad + \Delta t \left(\sum_{\ell=0}^{M} \theta_\ell^m \boldsymbol{G}(t^\ell, \boldsymbol{u}_b^{\ell,(p-1)}) + O(\Delta t^p) \right) \\
&= \boldsymbol{u}_b^{m,(p)} + \Delta t \sum_{\ell=0}^{m-1} \gamma^{\ell+1} \left(\boldsymbol{G}(t^\ell, \boldsymbol{u}_b^{\ell,(p)}) - \boldsymbol{G}(t^\ell, \boldsymbol{u}_b^{\ell,(p-1)}) \right) + O(\Delta t^{p+1}).
\end{aligned} \tag{52}$$

Thanks again to the Lipschitz-continuity of $\boldsymbol{G}$ and to the results on the accuracy of the bDeC method, for each $\ell = 1, \dots, m-1$, we can write

$$\begin{aligned}
\left\| \boldsymbol{G}(t^\ell, \boldsymbol{u}_b^{\ell,(p)}) - \boldsymbol{G}(t^\ell, \boldsymbol{u}_b^{\ell,(p-1)}) \right\|_{\infty,Q} &\leq L \left\| \boldsymbol{u}_b^{\ell,(p)} - \boldsymbol{u}_b^{\ell,(p-1)} \right\|_{\infty,Q} \\
&\leq L \left\| \boldsymbol{u}_\Delta^\ell - \boldsymbol{u}_\Delta^\ell + O(\Delta t^p) \right\|_{\infty,Q} = O(\Delta t^p),
\end{aligned} \tag{53}$$

where $\boldsymbol{u}_\Delta^\ell$ is the ℓ-th component of $\underline{\boldsymbol{u}}_\Delta$, solution to $\mathcal{L}_\Delta^2$; further, for $\ell = 0$ we have $\boldsymbol{G}(t^\ell, \boldsymbol{u}_b^{\ell,(p)}) - \boldsymbol{G}(t^\ell, \boldsymbol{u}_b^{\ell,(p-1)}) = \boldsymbol{0}$ as the component at the initial subtimenode is always equal to $\boldsymbol{u}^0$. By the previous fact, coming back to (52), we get the thesis

$$\boldsymbol{u}_s^{m,(p)} = \boldsymbol{u}_b^{m,(p)} + O(\Delta t^{p+1}). \tag{54}$$

□

3 Continuous Galerkin FEM

Let $\Omega \subset \mathbb{R}^D$ an open regular bounded domain. The general form of a hyperbolic system of balance laws reads

$$\frac{\partial}{\partial t}\boldsymbol{u}(\boldsymbol{x},t) + \operatorname{div}_{\boldsymbol{x}}\boldsymbol{F}(\boldsymbol{u}(\boldsymbol{x},t)) = \boldsymbol{S}(\boldsymbol{x},\boldsymbol{u}(\boldsymbol{x},t)), \qquad (\boldsymbol{x},t) \in \Omega \times \mathbb{R}_0^+, \tag{55}$$

provided with some initial condition $\boldsymbol{u}(\boldsymbol{x},0) = \boldsymbol{u}_0(\boldsymbol{x})$ on Ω and some boundary conditions on $\partial\Omega$.

Let us define $\mathcal{T}_h$ a triangulation of $\overline{\Omega}$ and denote with K the general element, which we assume to be convex and closed. Consider the continuous finite element space $V_h = \{g \in C^0(\overline{\Omega}) : g|_K \in \mathbb{P}_M(K)\ \forall K \in \mathcal{T}_h\}$, let $\{\varphi_i\}_{i=1,\dots,I}$ be a basis of V_h such that each φ_i can be associated to a degree of freedom $\boldsymbol{x}_i \in \overline{\Omega}$ and has support contained in $\mathcal{K}_i := \cup_{K\in K_i} K$, where $K_i := \{K \in \mathcal{T}_h : \boldsymbol{x}_i \in K\}$. Further, we assume the basis functions normalized in such a way that $\sum_{i=1}^I \varphi_i \equiv 1$. The general form of the semidiscrete formulation of a continuous Galerkin FEM scheme consists in finding a solution $\boldsymbol{u}_h(\boldsymbol{x}) = \sum_i \boldsymbol{c}_i(t)\varphi_i(\boldsymbol{x})$, with $\boldsymbol{c}_i(t) \in \mathbb{R}^Q$ at any time t, such that

$$\sum_{K\in K_i}\sum_{\boldsymbol{x}_j\in K}\left(\int_K \varphi_i(\boldsymbol{x})\varphi_j(\boldsymbol{x})d\boldsymbol{x}\right)\frac{d}{dt}\boldsymbol{c}_j(t) + \boldsymbol{\phi}_i(\boldsymbol{c}(t)) = \boldsymbol{0}, \qquad \forall i = 1,\dots,I, \tag{56}$$

where $\boldsymbol{ST}_i(\boldsymbol{u}_h)$ are some stabilization terms and the space residuals $\boldsymbol{\phi}_i(\boldsymbol{c}(t))$ are defined as

$$\boldsymbol{\phi}_i(\boldsymbol{c}(t)) = \sum_{K\in K_i}\int_K \left(\operatorname{div}_{\boldsymbol{x}}\boldsymbol{F}(\boldsymbol{u}_h(\boldsymbol{x},t)) - \boldsymbol{S}(\boldsymbol{x},\boldsymbol{u}_h(\boldsymbol{x},t))\right)\varphi_i(\boldsymbol{x})d\boldsymbol{x} + \boldsymbol{ST}_i(\boldsymbol{u}_h), \tag{57}$$

with $\boldsymbol{c}(t) \in \mathbb{R}^{I\times Q}$ containing as components all the Q-dimensional vectors $\boldsymbol{c}_i(t)$ associated to the DoFs.

3.1 DeC for CG

In this context, the parameter Δ of the Deferred Correction is the mesh parameter h of the space discretization. We assume CFL conditions on the temporal step size, i.e., $\Delta t \le Ch$ for some fixed constant $C > 0$. We will implicitly assume the Bernstein polynomials as basis functions; nevertheless, the method can be extended also to other basis functions provided that some constraints concerning the construction of the operator $\mathcal{L}^1_\Delta$, specified in the following, are fulfilled.

3.1.1 Preliminary results

Here, we will present some useful preliminary results that will be used later to prove the first order accuracy of $\mathcal{L}^1_\Delta$ and the Lipschitz-continuity-like condition of $\mathcal{L}^1_\Delta - \mathcal{L}^2_\Delta$. In particular, we will prove two propositions, via some intermediate lemmas. We will focus on the Bernstein polynomials; nevertheless the results can be easily extended to other polynomial bases.

Let us consider a general element K, the vector space $\mathbb{P}_M(K)$ of the scalar polynomial functions of degree M defined on it and $u \in \mathbb{P}_M(K)$. We can express u as a linear combination of the Bernstein polynomials $\{\varphi_r\}_{r=1,\dots,R}$ of degree M defined on the element because they are a basis of $\mathbb{P}_M(K)$. We have thus

$$u(\boldsymbol{x}) = \sum_{r=1}^R c_r\varphi_r(\boldsymbol{x}), \quad \forall \boldsymbol{x} \in K, \tag{58}$$

where the scalar coefficients c_r are the Bernstein coefficients associated to the DoFs $\boldsymbol{x}_r \in K$. Another possibility is to express u in terms of the Lagrange basis functions $\{\hat{\varphi}_r\}_{r=1,\dots,R}$ defined on K which constitute another basis of $\mathbb{P}_M(K)$. Therefore, we can also write

$$u(\boldsymbol{x}) = \sum_{r=1}^{R} v_r \hat{\varphi}_r(\boldsymbol{x}), \quad \forall \boldsymbol{x} \in K, \tag{59}$$

where the scalar coefficients v_r are the values of u in the DoFs $\boldsymbol{x}_r \in K$. We define the vector $\boldsymbol{c} \in \mathbb{R}^R$ of the coefficients of $u \in \mathbb{P}_M(K)$ with respect to the Bernstein basis and the vector $\boldsymbol{v} \in \mathbb{R}^R$ of the values of u in all the DoFs of K, i.e., the coefficients with respect to the Lagrange basis.

It is always possible to pass from the Bernstein coefficients to the values in the DoFs through the transition matrix T defined as

$$T = \begin{pmatrix} \varphi_1(\boldsymbol{x}_1) & \varphi_2(\boldsymbol{x}_1) & \dots & \varphi_R(\boldsymbol{x}_1) \\ \varphi_1(\boldsymbol{x}_2) & \varphi_2(\boldsymbol{x}_2) & \dots & \varphi_R(\boldsymbol{x}_2) \\ \vdots & \vdots & \ddots & \vdots \\ \varphi_1(\boldsymbol{x}_R) & \varphi_2(\boldsymbol{x}_R) & \dots & \varphi_R(\boldsymbol{x}_R) \end{pmatrix}. \tag{60}$$

The general element of $T = (T_{ij})_{i,j=1,\dots,R}$ with row index i and column index j is $T_{ij} = \varphi_j(\boldsymbol{x}_i)$ and we have $\boldsymbol{v} = T\boldsymbol{c}$ and $\boldsymbol{c} = T^{-1}\boldsymbol{v}$.

Remark 3.1 (Independence of the mesh parameter.)**.** *Neither the matrix T nor its inverse T^{-1} depend on the size of the element K. They just depend on the spatial dimension D and on the degree M. Once we fix D and M, for any specific type of elements, for example the simplices, we have a fixed T and T^{-1}.*

It is clear that the sum of the elements of each row of T is equal to 1, in fact

$$\sum_{j=1}^{R} T_{ij} = \sum_{j=1}^{R} \varphi_j(\boldsymbol{x}_i) = 1, \quad \forall i = 1, \dots, R. \tag{61}$$

This is due to the assumption on the basis functions, which are normalized in such a way that that

$$\sum_{j=1}^{R} \varphi_j(\boldsymbol{x}) \equiv 1, \quad \forall \boldsymbol{x} \in K. \tag{62}$$

Also its inverse T^{-1} enjoys the same property as we will prove in the next lemma.

Lemma 3.1. *The sum of the elements of each row of T^{-1}, inverse of the transition matrix defined in* (60)*, is equal to* 1*.*

Proof. Let us observe that proving the thesis is equivalent to prove that $T^{-1}\mathbf{1} = \mathbf{1}$ where $\mathbf{1} \in \mathbb{R}^R$ is a vector with all the entries equal to 1. From (61) we have that $T\mathbf{1} = \mathbf{1}$. Thanks to the previous equality, we have that

$$T^{-1}\mathbf{1} = T^{-1}T\mathbf{1} = \mathbf{1} \tag{63}$$

which is the thesis. □

The previous result will be used to prove the following lemma.

Lemma 3.2. *For any polynomial $u \in \mathbb{P}_M(K)$ such that*

$$u(\boldsymbol{x}) = \sum_{r=1}^{R} c_r \varphi_r(\boldsymbol{x}) = \sum_{r=1}^{R} v_r \hat{\varphi}_r(\boldsymbol{x}), \quad \forall \boldsymbol{x} \in K, \tag{64}$$

where φ_r are the Bernstein polynomials of $\mathbb{P}_M(K)$, c_r the Bernstein coefficients, $\hat{\varphi}_r$ the Lagrange polynomials of $\mathbb{P}_M(K)$ and v_r the Lagrange coefficients, it holds that

$$\sup_{i,j=1,\dots,R} |c_i - c_j| \leq \tilde{C} \sup_{i,j=1,\dots,R} |v_i - v_j|, \tag{65}$$

where $\tilde{C} > 0$ is independent of the size and aspect ratio of K.

Proof. The proof is a straightforward consequence of lemma 3.1. From the fact that $\boldsymbol{c} = T^{-1}\boldsymbol{v}$ we know that every Bernstein coefficient c_r can be expressed as a linear combination of the values v_k in the DoFs through the coefficients of the row r of the matrix T^{-1}

$$c_i = \sum_{k=1}^{R} (T^{-1})_{ik} v_k, \quad c_j = \sum_{k=1}^{R} (T^{-1})_{jk} v_k \tag{66}$$

and therefore

$$|c_i - c_j| = \left| \sum_{k=1}^{R} (T^{-1})_{ik} v_k - \sum_{k=1}^{R} (T^{-1})_{jk} v_k \right|. \tag{67}$$

Now, from lemma 3.1, we know that the coefficients $(T^{-1})_{rk}$ are such that

$$\sum_{k=1}^{R} (T^{-1})_{rk} = 1 \quad \forall r = 1, \dots, R. \tag{68}$$

This is in particular true for $r = i$ and $r = j$ and so there exist some coefficients $\lambda_{k,\ell}^{i,j}$, depending on i and j, such that (67) can be written as

$$|c_i - c_j| = \left| \sum_{k=1}^{R} (T^{-1})_{ik} v_k - \sum_{k=1}^{R} (T^{-1})_{jk} v_k \right| = \left| \sum_{k,\ell=1}^{R} \lambda_{k,\ell}^{i,j} (v_k - v_\ell) \right|. \tag{69}$$

One simple choice of these coefficients is given by $\lambda_{k,\ell}^{i,j} = \frac{(T^{-1})_{ik} - (T^{-1})_{jk}}{R}$ and a simple computation can be used to prove it. This might lead to suboptimal values of the estimations. The coefficients $\lambda_{k,\ell}^{i,j}$, like the coefficients T_{ij} and $(T^{-1})_{ij}$, do not depend on the size of K, and, thus, they can be bounded by a positive constant C_λ, which depends just on the type of the element considered. Then, thanks to the triangular inequality, (69) gives

$$|c_i - c_j| = \left| \sum_{k,\ell=1}^{R} \lambda_{k,\ell}^{i,j} (v_k - v_\ell) \right| \leq \sum_{k,\ell=1}^{R} |\lambda_{k,\ell}^{i,j}| |v_k - v_\ell| \leq C_\lambda \sum_{k,\ell=1}^{R} |v_k - v_\ell|. \tag{70}$$

Since the number of dimensions D and the degree M are fixed, also R is fixed and so the number of terms in the sum. Therefore, from (70) we get

$$|c_i - c_j| \leq C_\lambda \sum_{k,\ell=1}^{R} |v_k - v_\ell| \leq \tilde{C} \sup_{i,j=1,\dots,R} |v_i - v_j| \tag{71}$$

for some $\tilde{C} = C_\lambda R^2$ independent of the size of K. □

This allows to prove the following result.

Lemma 3.3. *For any polynomial $u \in \mathbb{P}_M(K)$ such that $u(\boldsymbol{x}) = \sum_{r=1}^{R} c_r \varphi_r(\boldsymbol{x})$, $\forall \boldsymbol{x} \in K$, where φ_r are the Bernstein polynomials of $\mathbb{P}_M(K)$ and c_r the Bernstein coefficients, then*

$$\sup_{i,j=1,\dots,R} |c_i - c_j| \leq \tilde{C} h \left\| \|\nabla_{\boldsymbol{x}} u\|_1 \right\|_{L^\infty(K)} \tag{72}$$

where $\tilde{C}$ is the positive constant in (65) *(and thus independent of the size of K, dependent just on the number of dimensions D, on the degree M and on the type of the element) and h is such that $diam(K) \leq h$. The norm $\|\cdot\|_1$ is the* 1*-norm in $\mathbb{R}^D$, the norm $\|\cdot\|_{L^\infty(K)}$ is the L^∞ norm over K.*

Proof. This is a consequence of lemma 3.2, in fact, from basic analysis, we know that for any smooth scalar function $f \in C^1(K)$

$$\sup_{\boldsymbol{x},\boldsymbol{y} \in K} |f(\boldsymbol{x}) - f(\boldsymbol{y})| \leq h \left\| \|\nabla_{\boldsymbol{x}} f\|_1 \right\|_{L^\infty(K)}, \tag{73}$$

where we remark that K is assumed to be closed. Thus for the polynomial u, thanks to the inequality (65), we have

$$\sup_{i,j=1,\dots,R} |c_i - c_j| \leq \tilde{C} \sup_{i,j=1,\dots,R} |v_i - v_j| \leq \tilde{C} h \left\| \|\nabla_{\boldsymbol{x}} u\|_1 \right\|_{L^\infty(K)}, \tag{74}$$

because v_r are the values of u in the DoFs of K. □

We will continue now with the first proposition of this section, which will be used later in the proofs of the first order accuracy of $\mathcal{L}^1_\Delta$ and of the Lipschitz-continuity-like condition of $\mathcal{L}^1_\Delta - \mathcal{L}^2_\Delta$.

Proposition 3.4 (Mass lumping accuracy)**.** *Let us consider a scalar continuous piecewise polynomial function $u \in V_h$. We can write u as a linear combination of the Bernstein polynomials $\{\varphi_i\}_{i=1,\dots,I}$ associated to the tessellation which constitute a basis of V_h, i.e., $u(\boldsymbol{x}) = \sum_{i=1}^{I} c_i \varphi_i(\boldsymbol{x}) \quad \forall \boldsymbol{x} \in \overline{\Omega}$ with c_i scalar coefficients. Then, we have $\forall i = 1, \dots, I$ that*

$$\left| \sum_{K \in K_i} c_i \int_K \varphi_i(\boldsymbol{x}) d\boldsymbol{x} - \sum_{K \in K_i} \sum_{\boldsymbol{x}_j \in K} c_j \int_K \varphi_i(\boldsymbol{x}) \varphi_j(\boldsymbol{x}) d\boldsymbol{x} \right| \leq \hat{C} h \left\| \|\nabla_{\boldsymbol{x}} u\|_1 \right\|_{L^\infty(\mathcal{K}_i)} \int_{\mathcal{K}_i} |\varphi_i(\boldsymbol{x})| \, d\boldsymbol{x}, \tag{75}$$

with $h = \max_{K \in \mathcal{T}_h} diam(K)$ and $\hat{C}$ being a constant independent of h, dependent just on the dimension D, on the degree M and on the type of the elements in the mesh.

Proof. We will assume at first all the elements of the tessellation to be of the same type but this hypothesis can be relaxed to the general case with different types of elements.

Let us focus on the left-hand side of (75). Thanks to the normalization (62) of the basis functions and to the fact that the only basis functions that are not identically zero in the element K are the ones associated to the DoFs contained in that element, we can write

$$\left| \sum_{K \in K_i} c_i \int_K \varphi_i(\boldsymbol{x}) d\boldsymbol{x} - \sum_{K \in K_i} \sum_{\boldsymbol{x}_j \in K} c_j \int_K \varphi_i(\boldsymbol{x}) \varphi_j(\boldsymbol{x}) d\boldsymbol{x} \right| = \left| \sum_{K \in K_i} \sum_{\boldsymbol{x}_j \in K} (c_i - c_j) \int_K \varphi_i(\boldsymbol{x}) \varphi_j(\boldsymbol{x}) d\boldsymbol{x} \right|. \tag{76}$$

Now, thanks to the triangular inequality, to the fact that the absolute value of the basis functions φ_j can be bounded by a constant C_0, independent of the size of K, dependent just on the dimension D, on the degree M and on the type of the elements in the tessellation and also to the fact that the number R of DoFs $\boldsymbol{x}_j$ in each element K is fixed since D and M are fixed, we can write

$$\begin{aligned} &\left| \sum_{K \in K_i} \sum_{\boldsymbol{x}_j \in K} (c_i - c_j) \int_K \varphi_i(\boldsymbol{x}) \varphi_j(\boldsymbol{x}) d\boldsymbol{x} \right| \leq \sum_{K \in K_i} \sum_{\boldsymbol{x}_j \in K} |c_i - c_j| \left| \int_K \varphi_i(\boldsymbol{x}) \varphi_j(\boldsymbol{x}) d\boldsymbol{x} \right| \\ &\leq \sum_{K \in K_i} \sum_{\boldsymbol{x}_j \in K} \sup_{\boldsymbol{x}_\ell \in K} |c_i - c_\ell| \int_K |\varphi_i(\boldsymbol{x})||\varphi_j(\boldsymbol{x})| d\boldsymbol{x} \leq \sum_{K \in K_i} \sum_{\boldsymbol{x}_j \in K} C_0 \sup_{\boldsymbol{x}_\ell \in K} |c_i - c_\ell| \int_K |\varphi_i(\boldsymbol{x})| d\boldsymbol{x} \\ &\leq \sum_{K \in K_i} R C_0 \sup_{\boldsymbol{x}_\ell \in K} |c_i - c_\ell| \int_K |\varphi_i(\boldsymbol{x})| d\boldsymbol{x}. \end{aligned} \tag{77}$$

By applying the previous proposition (72) and from the fact that by definition $\mathcal{K}_i = \cup_{K \in K_i} K$, we can continue the sequence of inequalities and get

$$\begin{aligned} &\sum_{K \in K_i} R C_0 \sup_{\boldsymbol{x}_\ell \in K} |c_i - c_\ell| \int_K |\varphi_i(\boldsymbol{x})| d\boldsymbol{x} \leq \sum_{K \in K_i} R C_0 \tilde{C} h \, \| \| \nabla_{\boldsymbol{x}} u \|_1 \|_{L^\infty(K)} \int_K |\varphi_i(\boldsymbol{x})| d\boldsymbol{x} \\ \leq & R C_0 \tilde{C} h \, \| \| \nabla_{\boldsymbol{x}} u \|_1 \|_{L^\infty(\mathcal{K}_i)} \sum_{K \in K_i} \int_K |\varphi_i(\boldsymbol{x})| d\boldsymbol{x} = R C_0 \tilde{C} h \, \| \| \nabla_{\boldsymbol{x}} u \|_1 \|_{L^\infty(\mathcal{K}_i)} \int_{\mathcal{K}_i} |\varphi_i(\boldsymbol{x})| \, d\boldsymbol{x}. \end{aligned} \tag{78}$$

We take $\hat{C} = R C_0 \tilde{C}$ and we have the thesis, in fact, none of R, C_0 and $\tilde{C}$ depend on h, but they just depend on the dimension D, on the degree M and on the type of the elements in the tessellation.

We remark that we assumed that all the elements of the tessellation were of the same type. To deal with the general case in which we have different types of elements we suffice to take $\tilde{C}$ as the maximum of the coefficients $\tilde{C}$ of lemma 3.3 associated to the different types of elements and R as the highest number of degrees of freedom in a single element.

□

Before going ahead let us make some useful observations.

Remark 3.2. *Since the Bernstein basis functions are not negative, we can actually remove the absolute value inside the integral in* (75)*. We left it on purpose to be more general. In fact, it is easy to see that what is proved in this section is actually not limited to the specific case of*

Bernstein polynomials; the results can be easily extended to other polynomial bases, like for example the Lagrange polynomials (for which the matrix T is the identity and the constant $\tilde{C} = 1$) provided that the normalization (62) holds, i.e. $\sum_{i=1}^{I} \varphi(\boldsymbol{x}) \equiv 1$.

Remark 3.3. *The final result (75), which has been proven for a scalar polynomial $u \in V_h$, can be easily extended to the vectorial case by applying it componentwise. If $\boldsymbol{u} \in V_h^Q$, then we have*

$$\boldsymbol{u}(\boldsymbol{x}) = \sum_{i=1}^{I} \boldsymbol{c}_i \varphi_i(\boldsymbol{x}) \quad \forall \boldsymbol{x} \in \overline{\Omega} \tag{79}$$

with $\boldsymbol{c}_i \in \mathbb{R}^Q$ $\forall i = 1, \dots, I$ being Q-dimensional vectors of coefficients and $\{\varphi_i\}_{i=1,\dots,I}$ the Bernstein basis and it holds that

$$\begin{aligned} &\left\| \sum_{K \in K_i} \left(\int_K \varphi_i(\boldsymbol{x}) d\boldsymbol{x} \right) \boldsymbol{c}_i - \sum_{K \in K_i} \sum_{\boldsymbol{x}_j \in K} \left(\int_K \varphi_i(\boldsymbol{x}) \varphi_j(\boldsymbol{x}) d\boldsymbol{x} \right) \boldsymbol{c}_j \right\|_\infty \\ &\leq \hat{C} h \left\| \left\| \|\nabla_{\boldsymbol{x}} \boldsymbol{u}\|_1 \right\|_{L^\infty(\mathcal{K}_i)} \right\|_\infty \int_{\mathcal{K}_i} |\varphi_i(\boldsymbol{x})| \, d\boldsymbol{x} \quad \forall i = 1, \dots, I \end{aligned} \tag{80}$$

where the norms $\|\cdot\|_1$ and $\|\cdot\|_{L^\infty(\mathcal{K}_i)}$ are applied to each scalar component while the norm $\|\cdot\|_\infty$ is on $\mathbb{R}^Q$.

The key point is that the result (75) is uniform with respect to all the components of $\boldsymbol{u}$ and so we can easily take the infinity norm of both sides to pass from the scalar to the vectorial case.

We focus now on another intermediate lemma before proving the second and final proposition of this section.

Lemma 3.5. *Let $z \in C^1(K)$ and assume that its gradient is bounded in such a way that $\left\| \|\nabla_{\boldsymbol{x}} z\|_1 \right\|_{L^\infty(K)} \leq C_g$. Then, for K small enough it holds*

$$\|z\|_{L^1(K)} \geq C^* \|z\|_{L^\infty(K)} |K|, \tag{81}$$

with $|K|$ measure of K and C^* a constant dependent on C_g and on $\|z\|_{L^\infty(K)}$ but independent of the size of K.

Proof. As K is closed and $z \in C^1(K)$, then

$$\exists \boldsymbol{x}^* \in K \text{ s.t. } |z(\boldsymbol{x}^*)| = \|z\|_{L^\infty(K)} < +\infty. \tag{82}$$

Further, due to the continuity of z, the set B of the points in K for which the absolute value of the function is larger or equal than $\frac{|z(\boldsymbol{x}^*)|}{2}$ is non-empty and has a strictly positive measure, i.e. $|B| > 0$ with

$$B := \left\{ \boldsymbol{x} \in K \text{ s.t. } |z(\boldsymbol{x})| \geq \frac{|z(\boldsymbol{x}^*)|}{2} \right\}. \tag{83}$$

We try now to find a lower bound for $|B|$ by defining a set $B^* \subseteq B$ whose measure is known; in particular we define

$$B^* := \left\{ \boldsymbol{x} \in K \text{ s.t. } d(\boldsymbol{x}, \boldsymbol{x}^*) \leq \frac{|z(\boldsymbol{x}^*)|}{2C_g} \right\}. \tag{84}$$

where $d(\cdot,\cdot)$ is the Euclidean distance. Indeed, we have that $B^* \subseteq B$. Let $\tilde{\boldsymbol{x}} \in B^*$, then by a simple Taylor expansion we get

$$|z(\tilde{\boldsymbol{x}})| = |z(\boldsymbol{x}^*) + \nabla_{\boldsymbol{x}} z(\boldsymbol{s})(\boldsymbol{s} - \boldsymbol{x}^*)| \tag{85}$$

with $\boldsymbol{s}$ being a point, dependent on $\tilde{\boldsymbol{x}}$, contained in the segment $S(\tilde{\boldsymbol{x}}, \boldsymbol{x}^*)$ connecting $\tilde{\boldsymbol{x}}$ and $\boldsymbol{x}^*$. The triangle inequality gives

$$|z(\tilde{\boldsymbol{x}})| = |z(\boldsymbol{x}^*) + \nabla_{\boldsymbol{x}} z(\boldsymbol{s})(\boldsymbol{s} - \boldsymbol{x}^*)| \geq |z(\boldsymbol{x}^*)| - |\nabla_{\boldsymbol{x}} z(\boldsymbol{s})(\boldsymbol{s} - \boldsymbol{x}^*)|. \tag{86}$$

Now, we have that $|\nabla_{\boldsymbol{x}} z(\boldsymbol{s})(\boldsymbol{s} - \boldsymbol{x}^*)| \leq \frac{|z(\boldsymbol{x}^*)|}{2}$ because of the regularity assumption on the gradient of z and because $d(\boldsymbol{s}, \boldsymbol{x}^*) \leq d(\tilde{\boldsymbol{x}}, \boldsymbol{x}^*)$ as $\boldsymbol{s}$ belongs to the segment $S(\tilde{\boldsymbol{x}}, \boldsymbol{x}^*)$. This can be seen by simple computations:

$$|\nabla_{\boldsymbol{x}} z(\boldsymbol{s})(\boldsymbol{s} - \boldsymbol{x}^*)| \leq \big\| \|\nabla_{\boldsymbol{x}} z\|_1 \big\|_{L^\infty(K)} \, d(\boldsymbol{s}, \boldsymbol{x}^*) \leq C_g \frac{|z(\boldsymbol{x}^*)|}{2C_g} = \frac{|z(\boldsymbol{x}^*)|}{2}. \tag{87}$$

Coming back to (86) with this information, we can write

$$|z(\tilde{\boldsymbol{x}})| \geq |z(\boldsymbol{x}^*)| - |\nabla_{\boldsymbol{x}} z(\boldsymbol{s})(\boldsymbol{s} - \boldsymbol{x}^*)| \geq |z(\boldsymbol{x}^*)| - \frac{|z(\boldsymbol{x}^*)|}{2} = \frac{|z(\boldsymbol{x}^*)|}{2} \tag{88}$$

and hence $\tilde{\boldsymbol{x}} \in B$ and $B^* \subseteq B$.

We are able to estimate the measure of B^* providing therefore a lower bound for $|B|$, indeed, by definition, such set is the intersection between K and the ball $B_\rho(\boldsymbol{x}^*)$ centered in $\boldsymbol{x}^*$ with radius $\rho := \frac{|z(\boldsymbol{x}^*)|}{2C_g}$. If the ball $B_\rho(\boldsymbol{x}^*)$ is entirely contained in K then $B^* = B_\rho(\boldsymbol{x}^*)$ and its measure is given by $|B^*| = |B_\rho(\boldsymbol{x}^*)| = C_s \rho^D$ where C_s is the measure of the unitary ball in $\mathbb{R}^D$. If this does not hold, it is anyway always possible to find a lower bound for the measure of B^* of the type

$$|B^*| \geq \min\left(C_\alpha \rho^D, |K|\right) \tag{89}$$

with C_α constant dependent only on the aspect ratio of K but not on its size. Therefore, from the definition of B and from $|B| \geq |B^*| \geq \min\left(C_\alpha \rho^D, |K|\right)$, we get

$$\|z\|_{L^1(K)} = \int_K |z(\boldsymbol{x})| d\boldsymbol{x} \geq \int_B |z(\boldsymbol{x})| d\boldsymbol{x} \geq \frac{|z(\boldsymbol{x}^*)|}{2} |B| \geq \frac{|z(\boldsymbol{x}^*)|}{2} |B^*| \geq \frac{|z(\boldsymbol{x}^*)|}{2} \min\left(C_\alpha \rho^D, |K|\right). \tag{90}$$

Now, recalling that $|z(\boldsymbol{x}^*)| = \|z\|_{L^\infty(K)}$, we have

$$\|z\|_{L^1(K)} \geq \frac{\|z\|_{L^\infty(K)}}{2} |K| \min\left(\frac{C_\alpha \rho^D}{|K|}, 1\right) \tag{91}$$

We define thus $C^* := \frac{1}{2} \min\left(\frac{C_\alpha \rho^D}{|K|}, 1\right)$ and we observe that, since C_α only depends on geometrical properties of K and ρ only depends on z, for K small enough $C^* = \frac{1}{2}$ and we get the thesis.

□

Now, let us generalize this result to the whole domain for piecewise C^1 functions, even discontinuous, by proving the last result of this section.

Proposition 3.6 (Relation between L^∞ and L^1 norms). *Let $z \in \left\{ z \in L^1(\Omega) \text{ s.t. } z|_K \in C^1(K),\, \forall K \in \mathcal{T}_h \right\}$ satisfying locally in each element the hypotheses of the previous lemma, i.e. $\| \|\nabla_{\boldsymbol{x}} z\|_1 \|_{L^\infty(K)} \le C_g$ and K small enough. Assume the mesh to be regular in the sense that for any $i = 1, \ldots, I$ it holds that*

$$\int_K |\varphi_i(\boldsymbol{x})| d\boldsymbol{x} \le C_{\mathcal{M}} \int_{\tilde{K}} |\varphi_i(\boldsymbol{x})| d\boldsymbol{x}, \qquad \forall K, \tilde{K} \in K_i, \tag{92}$$

where $\{\varphi_i\}_{i=1,\ldots,I}$ is the basis of V_h given by Bernstein polynomials. Then,

$$\sum_{i=1}^{I} \|z\|_{L^\infty(\mathcal{K}_i)} \sum_{K \in K_i} \int_K |\varphi_i(\boldsymbol{x})| d\boldsymbol{x} \le \tilde{C}^* \|z\|_{L^1(\Omega)}, \tag{93}$$

where $\tilde{C}^*$ is a positive constant independent of the mesh parameter.

Proof. Let $K^i \in K_i$ be the element such that $\|z\|_{L^\infty(\mathcal{K}_i)} = \|z\|_{L^\infty(K^i)}$; then, using the mesh regularity assumption (92) and the fact that the basis functions are bounded in absolute value by a constant C_0 independent of the mesh parameter, we have

$$\begin{aligned}
\sum_{i=1}^{I} \|z\|_{L^\infty(\mathcal{K}_i)} \sum_{K \in K_i} \int_K |\varphi_i(\boldsymbol{x})| d\boldsymbol{x} &= \sum_{i=1}^{I} \sum_{K \in K_i} \|z\|_{L^\infty(\mathcal{K}_i)} \int_K |\varphi_i(\boldsymbol{x})| d\boldsymbol{x} \\
&= \sum_{i=1}^{I} \sum_{K \in K_i} \|z\|_{L^\infty(K^i)} \int_K |\varphi_i(\boldsymbol{x})| d\boldsymbol{x} \\
&\le \sum_{i=1}^{I} \sum_{K \in K_i} C_{\mathcal{M}} \|z\|_{L^\infty(K^i)} \int_{K^i} |\varphi_i(\boldsymbol{x})| d\boldsymbol{x} \\
&\le \sum_{i=1}^{I} \sum_{K \in K_i} C_{\mathcal{M}} C_0 \|z\|_{L^\infty(K^i)} |K^i|.
\end{aligned} \tag{94}$$

We apply now the previous lemma 3.5 and, switching the sums over the elements and the DoFs, we get

$$\begin{aligned}
\sum_{i=1}^{I} \sum_{K \in K_i} C_{\mathcal{M}} C_0 \|z\|_{L^\infty(K^i)} |K^i| &\le \sum_{i=1}^{I} \sum_{K \in K_i} \frac{C_{\mathcal{M}} C_0}{C^*} \|z\|_{L^1(K^i)} \\
&= \frac{C_{\mathcal{M}} C_0}{C^*} \sum_{K \in \mathcal{T}_h} \sum_{\boldsymbol{x}_i \in K} \|z\|_{L^1(K^i)}
\end{aligned} \tag{95}$$

where C^* is the minimal coefficient of lemma 3.5 among the ones associated to all the elements K^i. If R is the maximal number of DoFs in a single element in the whole mesh, we can continue and write

$$\frac{C_{\mathcal{M}} C_0}{C^*} \sum_{K \in \mathcal{T}_h} \sum_{\boldsymbol{x}_i \in K} \|z\|_{L^1(K^i)} \le \frac{R C_{\mathcal{M}} C_0}{C^*} \sum_{K \in \mathcal{T}_h} \sup_{\boldsymbol{x}_i \in K} \|z\|_{L^1(K^i)}. \tag{96}$$

Now, in (96), each element K in the tessellation is contributing to the sum with the L^1 norm of z over one element K^i among the ones associated to the DoFs $\boldsymbol{x}_i \in K$. The generic element K^i can

be present in the sum at most a number of times equal to M_n+1 where M_n represents the maximal number of neighbors that an element can have in the tessellation. Hence, we get

$$\frac{RC_{\mathcal{M}}C_0}{C^*}\sum_{K\in\mathcal{T}_h}\sup_{\boldsymbol{x}_i\in K}\|z\|_{L^1(K^i)}\leq\frac{RC_{\mathcal{M}}C_0}{C^*}(M_n+1)\|z\|_{L^1(\Omega)}\,. \tag{97}$$

Observe that none of the coefficients R, $C_{\mathcal{M}}$, C_0, C^* or M_n depend on the mesh parameter, therefore, by setting $\tilde{C}^*=\frac{RC_{\mathcal{M}}C_0}{C^*}(M_n+1)$, we get the thesis. □

Also in this case, we remark that, in the context of Bernstein polynomials, which are non-negative, the absolute value on φ_i is not necessary. We kept it just to be more general. Indeed, all the results can be generalized to other basis functions like the Lagrange polynomials.

3.1.2 Definition of $\mathcal{L}^2_\Delta$

The operator $\mathcal{L}^2_\Delta$ is the high order implicit operator that we would like to solve. Its definition is not very different from the one seen in the context of the bDeC for ODEs. We introduce the $M+1$ subtimenodes t^m with $m=0,\dots,M$ in the interval $[t_n,t_n+\Delta t]$ in which we will consider the approximations of the values of the solution to our system of ODEs. We refer to $\boldsymbol{c}(t^m)$ as the exact solution in the node t^m and to $\boldsymbol{c}^m$ as the approximation of the solution in the same node. Clearly, in this case $\boldsymbol{c}(t^m)$ and $\boldsymbol{c}^m$ contain as components all the coefficients corresponding to the spatial DoFs, i.e., respectively the vectors $\boldsymbol{c}_i(t^m)$ of the exact coefficients in the DoFs at the time t^m and the vectors $\boldsymbol{c}_i^m$ of the approximated ones. As usual, for the first subtimenode we set $\boldsymbol{c}^0=\boldsymbol{c}(t^0)=\boldsymbol{c}(t_n)=\boldsymbol{c}_n$ without any approximation. Starting from the exact integration of (56) over $[t^0,t^m]$ and substituting $\boldsymbol{\phi}_i(\boldsymbol{c}(t))$ with its M-th order interpolation in time associated to the $M+1$ subtimenodes, we get

$$\sum_{K\in K_i}\sum_{\boldsymbol{x}_j\in K}\left(\int_K\varphi_i(\boldsymbol{x})\varphi_j(\boldsymbol{x})d\boldsymbol{x}\right)\left(\boldsymbol{c}_j^m-\boldsymbol{c}_j^0\right)+\Delta t\sum_{\ell=0}^{M}\theta_\ell^m\boldsymbol{\phi}_i(\boldsymbol{c}^\ell)=\boldsymbol{0},\ \forall i=1,\dots,I\ \forall m=1,\dots,M. \tag{98}$$

Therefore, we can define the operator $\mathcal{L}^2_\Delta:\mathbb{R}^{(I\times Q\times M)}\to\mathbb{R}^{(I\times Q\times M)}$ as

$$\mathcal{L}^2_\Delta(\underline{\boldsymbol{c}})=\left(\mathcal{L}^2_{\Delta,1}(\underline{\boldsymbol{c}}),\mathcal{L}^2_{\Delta,2}(\underline{\boldsymbol{c}}),\dots,\mathcal{L}^2_{\Delta,I}(\underline{\boldsymbol{c}})\right),\qquad\forall\underline{\boldsymbol{c}}\in\mathbb{R}^{(I\times Q\times M)}, \tag{99}$$

where for any i we have

$$\mathcal{L}^2_{\Delta,i}(\underline{\boldsymbol{c}})=\begin{pmatrix}\sum_{K\in K_i}\sum_{\boldsymbol{x}_j\in K}\left(\int_K\varphi_i(\boldsymbol{x})\varphi_j(\boldsymbol{x})d\boldsymbol{x}\right)\left(\boldsymbol{c}_j^1-\boldsymbol{c}_j^0\right)+\Delta t\sum_{\ell=0}^M\theta_\ell^1\boldsymbol{\phi}_i(\boldsymbol{c}^\ell)\\ \vdots\\ \sum_{K\in K_i}\sum_{\boldsymbol{x}_j\in K}\left(\int_K\varphi_i(\boldsymbol{x})\varphi_j(\boldsymbol{x})d\boldsymbol{x}\right)\left(\boldsymbol{c}_j^m-\boldsymbol{c}_j^0\right)+\Delta t\sum_{\ell=0}^M\theta_\ell^m\boldsymbol{\phi}_i(\boldsymbol{c}^\ell)\\ \vdots\\ \sum_{K\in K_i}\sum_{\boldsymbol{x}_j\in K}\left(\int_K\varphi_i(\boldsymbol{x})\varphi_j(\boldsymbol{x})d\boldsymbol{x}\right)\left(\boldsymbol{c}_j^M-\boldsymbol{c}_j^0\right)+\Delta t\sum_{\ell=0}^M\theta_\ell^M\boldsymbol{\phi}_i(\boldsymbol{c}^\ell)\end{pmatrix}. \tag{100}$$

with the general argument $\underline{\boldsymbol{c}}\in\mathbb{R}^{(I\times Q\times M)}$ having M components $\boldsymbol{c}^m\in\mathbb{R}^{(I\times Q)}$ each one associated to a subtimenode and having I components $\boldsymbol{c}_i^m$ each one associated to a DoF.

The solution $\underline{\boldsymbol{c}}_\Delta$ to $\mathcal{L}^2_\Delta(\underline{\boldsymbol{c}}_\Delta)=\boldsymbol{0}$ is $(M+1)$-th order accurate in the sense that would contain as components $(M+1)$-th order accurate approximations of the coefficients which represent the exact solution to (56) in all the subtimenodes t^m $m=1,\dots,M$. Unfortunately, the problem $\mathcal{L}^2_\Delta(\underline{\boldsymbol{c}})=\boldsymbol{0}$ is a huge nonlinear system.

3.1.3 Definition of $\mathcal{L}^1_\Delta$

Performing an Euler approximation in time to numerically solve (56) in $[t^0, t^m]$ we get

$$\sum_{K \in K_i} \sum_{\boldsymbol{x}_j \in K} \left(\int_K \varphi_i(\boldsymbol{x}) \varphi_j(\boldsymbol{x}) d\boldsymbol{x} \right) \left(\boldsymbol{c}_j^m - \boldsymbol{c}_j^0 \right) + \Delta t \beta^m \boldsymbol{\phi}_i(\boldsymbol{c}^0) = \boldsymbol{0}, \quad \forall i = 1, \dots, I, \quad \forall m = 1, \dots, M. \tag{101}$$

Further, we perform a first order mass lumping in space to get a fully explicit approximation formula for $\boldsymbol{c}_i^m$

$$C_i \left(\boldsymbol{c}_i^m - \boldsymbol{c}_i^0 \right) + \Delta t \beta^m \boldsymbol{\phi}_i(\boldsymbol{c}^0) = \boldsymbol{0}, \quad \forall i = 1, \dots, I \quad \forall m = 1, \dots, M \tag{102}$$

where C_i are constant quantities defined as

$$C_i := \int_\Omega \varphi_i(\boldsymbol{x}) d\boldsymbol{x} = \sum_{K \in K_i} \int_K \varphi_i(\boldsymbol{x}) d\boldsymbol{x}, \quad \forall i = 1, \dots, I. \tag{103}$$

We assume a choice of the basis functions such that $C_i \neq 0 \ \forall i$ so that (102) is well-posed. For example, if we choose the Bernstein polynomials, we have $C_i > 0 \ \forall i$ as the basis functions φ_i are nonnegative. Indeed, $\boldsymbol{c}_i^m$ got from (102) is a first order approximation of the exact coefficient $\boldsymbol{c}_i(t^m)$, as proved in the next proposition.

Proposition 3.7 (First order accuracy of (102))**.** *The solution to* (102) *is first order accurate with respect to the exact solution* $\boldsymbol{c}(t)$ *to* (56) *evaluated in all the subtimenodes* t^m *for* $m = 1, \dots, M$.

Proof. We can equivalently show that if we insert the exact solution to (56) evaluated in all the subtimenodes t^m $m = 1, \dots, M$ into the left-hand side of (102) we get an error $O(\Delta^{D+2})$ where D is the number of spatial dimensions and the parameter Δ is the mesh parameter h of the space discretization. Therefore, we want to prove that

$$C_i \left(\boldsymbol{c}_i(t^m) - \boldsymbol{c}_i^0 \right) + \Delta t \beta^m \boldsymbol{\phi}_i(\boldsymbol{c}^0) = O(\Delta^{D+2}), \quad \forall i = 1, \dots, I, \quad \forall m = 1, \dots, M. \tag{104}$$

We know that by plugging the exact solution $\boldsymbol{c}(t)$ in (101) we get an error $O(\Delta^{D+2})$:

$$\sum_{K \in K_i} \sum_{\boldsymbol{x}_j \in K} \left(\int_K \varphi_i(\boldsymbol{x}) \varphi_j(\boldsymbol{x}) d\boldsymbol{x} \right) \left(\boldsymbol{c}_j(t^m) - \boldsymbol{c}_j^0 \right) + \Delta t \beta^m \boldsymbol{\phi}_i(\boldsymbol{c}^0) = O(\Delta^{D+2}). \tag{105}$$

Hence, instead of (104), we can show that the difference of (104) and (105) is an $O(\Delta^{D+2})$, i.e.,

$$C_i \left(\boldsymbol{c}_i(t^m) - \boldsymbol{c}_i^0 \right) - \sum_{K \in K_i} \sum_{\boldsymbol{x}_j \in K} \left(\int_K \varphi_i(\boldsymbol{x}) \varphi_j(\boldsymbol{x}) d\boldsymbol{x} \right) \left(\boldsymbol{c}_j(t^m) - \boldsymbol{c}_j^0 \right) = O(\Delta^{D+2}). \tag{106}$$

By definition of the coefficients C_i in (103) and the preliminary result (80), we can write

$$\begin{aligned} &\left\| C_i \left(\boldsymbol{c}_i(t^m) - \boldsymbol{c}_i^0 \right) - \sum_{K \in K_i} \sum_{\boldsymbol{x}_j \in K} \left(\int_K \varphi_i(\boldsymbol{x}) \varphi_j(\boldsymbol{x}) d\boldsymbol{x} \right) \left(\boldsymbol{c}_j(t^m) - \boldsymbol{c}_j^0 \right) \right\|_\infty \\ \leq &\hat{C} h \left\| \left\| \left\| \nabla_{\boldsymbol{x}} (\boldsymbol{u}_h(\boldsymbol{x}, t^m) - \boldsymbol{u}_h(\boldsymbol{x}, t^0)) \right\|_1 \right\|_{L^\infty(\mathcal{K}_i)} \right\|_\infty \int_{\mathcal{K}_i} |\varphi_i(\boldsymbol{x})| \, d\boldsymbol{x}, \end{aligned} \tag{107}$$

where we remark that the internal norms $\|\cdot\|_1$ and $\|\cdot\|_{L^\infty(\mathcal{K}_i)}$ are applied componentwise while the external one, $\|\cdot\|_\infty$, is on $\mathbb{R}^Q$. From a Taylor expansion it is easy to see that

$$\nabla_{\boldsymbol{x}}(\boldsymbol{u}_h(\boldsymbol{x}, t^m) - \boldsymbol{u}_h(\boldsymbol{x}, t^0)) = O(\Delta t). \tag{108}$$

Moreover, $\int_{\mathcal{K}_i} |\varphi_i(\boldsymbol{x})| d\boldsymbol{x} = O(\Delta^D)$, hence, we have

$$\left\| C_i \left(\boldsymbol{c}_i(t^m) - \boldsymbol{c}_i^0\right) - \sum_{K \in K_i} \sum_{\boldsymbol{x}_j \in K} \left(\int_K \varphi_i(\boldsymbol{x}) \varphi_j(\boldsymbol{x}) d\boldsymbol{x} \right) \left(\boldsymbol{c}_j(t^m) - \boldsymbol{c}_j^0\right) \right\|_\infty = O(\Delta^{D+2}). \tag{109}$$

□

Directly from (102), we can define the explicit low order operator $\mathcal{L}^1_\Delta : \mathbb{R}^{(I \times Q \times M)} \to \mathbb{R}^{(I \times Q \times M)}$ as

$$\mathcal{L}^1_\Delta(\underline{\boldsymbol{c}}) = \left(\mathcal{L}^1_{\Delta,1}(\underline{\boldsymbol{c}}), \mathcal{L}^1_{\Delta,2}(\underline{\boldsymbol{c}}), \dots, \mathcal{L}^1_{\Delta,I}(\underline{\boldsymbol{c}})\right), \quad \forall \underline{\boldsymbol{c}} \in \mathbb{R}^{(I \times Q \times M)}, \tag{110}$$

where for any i we have

$$\mathcal{L}^1_{\Delta,i}(\underline{\boldsymbol{c}}) = \begin{pmatrix} C_i \left(\boldsymbol{c}_i^1 - \boldsymbol{c}_i^0\right) + \Delta t \beta^1 \boldsymbol{\phi}_i(\boldsymbol{c}^0) \\ \vdots \\ C_i \left(\boldsymbol{c}_i^m - \boldsymbol{c}_i^0\right) + \Delta t \beta^m \boldsymbol{\phi}_i(\boldsymbol{c}^0) \\ \vdots \\ C_i \left(\boldsymbol{c}_i^M - \boldsymbol{c}_i^0\right) + \Delta t \beta^M \boldsymbol{\phi}_i(\boldsymbol{c}^0) \end{pmatrix}. \tag{111}$$

in which the convention on the indices of the components of the general argument $\underline{\boldsymbol{c}} \in \mathbb{R}^{(I \times Q \times M)}$ is the same that we had for the operator $\mathcal{L}^2_\Delta$.

3.1.4 Proof of the properties of $\mathcal{L}^1_\Delta$ and $\mathcal{L}^2_\Delta$

The operators $\mathcal{L}^1_\Delta$ and $\mathcal{L}^2_\Delta$ act from X to Y with $X = Y = \mathbb{R}^{(I \times Q \times M)}$. Let us recall again the hypotheses that are needed in order to apply the Deferred Correction method

i) **Existence of a solution to $\mathcal{L}^2_\Delta$**
$\exists ! \underline{\boldsymbol{u}}_\Delta \in \mathbb{R}^{(I \times Q \times M)}$ solution of $\mathcal{L}^2_\Delta$, i.e. such that $\mathcal{L}^2_\Delta(\underline{\boldsymbol{u}}_\Delta) = \boldsymbol{0}$;

ii) **Coercivity-like property of $\mathcal{L}^1_\Delta$**
$\exists \alpha_1 \geq 0$ independent of Δ s.t.

$$\left\|\mathcal{L}^1_\Delta(\underline{\boldsymbol{v}}) - \mathcal{L}^1_\Delta(\underline{\boldsymbol{w}})\right\|_Y \geq \alpha_1 \left\|\underline{\boldsymbol{v}} - \underline{\boldsymbol{w}}\right\|_X, \quad \forall \underline{\boldsymbol{v}}, \underline{\boldsymbol{w}} \in \mathbb{R}^{(I \times Q \times M)}; \tag{112}$$

iii) **Lipschitz-continuity-like condition of $\mathcal{L}^1_\Delta - \mathcal{L}^2_\Delta$**
$\exists \alpha_2 \geq 0$ independent of Δ s.t.

$$\left\| \left[\mathcal{L}^1_\Delta(\underline{\boldsymbol{v}}) - \mathcal{L}^2_\Delta(\underline{\boldsymbol{v}})\right] - \left[\mathcal{L}^1_\Delta(\underline{\boldsymbol{w}}) - \mathcal{L}^2_\Delta(\underline{\boldsymbol{w}})\right] \right\|_Y \leq \alpha_2 \Delta \left\|\underline{\boldsymbol{v}} - \underline{\boldsymbol{w}}\right\|_X, \quad \forall \underline{\boldsymbol{v}}, \underline{\boldsymbol{w}} \in \mathbb{R}^{(I \times Q \times M)}. \tag{113}$$

We remark that in this context the parameter Δ is the mesh parameter h and that we assume the temporal step size $\Delta t \leq Ch$ for some fixed constant C.

We will not prove the first property, i.e., the existence of a unique solution to $\mathcal{L}^2_\Delta$, because the proof is identical to the one we had in the ODE case up to the inversion of the mass matrix: from $\mathcal{L}^2_\Delta$ we can define an operator $\mathcal{J} : \mathbb{R}^{(I\times Q\times M)} \to \mathbb{R}^{(I\times Q\times M)}$ whose fixed points (if any) are solutions to $\mathcal{L}^2_\Delta$; further, we can show that for Δ small enough the operator is a contraction over the space $\mathbb{R}^{(I\times Q\times M)}$ equipped with the infinity norm and, hence, there exists a unique fixed point of $\mathcal{J}$ which is the unique solution to $\mathcal{L}^2_\Delta$.

Before going to the proofs of the other two properties, we need to define the norms adopted on the spaces X and Y. Despite having $X = Y = \mathbb{R}^{(I\times Q\times M)}$ we will equip X and Y with two different norms, differently from what we have done in the ODE case. We will specify the norms after the following useful observation.

Remark 3.4 (Remark on the indices)**.** *The main complication of the proofs is that we have to deal with many indices. We remind that*

- $i = 1, \dots, I$ *is referred to the DoFs;*
- $q = 1, \dots, Q$ *is referred to the components of the approximated solution* $\boldsymbol{u}_h$ *to the system of balance laws* (55)*;*
- $m = 1, \dots, M$ *is referred to the subtimenodes* t^m*, even if we remark that we also have an initial subtimenode* $t^0 = t_n$ *in which the quantities are not unknown.*

We are already used to the fact that the general element $\underline{\boldsymbol{c}} \in \mathbb{R}^{(I\times Q\times M)}$ *must be thought as a collection of* M *components* $\boldsymbol{c}^m \in \mathbb{R}^{(I\times Q)}$ $m = 1, \dots, M$*. Each component* $\boldsymbol{c}^m$ *can be thought as the vector of the coefficients of a vectorial continuous piecewise polynomial function* $\boldsymbol{u}_h(\boldsymbol{x}, t) = \sum_{i=1}^I \boldsymbol{c}_i(t)\varphi_i(\boldsymbol{x})$ *evaluated in the subtimenode* t^m*. In fact, each* $\boldsymbol{c}^m$ *is made by* I *components* $\boldsymbol{c}_i^m \in \mathbb{R}^Q$ *with* $i = 1, \dots, I$ *associated to the DoFs. Finally, each* $\boldsymbol{c}_i^m$ *is made by* Q *components* $c_i^{q,m}$ $q = 1, \dots, Q$*, scalar coefficients associated to the components of the solution to the system of PDEs that we would like to solve, i.e.,*

$$\underline{\boldsymbol{c}} = \begin{pmatrix} \boldsymbol{c}^1 \\ \vdots \\ \boldsymbol{c}^m \\ \vdots \\ \boldsymbol{c}^M \end{pmatrix} \in \mathbb{R}^{(I\times Q\times M)}, \quad \boldsymbol{c}^m = \begin{pmatrix} \boldsymbol{c}_1^m \\ \vdots \\ \boldsymbol{c}_i^m \\ \vdots \\ \boldsymbol{c}_I^m \end{pmatrix} \in \mathbb{R}^{(I\times Q)}, \quad \boldsymbol{c}_i^m = \begin{pmatrix} c_i^{1,m} \\ \vdots \\ c_i^{q,m} \\ \vdots \\ c_i^{Q,m} \end{pmatrix} \in \mathbb{R}^Q. \tag{114}$$

In the proofs, we are going to focus on a single scalar component $q = 1, \dots, Q$ of a single subtimenode $m = 1, \dots, M$ and our results will be uniform with respect to the indices q and m, so we will be able to pass from the scalar results to the desired vectorial results through an infinity norm $\|\cdot\|_\infty$ on $\mathbb{R}^{(Q\times M)}$, similarly to what we did when we passed from (75) to (80) in the preliminary results. Therefore, the norm that we choose for the single component of $\underline{\boldsymbol{c}} \in X = \mathbb{R}^{(I\times Q\times M)}$ with fixed indices q and m, denoted by $\boldsymbol{c}^{q,m} \in \mathbb{R}^I$, is the $W_I^{1,1}(\Omega)$-norm, a discrete version of the classical $W^{1,1}(\Omega)$-norm. In particular, on a scalar function $u : \Omega \to \mathbb{R}$ the $W^{1,1}(\Omega)$-norm is defined as

$$\|u\|_{W^{1,1}(\Omega)} := \|u\|_{L^1(\Omega)} + \sum_{d=1}^D \left\| \frac{\partial}{\partial x_d} u \right\|_{L^1(\Omega)} = \|u\|_{L^1(\Omega)} + \left\| \|\nabla_{\boldsymbol{x}} u\|_1 \right\|_{L^1(\Omega)}, \tag{115}$$

from which we define the corresponding discrete norm on $\mathbb{R}^I$, defined by $\|\cdot\|_{W_I^{1,1}(\Omega)} : \mathbb{R}^I \to \mathbb{R}_0^+$ as

$$\|\boldsymbol{c}^{q,m}\|_{W_I^{1,1}(\Omega)} := \left\| \sum_{i=1}^{I} c_i^{q,m} \varphi_i \right\|_{W^{1,1}(\Omega)}. \tag{116}$$

Using then the classical infinity norm on the space $\mathbb{R}^{Q\times M}$ defined by $\|\cdot\|_{\infty,Q,M} : \mathbb{R}^{Q\times M} \to \mathbb{R}_0^+$, we introduce the X norm $\|\cdot\|_X : \mathbb{R}^{I\times Q\times M} \to \mathbb{R}_0^+$ as

$$\|\underline{\boldsymbol{c}}\|_X := \left\| \left\{ \|\boldsymbol{c}^{q,m}\|_{W_I^{1,1}(\Omega)} \right\}_{\substack{q=1,\dots,Q \\ m=1,\dots,M}} \right\|_{\infty,Q,M}. \tag{117}$$

Instead, we equip Y with a different norm; we choose for the single component $\tilde{\boldsymbol{c}}^{q,m} \in \mathbb{R}^I$ with fixed indices q and m of $\underline{\tilde{\boldsymbol{c}}} \in Y = \mathbb{R}^{(I\times Q\times M)}$ the 1-norm $\|\cdot\|_{1,I} : \mathbb{R}^I \to \mathbb{R}_0^+$ defined as

$$\|\tilde{\boldsymbol{c}}^{q,m}\|_{1,I} := \sum_{i=1}^{I} |\tilde{c}_i^{q,m}|, \tag{118}$$

then the norm on the whole space $Y = \mathbb{R}^{(I\times Q\times M)}$, $\|\cdot\|_Y : \mathbb{R}^{(I\times Q\times M)} \to \mathbb{R}_0^+$, is defined by

$$\|\underline{\tilde{\boldsymbol{c}}}\|_Y = \left\| \left\{ \|\tilde{\boldsymbol{c}}^{q,m}\|_{1,I} \right\}_{\substack{q=1,\dots,Q \\ m=1,\dots,M}} \right\|_{\infty,Q,M}. \tag{119}$$

Remark 3.5. *We remark that the initial subtimenode $m = 0$ is not kept into account in the norms* (117) *and* (119) *as it is a datum of the problem.*

Remark 3.6 (On the choice of the norms)**.** *The reason of the difference in the norms assumed on X and Y is intuitively due to the following fact. Practically speaking, the elements of X, the arguments of $\mathcal{L}^2_\Delta$ and $\mathcal{L}^1_\Delta$ given respectively by* (99) *and* (110) *(and so by* (100) *and* (111)*), are the coefficients associated to a vectorial continuous piecewise polynomial function evaluated in the subtimenodes t^m $m = 1,\dots,M$. Therefore, on the space X we take an integral norm for "functions". Instead, the elements of the space Y, the images of $\mathcal{L}^2_\Delta$ and $\mathcal{L}^1_\Delta$, are consistent with integrals of the mentioned function associated to the coefficients. In order to guarantee the consistency of the terms in the inequalities to prove and to compare $\|\cdot\|_X$ and $\|\cdot\|_Y$, we must take for Y a norm which does not modify the integral "character" of the components of the elements of the space.*

It is straightforward to prove that (117) and (119) are norms but we will not do it for the sake of brevity. In the context of the proofs of the properties of $\mathcal{L}^2_\Delta$ and $\mathcal{L}^1_\Delta$, we are going to make use of the two following regularity assumptions.

Assumption 3.8 (Poincaré-like inequality)**.** *We assume that we are working with coefficients regular enough to guarantee that the associated functions $\boldsymbol{g}_h$, for some $C_p \geq 0$ independent of Δ, are such that*

$$\|\boldsymbol{g}_h\|_{W^{1,1}(\Omega)} \leq C_p \|\boldsymbol{g}_h\|_{L^1(\Omega)}, \tag{120}$$

i.e., we assume that we can control the norm of the gradient of all functions that we will consider with the norm of the functions.

Assumption 3.9 (Smoothness of the space residuals)**.** *We assume the functions $\boldsymbol{\phi}_i$ defined in* (57) *to be smooth.*

Finally, the notation in eq. (114) will hold for two generic vectors $\underline{\boldsymbol{v}}, \underline{\boldsymbol{w}} \in \mathbb{R}^{(I\times Q\times M)}$ that will be used in the proof.

In order to deal with the single component got for fixed m and q, as we are going to do in a few lines, it is very useful to define here the scalar continuous piecewise polynomial functions

$$v_h^{q,m}(\boldsymbol{x}) = \sum_{i=1}^{I} v_i^{q,m}\varphi_i(\boldsymbol{x}), \quad w_h^{q,m}(\boldsymbol{x}) = \sum_{i=1}^{I} w_i^{q,m}\varphi_i(\boldsymbol{x}) \tag{121}$$

associated to the scalar coefficients $v_i^{q,m}$ and $w_i^{q,m}$ $i = 1, \dots, I$.

Now, we have all the elements that we need in order to handle the proofs of the properties of the two operators.

Proposition 3.10 (Coercivity-like property of $\mathcal{L}^1_\Delta$)**.** *Let $\mathcal{L}^1_\Delta : X \to Y$ be the operator defined in* (110) *and* (111)*, $\underline{\boldsymbol{v}}, \underline{\boldsymbol{w}} \in X$ and suppose that assumption 3.8 holds, then $\exists \alpha_1 > 0$ independent of Δ s.t.*

$$\left\|\mathcal{L}^1_\Delta(\underline{\boldsymbol{v}}) - \mathcal{L}^1_\Delta(\underline{\boldsymbol{w}})\right\|_Y \geq \alpha_1 \left\|\underline{\boldsymbol{v}} - \underline{\boldsymbol{w}}\right\|_X, \quad \forall \underline{\boldsymbol{v}}, \underline{\boldsymbol{w}} \in \mathbb{R}^{(I\times Q\times M)}. \tag{122}$$

Proof. From a direct computation we have, for every $i = 1, \dots, I$, $m = 1, \dots, M$ and $q = 1, \dots, Q$, that

$$\begin{aligned}\mathcal{L}^{1,q,m}_{\Delta,i}(\underline{\boldsymbol{v}}) - \mathcal{L}^{1,q,m}_{\Delta,i}(\underline{\boldsymbol{w}}) &= C_i\left(v_i^{q,m} - c_i^{0,q}\right) + \Delta t \beta^m \boldsymbol{\phi}_i^q(\boldsymbol{c}^0) - C_i\left(w_i^{q,m} - c_i^{0,q}\right) - \Delta t\beta^m \boldsymbol{\phi}_i^q(\boldsymbol{c}^0)\\ &= C_i\left(v_i^{q,m} - w_i^{q,m}\right).\end{aligned} \tag{123}$$

We remark again that $\boldsymbol{c}^0$ is known and so also $\boldsymbol{c}_i^0$. We will start by proving the coercivity-like property for a fixed component q and a fixed subtimenode m, i.e., we will prove that the 1-norm of (123) over the indexes $i = 1, \dots, I$ is such that

$$\left\|\mathcal{L}^{1,q,m}_{\Delta}(\underline{\boldsymbol{v}}) - \mathcal{L}^{1,q,m}_{\Delta}(\underline{\boldsymbol{w}})\right\|_{1,I} \geq \alpha_1 \left\|v_h^{q,m} - w_h^{q,m}\right\|_{W^{1,1}(\Omega)} \tag{124}$$

for some α_1 independent of Δ for all m and q. Recalling the definition (103) of the coefficients $C_i = \int_\Omega \varphi_i(\boldsymbol{x})d\boldsymbol{x}$ and the fact that the Bernstein basis functions are nonnegative, we have

$$\left\|\mathcal{L}^{1,q,m}_{\Delta}(\underline{\boldsymbol{v}}) - \mathcal{L}^{1,q,m}_{\Delta}(\underline{\boldsymbol{w}})\right\|_{1,I} = \sum_{i=1}^{I}\left|C_i\left(v_i^{q,m} - w_i^{q,m}\right)\right| = \sum_{i=1}^{I}\int_\Omega \left|(v_i^{q,m} - w_i^{q,m})\varphi_i(\boldsymbol{x})\right| d\boldsymbol{x}. \tag{125}$$

Using the triangular inequality and recalling the definition (121) of the scalar continuous piecewise polynomial functions $v_h^{q,m}$ and $w_h^{q,m}$, from the previous equation we get

$$\begin{aligned}\left\|\mathcal{L}^{1,q,m}_{\Delta}(\underline{\boldsymbol{v}}) - \mathcal{L}^{1,q,m}_{\Delta}(\underline{\boldsymbol{w}})\right\|_{1,I} &\geq \int_\Omega \left|\sum_{i=1}^{I}(v_i^{q,m} - w_i^{q,m})\varphi_i(\boldsymbol{x})\right| d\boldsymbol{x}\\ &= \int_\Omega \left|v_h^{q,m}(\boldsymbol{x}) - w_h^{q,m}(\boldsymbol{x})\right| d\boldsymbol{x} = \left\|v_h^{q,m} - w_h^{q,m}\right\|_{L^1(\Omega)}\\ &\geq \frac{1}{C_p}\left\|v_h^{q,m} - w_h^{q,m}\right\|_{W^{1,1}(\Omega)} = \alpha_1 \left\|v_h^{q,m} - w_h^{q,m}\right\|_{W^{1,1}(\Omega)},\end{aligned} \tag{126}$$

where, in the last inequality, we used the Poincaré-like inequality (120) and $\alpha_1 = \frac{1}{C_p}$ with C_p independent of Δ, which is the intermediate result that we wanted to show.

In order to get the final result, it suffices to observe that the previous inequality is uniform with respect to the indices q and m, so, we can take the infinity norm on these indices of both the sides and get

$$\left\| \mathcal{L}^1_\Delta(\underline{\boldsymbol{v}}) - \mathcal{L}^1_\Delta(\underline{\boldsymbol{w}}) \right\|_Y \geq \alpha_1 \left\| \underline{\boldsymbol{v}} - \underline{\boldsymbol{w}} \right\|_X \tag{127}$$

using the definitions (117) and (119). $\square$

Proposition 3.11 (Lipschitz-continuity-like condition of $\mathcal{L}^1_\Delta - \mathcal{L}^2_\Delta$)**.** *Let $\mathcal{L}^1_\Delta, \mathcal{L}^2_\Delta : X \to Y$ the operators defined in* (110) *and* (99)*. Consider $\underline{\boldsymbol{v}}, \underline{\boldsymbol{w}} \in X$ regular enough and suppose that assumption 3.9 holds. Then, $\exists \alpha_2 > 0$ independent of Δ s.t.*

$$\left\| \left[\mathcal{L}^1_\Delta(\underline{\boldsymbol{v}}) - \mathcal{L}^2_\Delta(\underline{\boldsymbol{v}}) \right] - \left[\mathcal{L}^1_\Delta(\underline{\boldsymbol{w}}) - \mathcal{L}^2_\Delta(\underline{\boldsymbol{w}}) \right] \right\|_Y \leq \alpha_2 \Delta \left\| \underline{\boldsymbol{v}} - \underline{\boldsymbol{w}} \right\|_X . \tag{128}$$

Proof. Focusing on one DoF $i \in \{1, \dots, I\}$ and on one subtimenode $m \in \{1, \dots, M\}$, we have

$$\begin{aligned} &\left[\mathcal{L}^{1,m}_{\Delta,i}(\underline{\boldsymbol{v}}) - \mathcal{L}^{2,m}_{\Delta,i}(\underline{\boldsymbol{v}}) \right] - \left[\mathcal{L}^{1,m}_{\Delta,i}(\underline{\boldsymbol{w}}) - \mathcal{L}^{2,m}_{\Delta,i}(\underline{\boldsymbol{w}}) \right] = \\ &C_i \left(\boldsymbol{v}^m_i - \boldsymbol{w}^m_i \right) - \sum_{K \in K_i} \sum_{\boldsymbol{x}_j \in K} \left(\boldsymbol{v}^m_j - \boldsymbol{w}^m_j \right) \int_K \varphi_i(\boldsymbol{x}) \varphi_j(\boldsymbol{x}) d\boldsymbol{x} - \Delta t \sum_{\ell=0}^M \theta^m_\ell \left[\boldsymbol{\phi}_i(\boldsymbol{v}^\ell) - \boldsymbol{\phi}_i(\boldsymbol{w}^\ell) \right] . \end{aligned} \tag{129}$$

Just like we did when we proved the coercivity-like property of $\mathcal{L}^1_\Delta$, we will work on the single component of (129) for fixed $q = 1, \dots, Q$ and $m = 1, \dots, M$, then we will derive the final result on the norms of X and Y by considering the ∞-norm over the indices q and m.

Let us thus focus on

$$\begin{aligned} &\left[\mathcal{L}^{1,q,m}_{\Delta,i}(\underline{\boldsymbol{v}}) - \mathcal{L}^{2,q,m}_{\Delta,i}(\underline{\boldsymbol{v}}) \right] - \left[\mathcal{L}^{1,q,m}_{\Delta,i}(\underline{\boldsymbol{w}}) - \mathcal{L}^{2,q,m}_{\Delta,i}(\underline{\boldsymbol{w}}) \right] = C_i \left(v^{q,m}_i - w^{q,m}_i \right) \\ &- \sum_{K \in K_i} \sum_{\boldsymbol{x}_j \in K} \left(v^{q,m}_j - w^{q,m}_j \right) \int_K \varphi_i(\boldsymbol{x}) \varphi_j(\boldsymbol{x}) d\boldsymbol{x} - \Delta t \sum_{\ell=0}^M \theta^m_\ell \left[\phi^q_i(\boldsymbol{v}^\ell) - \phi^q_i(\boldsymbol{w}^\ell) \right] \end{aligned} \tag{130}$$

where $\phi^q_i(\cdot)$ represents the q-th component of the space residual $\boldsymbol{\phi}_i(\cdot)$. We want to show now that the 1-norm, over all the indices i, of (130), for fixed q and m, is such that

$$\left\| \left[\mathcal{L}^{1,q,m}_\Delta(\underline{\boldsymbol{v}}) - \mathcal{L}^{2,q,m}_\Delta(\underline{\boldsymbol{v}}) \right] - \left[\mathcal{L}^{1,q,m}_\Delta(\underline{\boldsymbol{w}}) - \mathcal{L}^{2,q,m}_\Delta(\underline{\boldsymbol{w}}) \right] \right\|_{1,I} \leq \alpha_2 \Delta \left\| \underline{\boldsymbol{v}} - \underline{\boldsymbol{w}} \right\|_X , \tag{131}$$

for some α_2 independent of Δ, from which we will get the final result by taking the infinity norm of the left hand side with respect to the indices q and m. Thanks to the triangular inequality we

have

$$\left\| \left[\mathcal{L}_\Delta^{1,q,m}(\underline{\boldsymbol{v}}) - \mathcal{L}_\Delta^{2,q,m}(\underline{\boldsymbol{v}}) \right] - \left[\mathcal{L}_\Delta^{1,q,m}(\underline{\boldsymbol{w}}) - \mathcal{L}_\Delta^{2,q,m}(\underline{\boldsymbol{w}}) \right] \right\|_{1,I} \tag{132}$$

$$= \sum_{i=1}^{I} \left| \left[\mathcal{L}_{\Delta,i}^{1,q,m}(\underline{\boldsymbol{v}}) - \mathcal{L}_{\Delta,i}^{2,q,m}(\underline{\boldsymbol{v}}) \right] - \left[\mathcal{L}_{\Delta,i}^{1,q,m}(\underline{\boldsymbol{w}}) - \mathcal{L}_{\Delta,i}^{2,q,m}(\underline{\boldsymbol{w}}) \right] \right| \tag{133}$$

$$\leq \underbrace{\sum_{i=1}^{I} \left| \sum_{K \in K_i} \left(v_i^{q,m} - w_i^{q,m}\right) \int_K \varphi_i(\boldsymbol{x}) d\boldsymbol{x} - \sum_{K \in K_i} \sum_{\boldsymbol{x}_j \in K} \left(v_j^{q,m} - w_j^{q,m}\right) \int_K \varphi_i(\boldsymbol{x}) \varphi_j(\boldsymbol{x}) d\boldsymbol{x} \right|}_{=:E_1} \tag{134}$$

$$+ \underbrace{\sum_{i=1}^{I} \left| \Delta t \sum_{\ell=0}^{M} \theta_\ell^m \left[\phi_i^q(\boldsymbol{v}^\ell) - \phi_i^q(\boldsymbol{w}^\ell) \right] \right|}_{=:E_2} \tag{135}$$

recalling the definition of $C_i = \sum_{K \in K_i} \int_K \varphi(\boldsymbol{x}) d\boldsymbol{x}$ in (103).

Thanks to the previous inequality, we can deal separately with the two terms of the right hand side, the first one (134) concerning the mass matrix and the second one (135) involving the space residuals, and show that they can be bounded in the following way

$$E_1 \leq C_a \Delta \left\| \underline{\boldsymbol{v}} - \underline{\boldsymbol{w}} \right\|_X, \tag{136}$$

$$E_2 \leq C_b \Delta \left\| \underline{\boldsymbol{v}} - \underline{\boldsymbol{w}} \right\|_X, \tag{137}$$

with C_a and C_b independent of Δ which would give us the desired result.

• First term concerning the mass matrix

In order to bound this term, we can directly apply the preliminary result in proposition 3.4 and we get

$$\begin{aligned} &\left| \sum_{K \in K_i} \left(v_i^{q,m} - w_i^{q,m}\right) \int_K \varphi_i(\boldsymbol{x}) d\boldsymbol{x} - \sum_{K \in K_i} \sum_{\boldsymbol{x}_j \in K} \left(v_j^{q,m} - w_j^{q,m}\right) \int_K \varphi_i(\boldsymbol{x}) \varphi_j(\boldsymbol{x}) d\boldsymbol{x} \right| \\ &\leq \hat{C} \Delta C_i \left\| \left\| \nabla_{\boldsymbol{x}} \left(v_h^{q,m} - w_h^{q,m}\right) \right\|_1 \right\|_{L^\infty(\mathcal{K}_i)}, \quad \forall i = 1, \dots, I, \end{aligned} \tag{138}$$

with $\hat{C}$ independent of the mesh parameter $\Delta = h$, dependent just on the number of dimensions D, on the degree M and on the type of the elements in the mesh. From (138) we have

$$\begin{aligned} E_1 &= \sum_{i=1}^{I} \left| \sum_{K \in K_i} \left(v_i^{q,m} - w_i^{q,m}\right) \int_K \varphi_i(\boldsymbol{x}) d\boldsymbol{x} - \sum_{K \in K_i} \sum_{\boldsymbol{x}_j \in K} \left(v_j^{q,m} - w_j^{q,m}\right) \int_K \varphi_i(\boldsymbol{x}) \varphi_j(\boldsymbol{x}) d\boldsymbol{x} \right| \\ &\leq \hat{C} \Delta \sum_{i=1}^{I} \left\| \left\| \nabla_{\boldsymbol{x}} \left(v_h^{q,m} - w_h^{q,m}\right) \right\|_1 \right\|_{L^\infty(\mathcal{K}_i)} C_i. \end{aligned} \tag{139}$$

Thanks to proposition 3.6 taking $z = \left\| v_h^{q,m} - w_h^{q,m} \right\|_1$, then (139) can be bounded in the following way

$$\hat{C} \Delta \sum_{i=1}^{I} \left\| \left\| \nabla_{\boldsymbol{x}} \left(v_h^{q,m} - w_h^{q,m}\right) \right\|_1 \right\|_{L^\infty(\mathcal{K}_i)} C_i \leq \hat{C} \Delta \tilde{C}^* \left\| \left\| \nabla_{\boldsymbol{x}} \left(v_h^{q,m} - w_h^{q,m}\right) \right\|_1 \right\|_{L^1(\Omega)}. \tag{140}$$

Hence, by definition of the $W^{1,1}(\Omega)$-norm (115), of the $W_I^{1,1}(\Omega)$-norm (116) and of the X norm (117), we have

$$\begin{aligned} E_1 &\le \hat{C}\Delta\tilde{C}^* \left\| \|\nabla_{\boldsymbol{x}} (v_h^{q,m} - w_h^{q,m})\|_1 \right\|_{L^1(\Omega)} \le \hat{C}\tilde{C}^*\Delta \|v_h^{q,m} - w_h^{q,m}\|_{W^{1,1}(\Omega)} \\ &\le C_a\Delta \|v_h^{q,m} - w_h^{q,m}\|_{W^{1,1}(\Omega)} \le C_a\Delta \|\underline{\boldsymbol{v}} - \underline{\boldsymbol{w}}\|_X , \end{aligned} \tag{141}$$

with $C_a = \hat{C}\tilde{C}^*$ independent of Δ.

• **Second term involving the space residuals**
By applying the triangular inequality, recalling that θ_ℓ^m are fixed normalized constant coefficients, thus, bounded in absolute value by a positive constant C_θ, and that $\Delta t \le Ch = C\Delta$ for some fixed constant C, we have

$$E_2 = \sum_{i=1}^{I} \left| \Delta t \sum_{\ell=0}^{M} \theta_\ell^m \left[\phi_i^q(\boldsymbol{v}^\ell) - \phi_i^q(\boldsymbol{w}^\ell) \right] \right| \le \Delta C C_\theta \sum_{i=1}^{I}\sum_{\ell=0}^{M} |\phi_i^q(\boldsymbol{v}^\ell) - \phi_i^q(\boldsymbol{w}^\ell)|. \tag{142}$$

From the fact that $\boldsymbol{v}^0 = \boldsymbol{w}^0 = \boldsymbol{c}^0$, we have

$$\Delta C C_\theta \sum_{i=1}^{I}\sum_{\ell=0}^{M} |\phi_i^q(\boldsymbol{v}^\ell) - \phi_i^q(\boldsymbol{w}^\ell)| \le \Delta C C_\theta M \sum_{i=1}^{I} \left\| \{\phi_i^q(\boldsymbol{v}^m) - \phi_i^q(\boldsymbol{w}^m)\}_{\substack{q=1,\dots,Q\\ m=1,\dots,M}} \right\|_{\infty,Q,M} . \tag{143}$$

Then, we use the assumption of smoothness of the space residuals $\boldsymbol{\phi}_i(\cdot)$. In particular, we assume the following Lipschitz-continuity-like condition

$$\sum_{i=1}^{I} \left\| \{\phi_i^q(\boldsymbol{v}^m) - \phi_i^q(\boldsymbol{w}^m)\}_{\substack{q=1,\dots,Q\\ m=1,\dots,M}} \right\|_{\infty,Q,M} \le C_\phi \left\| \|\underline{\boldsymbol{v}}_h - \underline{\boldsymbol{w}}_h\|_{W^{1,1}(\Omega)} \right\|_{\infty,Q,M} = C_\phi \|\underline{\boldsymbol{v}} - \underline{\boldsymbol{w}}\|_X \tag{144}$$

with C_ϕ independent of Δ. Using this, from (143) we get

$$E_2 \le C_b\Delta \|\underline{\boldsymbol{v}} - \underline{\boldsymbol{w}}\|_X \tag{145}$$

with $C_b = CC_\theta M C_\phi$ independent of Δ, obtaining (137).

Now, that we have proven (136) and (137), the Lipschitz inequality (131) is proven with $\alpha_2 = C_a + C_b$ independent of Δ. Finally, we get the final result by observing that what we have proved holds for any component with fixed indices $q = 1, \dots, Q$ and $m = 1, \dots, M$. So, applying the infinity norm of the left hand side with respect to these indices, we get

$$\begin{aligned} \max_{q,m} &\left\| \left[\mathcal{L}_\Delta^{1,q,m}(\underline{\boldsymbol{v}}) - \mathcal{L}_\Delta^{2,q,m}(\underline{\boldsymbol{v}}) \right] - \left[\mathcal{L}_\Delta^{1,q,m}(\underline{\boldsymbol{w}}) - \mathcal{L}_\Delta^{2,q,m}(\underline{\boldsymbol{w}}) \right] \right\|_{1,I} \\ &= \left\| \left[\mathcal{L}_\Delta^{1}(\underline{\boldsymbol{v}}) - \mathcal{L}_\Delta^{2}(\underline{\boldsymbol{v}}) \right] - \left[\mathcal{L}_\Delta^{1}(\underline{\boldsymbol{w}}) - \mathcal{L}_\Delta^{2}(\underline{\boldsymbol{w}}) \right] \right\|_Y \le \alpha_2\Delta \|\underline{\boldsymbol{v}} - \underline{\boldsymbol{w}}\|_X , \end{aligned} \tag{146}$$

which is the thesis. □

3.2 Issues with the DeC for CG

We discuss here a negative result seen in the numerical tests even on the monodimensional linear advection equation (LAE) reported in the main document and in many other works, e.g. [1, 7, 2]. The DeC formulation for PDEs with the lumping of the mass matrix does not give the expected formal order of accuracy for space discretizations of order higher than or equal to 4 if one performs the theoretical optimal number of iterations. In this section, we will try to investigate the problem by numerically assessing the impact of the number of iterations P, of the CFL and of the CIP stabilization on higher order derivatives. Before starting, we remark that the loss in the accuracy is not registered in the context of steady problems, indeed, in [3] the expected order of accuracy is obtained with B3 on a nontrivial steady test for the bidimensional Euler equations. Further, one of the authors is involved in a project [5] on some novel CIP stablizations for the monodimensional SW equations, soon to be submitted, in which the right order of accuracy is obtained for P3, B3 and B4 with the theoretical optimal number of iterations on all the considered steady tests. Therefore, we will focus on the same unsteady test for the monodimensional LAE presented in the main document and, in particular, we will consider P3, B3 and B4 as basis functions and the original formulation of the bDeC for PDEs without interpolations between the iterations as timestepping method. For P3 and B3 we will use, in the context of the CIP stabilization, the same coefficients adopted in the main document, $\delta^{\text{CIP}} = 0.00702$. As the optimal coefficient for B4 is not provided in [6], we will adopt the same coefficient as for B3 and P3. The reference CFL adopted for the tests with B3 and P3 is 0.1, instead, with B4 it is 0.05. Where not specified, such values have been adopted.

3.2.1 Impact of the number of iterations

The numerical results for different number of iterations are reported in fig. 1. In all the cases we can see the same trend: the optimal number of iterations gives order 2, increasing the number of iterations improves the accuracy allowing to reach the formal order. Nevertheless, it is important to notice that many more iterations, with respect to the optimal number, are needed in order to achieve the right order of convergence: 10 for P3, 80 for B3, 320 for B4.

3.2.2 Impact of the CFL

The numerical results for different values of the CFL are reported in fig. 2. Such parameter seems not to have impact on the order. For P3, CFL = 0.1 performs better than CFL = 0.01 and CFL = 0.001; for the other basis functions one gets similar results for the different values of the CFL meaning that spatial error is dominating with respect to the error in time.

3.2.3 Impact of the stabilization on higher order derivatives

The CIP stabilization on the first derivative that we have presented can be actually generalized to keep into account higher order derivatives as in [4]

$$\boldsymbol{ST}_i(\boldsymbol{u}_h) = \sum_{f \in \mathcal{F}_h} \sum_{r=1}^{R} \alpha_{f,r}^{\text{CIP}} \int_f \left[\!\left[\nabla_{\nu_f}^r \varphi_i \right]\!\right] \cdot \left[\!\left[\nabla_{\nu_f}^r \boldsymbol{u}_h \right]\!\right] d\sigma(\boldsymbol{x}), \quad \alpha_{f,r}^{\text{CIP}} = \delta_r^{\text{CIP}} \bar{\rho}_f h_f^{2r} \tag{147}$$

where $\mathcal{F}_h$ is the set of the $(D-1)$-dimensional faces shared by two elements of $\mathcal{T}_h$, $\nabla_{\nu_f}^r$ is the r-th partial derivative in the direction ν_f normal to the face f and δ_r^{CIP} are constant parameters which

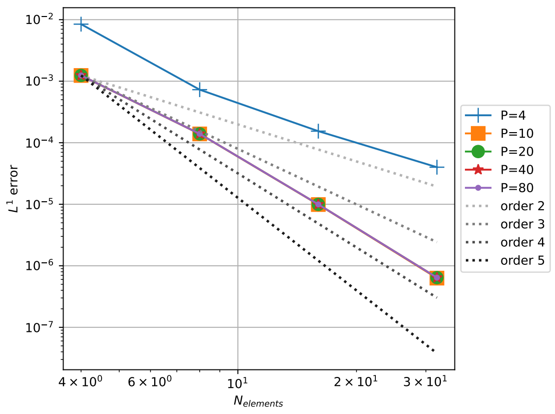

(a) P3

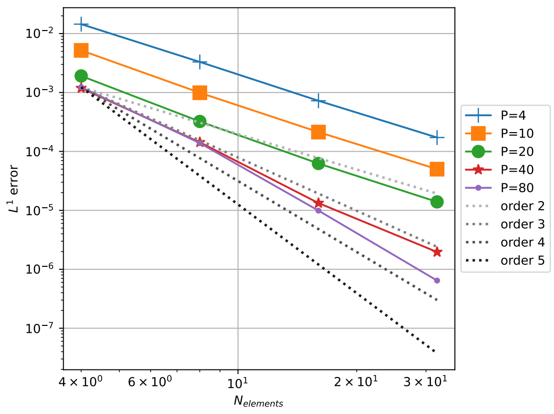

(b) B3

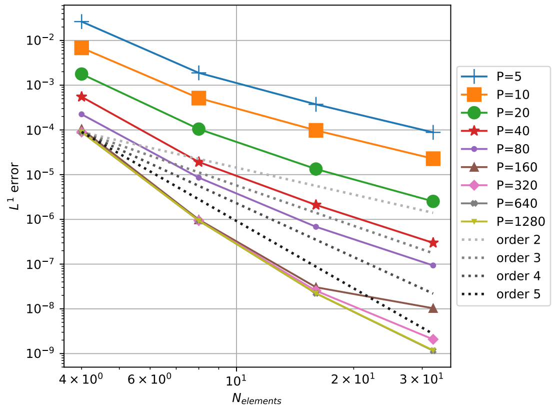

(c) B4

Figure 1: 1D LAE: tests with different numbers of iterations

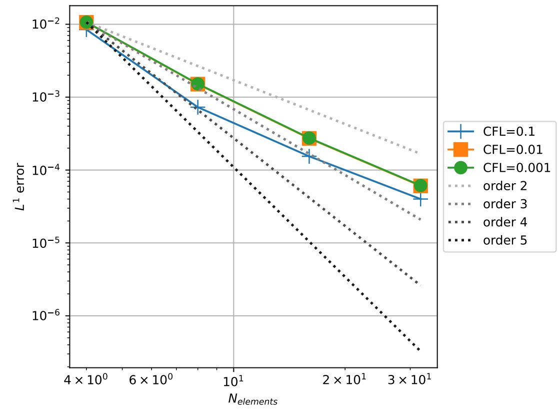

(a) P3

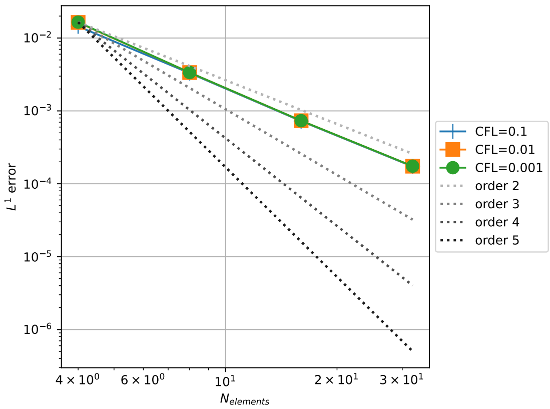

(b) B3

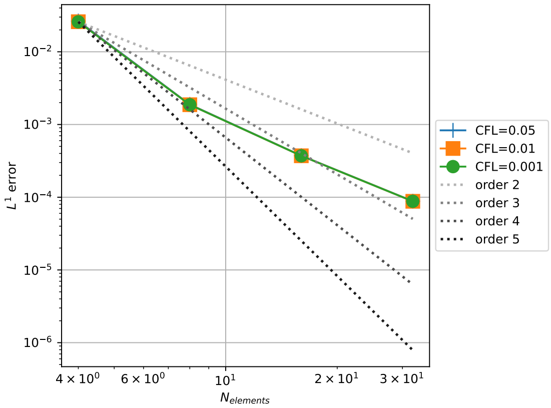

(c) B4

Figure 2: 1D LAE: tests with different CFLs

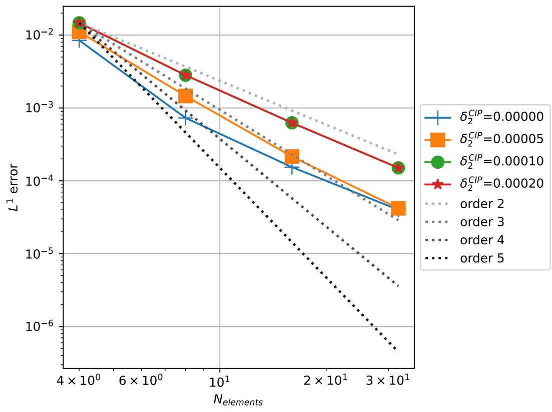

(a) P3.

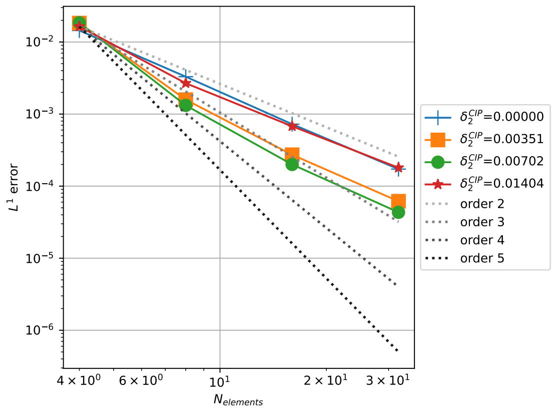

(b) B3.

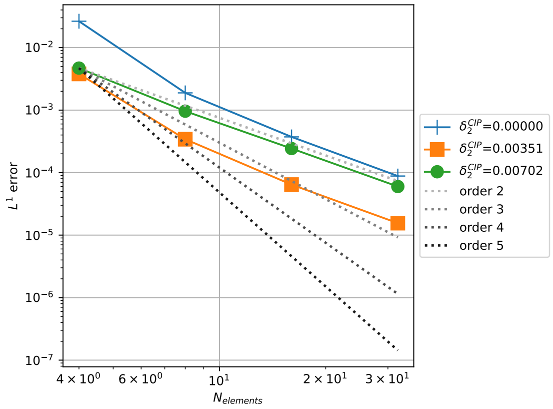

(c) B4.

Figure 3: 1D LAE: tests with different stabilization parameters on the second derivative.

must be tuned. We will focus on the stabilization of the first and second derivatives only, $R = 2$. The results obtained with $\delta_1^{\text{CIP}} = 0.00702$ and different values of δ_2^{CIP} are displayed in fig. 3. For B3 and B4, the extra stabilization seems to help in decreasing the errors but still it is not sufficient to achieve the right order of accuracy.

3.2.4 Final remarks

The conclusion of the previous analysis is that, in the context of unsteady problems, with the optimal number of iterations one obtains second order accuracy. Among the three aspects numerically analyzed, only the first one seems to have an effect on the order of accuracy; in particular, many more iterations than expected are needed to reach the formal order. For the moment we do not further investigate this issue, but we have other ideas on how to proceed. First of all, an analysis of the combination of the three parameters studied above could give better results and a linear stability/dispersion analysis, in the style of [6, 7], can help in determining the optimal setting to

achieve the best possible results. Further, higher order derivatives stabilization terms could be taken in consideration hoping for a better stabilization, this has been suggested also in [2, 7]. More in general, other stabilizations other than CIP and OSS could be considered. Moreover, the authors suspect that assumption 3.8 is not verified by the approximations and a stronger estimate on the H^1 norm of the solution of the discrete problem should be provided with weaker hypotheses to guarantee the accuracy results.

We conclude this section remarking that the mentioned problem does not occur with cubature elements also in the DeC framework, which provide accurate and fast results.

4 Vibrating system

Let us consider a general sinusoidal function

$$x(t) = X \cos(\Omega t + \varphi), \tag{148}$$

then we refer to $X \in \mathbb{R}_0^+$ as the amplitude, to $\Omega \in \mathbb{R}^+$ as the frequency and to $\varphi \in [0, 2\pi[$ modulo $2\pi n$ with $n \in \mathbb{Z}$ as the phase.

Let us introduce two general sinusoidal functions

$$x_j(t) = X_j \cos(\Omega t + \varphi_j), \quad \text{for } j = 1, 2, \tag{149}$$

characterized by the same frequency $\Omega > 0$, amplitudes $X_1, X_2 \geq 0$ and phases $\varphi_1, \varphi_2 \in [0, 2\pi[$ modulo $2n\pi$ with $n \in \mathbb{Z}$.

Proposition 4.1. *The sum $x_s(t) = x_1(t) + x_2(t)$ between two sinusoidal functions with the same frequency Ω is another sinusoidal function with the same frequency.*

Proof. If $x_1(t)+x_2(t) = 0$ or at least one between X_1 or X_2 is zero, then the proof is straightforward so let us focus on the case in which $x_1(t) + x_2(t) \neq 0$ and both X_1 and X_2 are different from 0.

From basic trigonometry, we have

$$x_j(t) = X_j \cos(\Omega t + \varphi_j) = X_j[\cos(\Omega t) \cos(\varphi_j) - \sin(\Omega t) \sin(\varphi_j)], \quad \text{for } j = 1, 2, \tag{150}$$

then

$$x_s(t) = x_1(t) + x_2(t) = A \cos(\Omega t) - B \sin(\Omega t), \tag{151}$$

$$\text{with } A := X_1 \cos(\varphi_1) + X_2 \cos(\varphi_2), \qquad B := X_1 \sin(\varphi_1) + X_2 \sin(\varphi_2). \tag{152}$$

We consider now the point $(A, B) \in \mathbb{R}^2$, different from $(0, 0)$ by assumption, and the induced vector of length $X_s = \sqrt{A^2 + B^2}$ and phase $\varphi_s = \angle(A, B)$, so that $A = X_s \cos(\varphi_s)$ and $B = X_s \sin(\varphi_s)$. By definition of such vector, (151) can be recast as

$$x_1(t) + x_2(t) = X_s \cos(\varphi_s) \cos(\Omega t) - X_s \sin(\varphi_s) \sin(\Omega t) = X_s \cos(\Omega t + \varphi_s), \tag{153}$$

which completes the proof. □

We introduce now a bijection $\mathcal{S}$ from the quotient set of the sinusoidal functions with a fixed frequency Ω defined by (X, φ), in which we identify all the functions characterized by $X = 0$, onto the complex plane

$$\mathcal{S}(x(t)) = \mathcal{S}(X, \varphi) = \begin{cases} Xe^{i\varphi} & \text{if} \quad X \neq 0 \\ 0 & \text{if} \quad X = 0 \end{cases}. \tag{154}$$

The complex number $\overline{X} := \mathcal{S}(x(t))$ is called phasor associated to the sinusoidal function $x(t)$.

Proposition 4.2. *If we have two sinusoidal functions $x_1(t), x_2(t)$ with the same frequency Ω then the phasor $\overline{X}_s$ associated to the sum $x_s(t)$ of the two sinusoidal functions is the sum of the phasors $\overline{X}_1, \overline{X}_2$ associated to the single sinusoidal functions.*

Proof. The phasors related to the sinusoidal functions (149) are given by

$$\overline{X}_j = X_j e^{i\varphi_j} = X_j \left[\cos(\varphi_j) + i \sin(\varphi_j)\right], \text{ for } j = 1, 2. \tag{155}$$

If one between X_1 or X_2 is zero then the proof is straightforward therefore we focus on the case in which they are both different from 0. Further, we assume for the moment that $x_1(t) + x_2(t) \neq 0$. The sum of the phasors gives

$$\begin{aligned} \overline{X}_r &= \overline{X}_1 + \overline{X}_2 \\ &= \left[X_1 \cos(\varphi_1) + X_2 \cos(\varphi_2)\right] + i\left[X_1 \sin(\varphi_1) + X_2 \sin(\varphi_2)\right] = A + iB \end{aligned} \tag{156}$$

with A and B defined exactly as in (152) leading to

$$\overline{X}_r = X_r e^{i\varphi_r} \tag{157}$$

with $X_r = X_s$ and $\varphi_r = \varphi_s$ with X_s and φ_s defined from the phasor associated to $x_s(t)$.

If $x_1(t) + x_2(t) = 0$, by simple considerations, we must have $X_2 = X_1$ and $\varphi_2 = \varphi_1 + \pi$ modulo 2π, which leads to

$$\overline{X}_1 = X_1 e^{i\varphi_1}, \quad \overline{X}_2 = X_1 e^{i(\varphi_1 + \pi)} = -\overline{X}_1. \tag{158}$$

Then, we clearly have $\overline{X}_1 + \overline{X}_2 = 0$. Indeed, also the phasor $\overline{X}_s$ associated to the sum is 0 and this completes the proof. □

It is clear that if we have a sinusoidal function $x(t) = X\cos(\Omega t + \varphi)$ then its derivative in time is still a sinusoidal function with the same frequency

$$x'(t) = -\Omega X \sin(\Omega t + \varphi) = \Omega X \cos\left(\Omega t + \varphi + \frac{\pi}{2}\right). \tag{159}$$

Then the phasor $\overline{X'}$ associated to the derivative in time $x'(t)$ is

$$\overline{X'} = \Omega X e^{i\left(\varphi + \frac{\pi}{2}\right)} = i\Omega X e^{i\varphi} = i\Omega\overline{X}. \tag{160}$$

By the same argument we have that the phasor $\overline{X''}$ associated to the second derivative in time $x''(t)$ is

$$\overline{X''} = i\Omega\overline{X'} = i\Omega(i\Omega\overline{X}) = -\Omega^2\overline{X}. \tag{161}$$

We consider the scalar ODE

$$\begin{cases} my'' + ry' + ky = F\cos(\Omega t + \varphi), & t \in \mathbb{R}_0^+ \\ y(0) = A, \\ y'(0) = B, \end{cases} \tag{162}$$

with the real nonnegative constants $m, k, \Omega > 0$ and $r, F \geq 0$ with $\varphi \in [0, 2\pi[$ modulo $2\pi n$ with $n \in \mathbb{Z}$. The solution to (162) is given by

$$y(t) = y_h(t) + y_p(t) \tag{163}$$

where $y_h(t)$ is a solution to the homogeneus equation and $y_p(t)$ is a solution to the whole equation.

We first focus on the homogeneus problem

$$my'' + ry' + ky = 0 \tag{164}$$

and we look for a solution in the form $y(t) = Ae^{\lambda t}$ which is nontrivial and so we assume $A \neq 0$. We substitute it in the homogeneus equation and we get

$$\left(m\lambda^2 + r\lambda + k\right) Ae^{\lambda t} = 0 \tag{165}$$

and since $Ae^{\lambda t} \neq 0$ $\forall t \in \mathbb{R}_0^+$ because $A \neq 0$ then we get the characteristic equation

$$\lambda^2 + \alpha\lambda + \beta = 0 \tag{166}$$

with $\alpha = \frac{r}{m} \geq 0$ and $\beta = \frac{k}{m} > 0$. The roots are given by

$$\lambda_{1,2} = \frac{1}{2}\left(-\alpha \pm \sqrt{\alpha^2 - 4\beta}\right) \tag{167}$$

and, depending on the parameters of the problem, we have three possibilities

1. $\lambda_1 \neq \lambda_2$, real, negative and different if $\alpha > 2\sqrt{\beta} \Leftrightarrow r > 2\sqrt{km}$;
2. $\lambda_1 = \lambda_2 = \lambda$, real, negative and coincident if $\alpha = 2\sqrt{\beta} \Leftrightarrow r = 2\sqrt{km}$;
3. $\lambda_{1,2} = \alpha \pm i\omega$, complex and conjugate with negative real part if $\alpha < 2\sqrt{\beta} \Leftrightarrow r < 2\sqrt{km}$.

Thus, the solution to our homogeneous ODE is

$$y_h(t) = \begin{cases} C_1 e^{\lambda_1 t} + C_2 e^{\lambda_2 t}, & \text{if} \quad \alpha > 2\sqrt{\beta} \Leftrightarrow r > 2\sqrt{km}, \\ C_1 e^{\lambda t} + C_2 t e^{\lambda t}, & \text{if} \quad \alpha = 2\sqrt{\beta} \Leftrightarrow r = 2\sqrt{km}, \\ e^{-\frac{\alpha}{2}t}\left(C_1 cos(\omega t) + C_2 sin(\omega t)\right), & \text{if} \quad \alpha < 2\sqrt{\beta} \Leftrightarrow r < 2\sqrt{km}. \end{cases} \tag{168}$$

Now, we focus on the whole ODE (162) and we assume a sinusoidal solution of the type $y_p = Y_p \cos(\Omega t + \psi)$, we substitute it in (162) and we solve the equation in the space of the phasors. Recalling the expression of the phasors associated to the first and the second derivatives of a sinusoidal function given by (160) and (161) we have

$$-m\Omega^2 \overline{Y}_p + i\Omega r \overline{Y}_p + k\overline{Y}_p = Fe^{i\varphi}. \tag{169}$$

Then

$$\overline{Y}_p = \frac{Fe^{i\varphi}}{-m\Omega^2 + k + i\Omega r}, \tag{170}$$

from which we get

$$Y_p = \frac{F}{\sqrt{(-m\Omega^2 + k)^2 + \Omega^2 r^2}}, \qquad \psi = \varphi - \arg\left(-m\Omega^2 + k + i\Omega r\right), \tag{171}$$

where by $\arg(\cdot)$ we denote the phase of the argument up to $2n\pi$ with $n \in \mathbb{Z}$. Once we compute $\overline{Y}_p$, we automatically get the unique associated sinusoidal function $y_p(t) = Y_p \cos(\Omega t + \psi)$.

So, the final solution to our ODE (162) is $y(t) = y_h(t) + y_p(t)$, where $y_h(t)$ is given by (168) and $y_p(t)$ is a sinusoidal function whose amplitude and phase are given by (171).

The two constants C_1 and C_2 in $y_h(t)$ are computed by imposing the initial conditions $y(0) = A$ and $y'(0) = B$ and solving the resulting 2 by 2 linear system.

References

[1] Rémi Abgrall. High order schemes for hyperbolic problems using globally continuous approximation and avoiding mass matrices. *J. Sci. Comput.*, 73(2-3):461–494, 2017.

[2] Rémi Abgrall, Paola Bacigaluppi, and Svetlana Tokareva. High-order residual distribution scheme for the time-dependent Euler equations of fluid dynamics. *Computers & Mathematics with Applications*, 78(2):274–297, 2019.

[3] Rémi Abgrall and Davide Torlo. High order asymptotic preserving deferred correction implicit-explicit schemes for kinetic models. *SIAM Journal on Scientific Computing*, 42(3):B816–B845, 2020.

[4] Mats G Larson and Sara Zahedi. Stabilization of high order cut finite element methods on surfaces. *IMA Journal of Numerical Analysis*, 40(3):1702–1745, 2020.

[5] Lorenzo Micalizzi, Mario Ricchiuto, and Rémi Abgrall. Novel well-balanced arbitrary high order continuous interior penalty stabilization techniques for continuous galerkin fem and residual distribution. *in preparation*, 2022.

[6] Sixtine Michel, Davide Torlo, Mario Ricchiuto, and Rémi Abgrall. Spectral analysis of continuous FEM for hyperbolic PDEs: influence of approximation, stabilization, and time-stepping. *Journal of Scientific Computing*, 89(2):1–41, 2021.

[7] Sixtine Michel, Davide Torlo, Mario Ricchiuto, and Rémi Abgrall. Spectral analysis of high order continuous fem for hyperbolic pdes on triangular meshes: influence of approximation, stabilization, and time-stepping. *Journal of Scientific Computing*, 94(3):49, 2023.
